# Platinum-Catalyzed Diboration of Alkynes by 1,8-Diaminonaphthalene-Protected Diboronic Acid (B_2_(dan)_2_)

**DOI:** 10.1021/acs.joc.4c01939

**Published:** 2024-10-25

**Authors:** Shinichi Saito, Yuya Koizumi, Yuki Ito, Taiga Yasuda, Yusuke Yoshigoe

**Affiliations:** Department of Chemistry, Faculty of Science, Tokyo University of Science, Kagurazaka, Shinjuku, Tokyo 162-8601, Japan

## Abstract

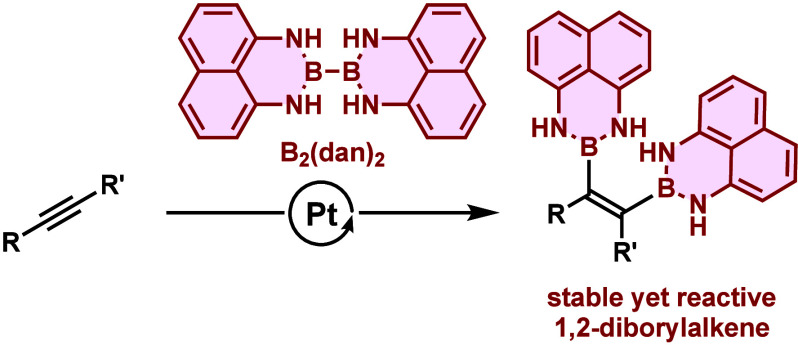

The diboration of alkynes by 1,8-diaminonaphthalene-protected diboronic acid (B_2_(dan)_2_) proceeded smoothly in the presence of a platinum catalyst, and 1,2-diborylalkenes were isolated in up to 94% yield. The use of an appropriate solvent and ligand was critical for the progress of the reaction. The derivatization of 1,2-diborylalkenes was briefly examined.

The transition metal-catalyzed addition of diboronic acid derivatives to unsaturated hydrocarbons is a useful method for the synthesis of organoboron compounds.^1,2^ Since the first publication^3^ by Miyaura, Suzuki, and co-workers reporting the Pt-catalyzed reaction of alkynes with bis(pinacolato)diboron (B_2_(pin)_2_), various combinations of diboronic acid derivatives, unsaturated hydrocarbons and metal catalysts have been examined. As for the diboration of alkynes, B_2_(pin)_2_ and bis(catecholato)diboron (B_2_(cat)_2_) were widely used as borylating agents, and a large number of 1,2-diboration has been reported (Scheme 1). The use of unsymmetrically substituted diboron compounds such as B(pin)B(dan) (dan = naphthalene-1,8-diaminato) provided a practical method for the synthesis of diborylated compounds which could be further functionalized in a selective manner.^4−6^ The Pt-catalyzed diboration of alkenes and allenes, including stereoselective reactions, has also been studied extensively by Morken and other groups.^7^ By choosing appropriate reaction conditions, the 1,1-diboration of alkynes proceeded, providing synthetically useful 1,1-diborylalkenes.^8^

**Scheme 1:**
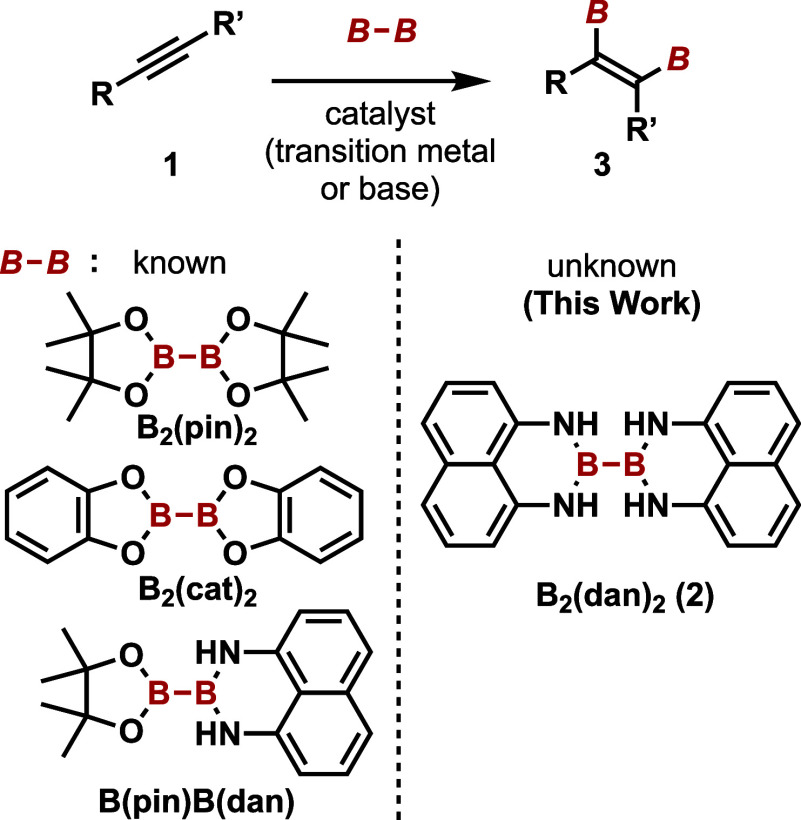
1,2-Diboration of Alkynes by Diboron Compounds

We have been interested in the chemistry of B(dan) derivatives.^9^ B(dan) derivatives were introduced as protected (masked) boronic acids by Suginome and co-workers,^10^ but recent studies disclosed that B(dan) group could be activated and used for cross-coupling reactions without removal of the diaminonaphthalene moiety.^9,11^ We recently reported that B_2_(dan)_2_ (**2**) is readily available from diboronic acid. B_2_(dan)_2_ is a bench stable compound and could be used for the borylation of styrene derivatives in the presence of Cu catalyst.^9c^ Interestingly, B_2_(dan)_2_ has never been used for the diboration reaction of unsaturated hydrocarbons.

We envisioned that the use of B_2_(dan)_2_ in the diboration reaction of unsaturated hydrocarbons could provide stable yet reactive diboryl compounds. In this paper we report the synthesis of 1,2-diborylalkenes by the Pt-catalyzed diboration of alkynes.

We initiated our study by the screening of the Pt catalysts for this reaction. The diboration of 4-methoxyphenylacetylene (**1a**) with B_2_(dan)_2_ (**2**) was examined and the results are summarized in Table 1.

**Table 1:**
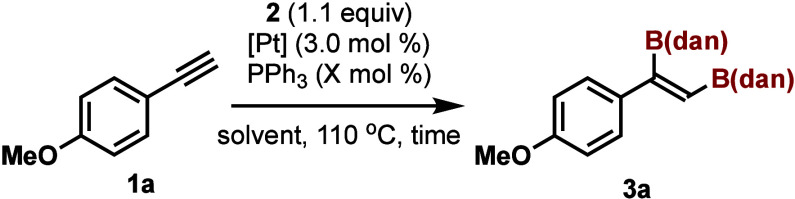
Optimization Study^a^

Entry	[Pt] (3 mol %)	PPh_3_ (mol %)	Solvent	t (h)	Yield^b^ (%)
1	Pt(PPh_3_)_4_	0	DMF	25	0
2	Pt(PPh_3_)_4_	0	toluene	23	78
3	Pt(dba)_3_	3.0	DMF	24	0
4	Pt(dba)_3_	3.0	toluene	0.5	84
5	Pt(dba)_3_	6.0	toluene	3	84
6	Pt(dba)_3_	9.0	toluene	4	79
7	Pt(dba)_3_	0	toluene	0.5	(48)^c^

a A mixture of **1a** (1.0 equiv), **2** (1.1 equiv), and [Pt] (3 mol %) in solvent was stirred for at 110 °C.

b Isolated yield.

c The product was not isolated in pure form.

The progress of the reaction was not observed when we applied the conditions reported by Miyaura and Suzuki: **1a** did not react with **2** at 110 °C in DMF (24 h) when Pt(PPh_3_)_4_ (3 mol %) was used as the catalyst (entry 1). The reaction proceeded slowly when toluene was used and the diborated compound (**3a**) was isolated in 78% yield after heating the mixture for 23 h (entry 2). Only *cis*-1,2-addition proceeded and the formation of other isomers was not detected. To examine the effect of the amount and structure of the ligand, we chose Pt(dba)_3_ (dba = bis(dibenzylideneacetone)) as the Pt source and used 3 mol % of PPh_3_. Although the reaction did not proceed in DMF, the reaction completed in 0.5 h and **3a** was isolated in 84% yield when the solvent was switched to toluene (entries 3 and 4). The observed solvent effect contrasted with the results reported previously in the reaction of B_2_(pin)_2_, where DMF was the best solvent.^3,4a^ When we increased the amount of PPh_3_, the rate of the reaction decreased (entries 5 and 6). The reaction proceeded in the absence of PPh_3_ but the yield of the product was low (entry 7). The results imply that the coordination of multiple phosphine ligands or the solvent to the Pt species would result in the decreased rate of the reaction. Next, we examined the effect of phosphine ligands on the reaction (Table S1). Other phosphine ligands including PPh_2_(*o*-Tol)^4c^ and (4-CF_3_C_6_H_4_)_3_P^4e^ were not effective for this reaction, and we selected the conditions described in Table 1, entry 4 for further studies. It is noteworthy that high reaction temperature was required for this reaction compared to reported reaction conditions for the Pt-catalyzed diboration of **1a** with B_2_(pin)_2_^3,4^ or B(pin)B(dan).^4e^ The result might imply the lower reactivity of B_2_(dan)_2_ compared to other diboryl compounds.^12^

We investigated the substrate scope of this reaction and the results are summarized in Schemes 2 and 3. The scalability of the reaction was demonstrated by using a larger amount (2.5 or 5.0 mmol) of **1a**, and **3a** was isolated in 66% or 77% yield (Scheme 2). The reaction of phenylacetylene (**1b**) and 4-dimethylaminophenylacetylene (**1c**) proceeded smoothly and the corresponding 1,2-diborylalkenes were isolated in 93% and 83% yields, respectively. The introduction of an electron-withdrawing group such as bromine (**1d**), trifluoromethyl group (**1e**), ethoxycarbonyl group (**1f**) or cyano group (**1g**) at the *para* position of the phenyl group did not significantly affect the reaction, and the products were isolated in 71–91% yields. 2-Methoxyphenylacetylene (**1h**) and 3-methoxyphenylacetylene (**3i**) reacted smoothly and gave the diborylated compounds in high yields. Even mesitylacetylene (**1j**) reacted under standard reaction conditions and **3j** was isolated in 83% yield. Unlike other alkenes substituted with B(pin) or B(cat), **3a**–**j** were bench stable compounds and they could be purified without precaution by column chromatography.

**Scheme 2:**
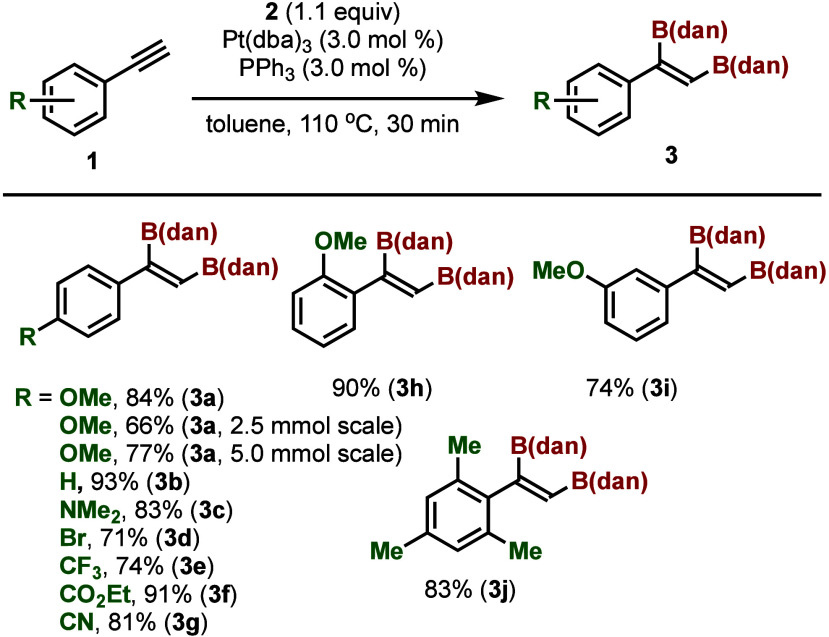
Pt-Catalyzed Diboration of Phenylacetylene Derivatives with B_2_(dan)_2_

**Scheme 3:**
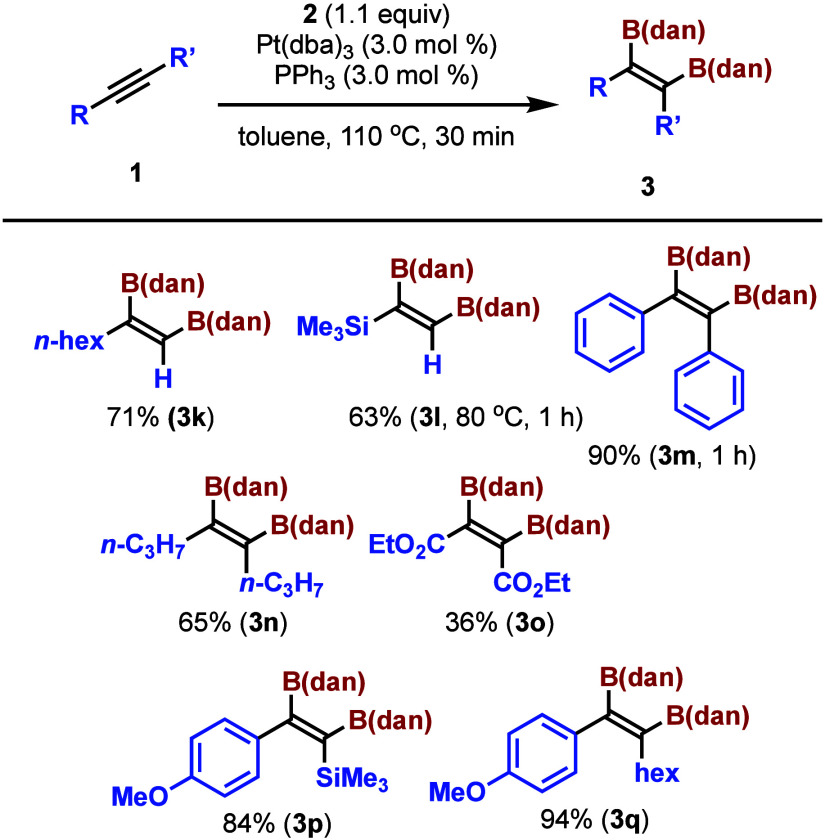
Pt-Catalyzed Diboration of Various Alkynes with B_2_(dan)_2_

The scope of this reaction was further examined using other alkynes (Scheme 3). 1-Octyne (**1k**) reacted under standard reaction conditions and the product was isolated in 71% yield. The reaction of trimethylsilylacetylene (**1l**) was examined at lower temperature (80 °C) due to the low bp (53 °C) of **1l**: the reaction completed in 1 h and **3l** was isolated in 63% yield. The reactivity of diphenylacetylene (**1m**) was lower compared to other alkynes: the reaction completed in 1 h and the diborylated compound was isolated in 90% yield. 4-Octyne (**1n**) was reactive toward the diboration reaction, and **3n** was isolated in 65% yield. Though diethyl acetylenedicarboxylate (**1o**) was reactive and gave the corresponding diborylated compound (**3o**), the yield of **3o** was low (36%). The reaction of a nonsymmetric internal alkyne gave the diborylated compound (**3p**) in 84% yield. An aryl alkyl alkyne reacted smoothly, providing the product (**3q**) in 94% yield. The results imply that this reaction would be widely applicable for a variety of alkynes.

We assume that the mechanism of this reaction is similar to that reported for the Pt-catalyzed diborylation of alkynes using other diboron compounds (Scheme 4). Thus, the PPh_3_-Pt(0) species (**I**) would be generated *in situ* by the reaction of Pt(dba)_3_ with PPh_3_, and the oxidative addition of the Pt(0) species with B_2_(dan)_2_ would provide the diboryl Pt(II) species (**II**). The insertion of alkyne to the Pt–B bond would provide the vinylplatinum intermediate (**III**). The reductive elimination of **III** would give the diborylated alkene and the PPh_3_-Pt(0) species would be generated. The presence of a large amount of the phosphine ligand or the use of polar solvent would inhibit the oxidative addition of **2** to the Pt(0) species and reduce the rate of the reaction. Acceleration of the oxidative addition of the Pt(0) species to B_2_(pin)_2_ in a less polar solvent (hexane) has been reported previously, supporting the assumption.^4a,13^

**Scheme 4:**
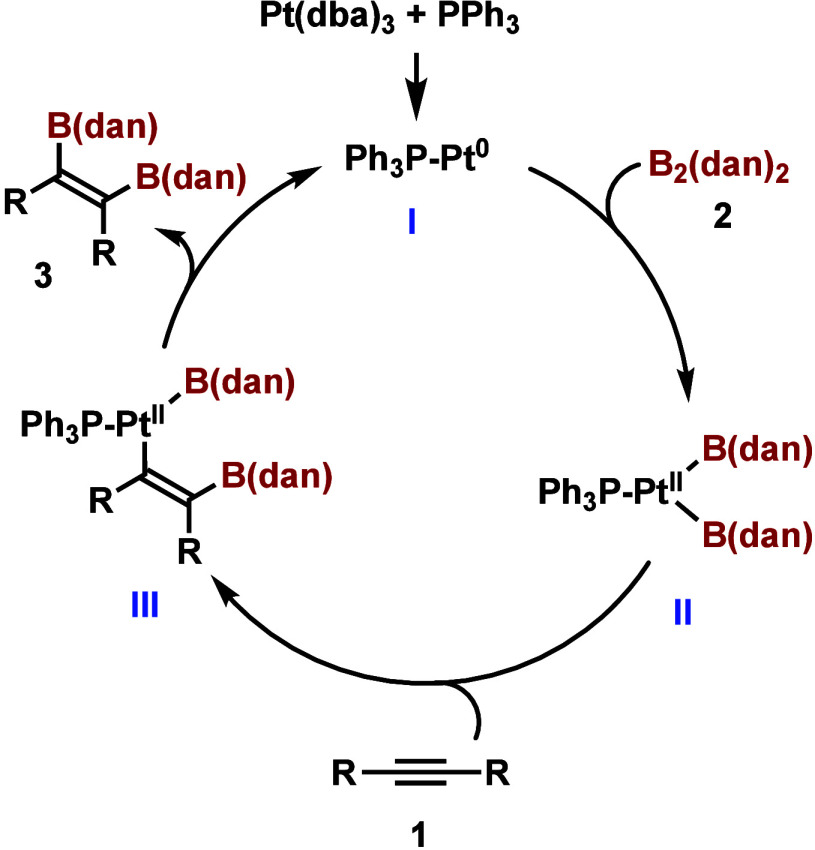
Proposed Mechanism for the Pt-Catalyzed Diboration of Alkynes with B_2_(dan)_2_

The diborylalkenes synthesized in this study are useful synthetic intermediates (Scheme 5). For example, the hydrogenation of **3a** proceeded in the presence of Pd/C and the corresponding diborylalkane **4** was synthesized in 88% yield.^14^ The treatment of **3a** with *t*-BuOOH (1.2 equiv) and KH (1.2 equiv) gave ketone **5**. The monomethylation of **3a** proceeded with moderate selectivity: the α-position was methylated preferentially^15^ and compound **5** was isolated in 57% yield. Though the monoarylation of **3a** did not proceed selectively, the Pd-catalyzed diarylation of **3a** with 4-iodotoluene (2.2 equiv) proceeded and triarylethene **7** was isolated in 52% yield.

**Scheme 5:**
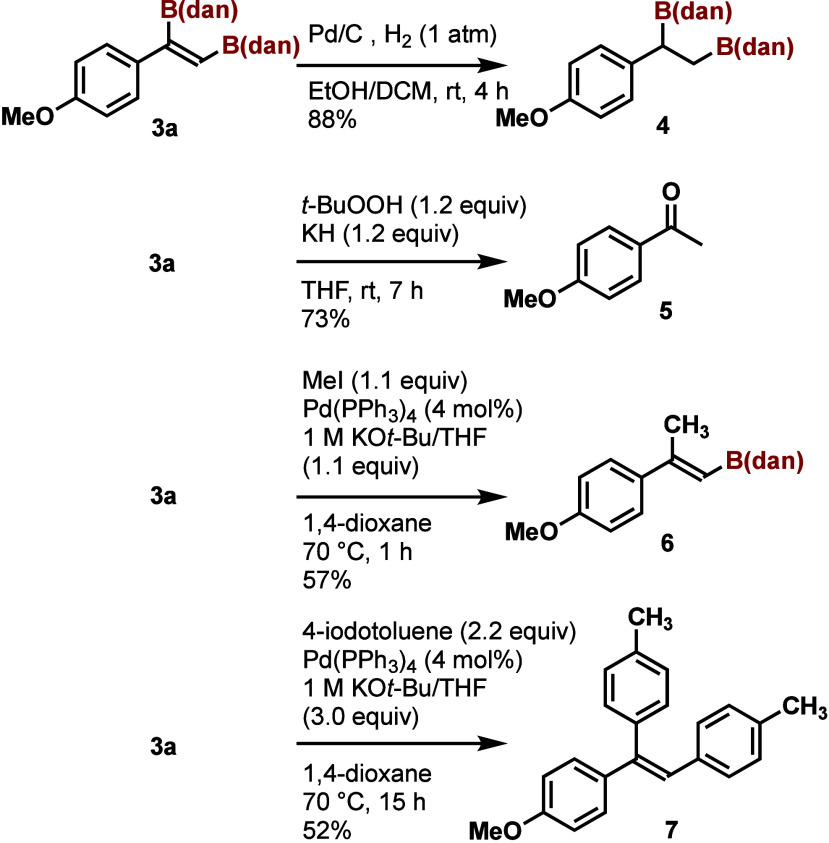
Derivatization of Diborylalkene **3a**

In summary, we succeeded in the synthesis of 1,8-diaminonaphthalene-protected 1,2-diborylalkenes by the Pt-catalyzed reaction of alkyne with B_2_(dan)_2_. A wide range of diborylalkenes could be synthesized by simple operation. The study would contribute to the expansion of the scope of the chemistry of organoboranes.

## Data Availability

The data underlying this study are available in the published article and its online Supporting Information.

## Supporting Information Available

Experimental procedures and characterization data (PDF)
