# Supplementary material for: Platinum-Catalyzed Diboration of Alkynes by 1,8-Diaminonaphthalene-Protected Diboronic Acid (B2(dan)2)
Source: J Org Chem. 2024 Oct 25;89(22):16947–51. doi: 10.1021/acs.joc.4c01939 (PMC11574848; doi:10.1021/acs.joc.4c01939)
Supplement: Supplementary file 1 — jo4c01939_si_001.pdf [file jo4c01939_si_001.pdf]

Supporting Information

Platinum-Catalyzed Diboration of Alkynes by 1,8-Diaminonaphthalene-Protected Diboronic Acid ($B_2(dan)_2$)

Shinichi Saito*, Yuya Koizumi, Yuki Ito, Taiga Yasuda, and Yusuke Yoshigoe

Department of Chemistry, Faculty of Science, Tokyo University of Science
Kagurazaka, Shinjuku, Tokyo 162-8601, Japan

*E-mail: ssaito@rs.tus.ac.jp

Table of Contents

General Information S2
Experimental Section S3
I. Optimization of the Reaction Conditions S3
II. General Procedure for Platinum-Catalyzed Diboration S4
III. Characterization Data S4
IV. Derivatization of Diborylalkene **3a** S13
References S15
Copy of NMR Spectra S16

General Information

Unless otherwise noted, all reactions were carried out in oven- or flame-dried glassware under an inert atmosphere of argon. Toluene and 1,4-dioxane of anhydrous grade were purchased from commercial sources and stored over activated molecular sieves (4A). $Pt_2(dba)_3$,[1] $Pt(dba)_2$,[2] **1p**,[3] **1q**,[4] and $B_2(dan)_2$ (**2**)[5] were synthesized by reported method. Unless otherwise noted, other reagents and solvents were commercially available and used without further purification. An oil bath or a bead bath was used as the heat source, and the external temperature was reported. NMR spectra were recorded on a 400 MHz spectrometer. Chemical shifts were reported in delta units (δ) relative to residual $CHCl_3$ in $CDCl_3$ (7.24 ppm) or DMSO in DMSO-d_6 (2.50 ppm) for ^1H NMR, $CHCl_3$ in $CDCl_3$ (77.23 ppm) or DMSO in DMSO-d_6 (39.52 ppm) for ^{13}C NMR. $^{11}B\{^1H\}$ NMR spectra were measured in quartz NMR tube, and the chemical shits were referenced to the $^{11}B\{^1H\}$ signal of $BF_3{\cdot}OEt_2$ (0.00 ppm) as an external standard. Multiplicity is indicated by s (singlet), d (doublet), t (triplet), m (multiplet) and br (broad). Coupling constants, *J*, are reported in Hertz. IR spectra were recorded on a Fourier transform infrared spectrometer using a diamond ATR module. Thin layer chromatography was performed on Merck silica gel 60F-254 plates. Column chromatography was performed using Kanto Chemical silica gel 60N (spherical, neutral 40–50 μm). A YMC-GPC T30000 (21.2 mm ID × 600 mm L) column was used for gel permeation chromatography (GPC) using $CHCl_3$ as the eluent. High-resolution mass spectra (HRMS) were obtained on a quadrupole time-of-flight (TOF) mass analyzer.

Experimental Section

I. Optimization of Reaction conditions

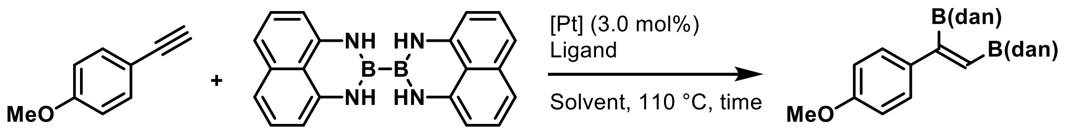

1a **2** (1.1 equiv) **3a**

A mixture of platinum catalyst (0.0030 mmol Pt, 3.0 mol %), ligand (0.0030 mmol, 3.0 mol %), $B_2(dan)_2$ (**2**, 37 mg, 0.11 mmol, 1.1 equiv) and **1a** (13 mg, 0.10 mmol, 1.0 equiv) in toluene (1.0 mL) was stirred at 110 °C. After the completion of the reaction, the resulting mixture was evaporated and the residue was purified by silica gel column chromatography (hexane/AcOEt = 10:1) to afford **3a**.

Table S1. Optimization of the reaction conditions

Entry	[Pt]	Ligand (x mol %)	Solvent	Time (h)	Yield (%)
1	$Pt(PPh_3)_4$	-	DMF	24	0
2	$Pt(PPh_3)_4$	-	toluene	23	78
3	$Pt_2(dba)_3$	PPh_3 (3.0 mol %)	toluene	0.5	77
4	$Pt(dba)_2$	PPh_3 (3.0 mol %)	toluene	0.5	81
5	$Pt(dba)_3$	PPh_3 (3.0 mol %)	toluene	0.5	84
6	$Pt(dba)_3$	PPh_3 (6.0 mol %)	toluene	3	84
7	$Pt(dba)_3$	PPh_3 (9.0 mol %)	toluene	4	79
8	$Pt(dba)_3$	-	toluene	0.5	(48) [a]
9	$Pt(dba)_3$	PPh_2(*o*-Tol) (3.0 mol %)	toluene	1	83
10	$Pt(dba)_3$	$P(4\text{-}CF_3C_6H_4)_3$ (3.0 mol %)	toluene	1	77
11	$Pt(dba)_3$	$P(C_6F_5)_3$ (3.0 mol %)	toluene	1	73
12	$Pt(dba)_3$	PCy_3 (3.0 mol %)	toluene	1	42
13	$Pt(dba)_3$	XPhos (3.0 mol %)	toluene	1	35

[a]The product was not isolated in pure form.

II. General Procedure for Platinum-Catalyzed Diboration

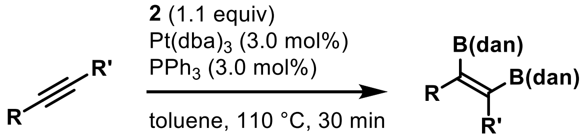

1 3

A mixture of $Pt(dba)_3$ (2.7 mg, 0.0030 mmol, 3.0 mol %), PPh_3 (0.79 mg, 0.0030 mmol, 3.0 mol %), $B_2(dan)_2$ (**2**, 37 mg, 0.11 mmol, 1.1 equiv) and alkyne **1** (0.10 mmol, 1.0 equiv) in toluene (1.0 mL) was stirred at 110 °C for 0.5 h. The resulting mixture was evaporated and the residue was purified by silica gel column chromatography (hexane/AcOEt) to afford **3.**

III. Characterization Data

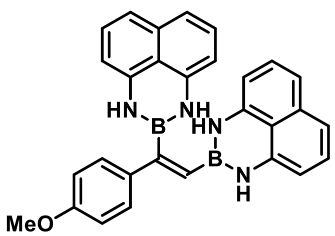

3a

(*E*)-2,2'-(1-(4-Methoxyphenyl)ethene-1,2-diyl)bis(2,3-dihydro-1H-naphtho[1,8-de][1,3,2]-diazaborinine) (**3a**): **3a** (34 mg, 0.084 mmol, 84%) was synthesized from 4-ethynylanisole (**1a**, 13 mg, 0.10 mmol). Purified by silica gel column chromatography (hexane/AcOEt = 10:1). White solid. Mp: 240.0–241.2 °C; ^{1}H NMR ($CDCl_3$, 400 MHz): δ 7.45 (d, 2H, J = 8.2 Hz), 7.14-7.08 (m, 4H), 6.99 (t, 2H, J = 7.5 Hz), 6.94 (d, 2H, J = 8.2 Hz), 6.88 (d, 2H, J = 8.7 Hz), 6.37 (s, 1H), 6.33 (d, 2H, J = 6.8 Hz), 6.11 (d, 2H, J = 6.8 Hz), 6.02 (s, 2H), 5.86 (s, 2H), 3.81 (s, 3H); ^{13}C{^{1}H} NMR ($CDCl_3$, 100 MHz): δ 159.9, 141.2, 140.4, 136.5, 136.4, 135.7, 128.4, 127.9, 127.8, 120.0, 119.9, 118.6, 117.7, 114.3, 106.6, 106.1, 55.6, the signals of the boron-bound carbon atom were obscure due to the quadrupolar boron nucleus; ^{11}B{^{1}H} NMR ($CDCl_3$, 128 MHz): δ 31.0; IR (ATR): 3401, 3053, 2951, 1606, 1507, 1412, 818 cm^{-1}; HRMS (ESI/Q-TOF) *m/z*: $[M+H]^+$ calcd. for $C_{29}H_{25}{}^{10}B_2N_4O$ 465.2282. Found 465.2282.

Large-scale synthesis of 3a (2.5 mmol scale): A mixture of $Pt(dba)_3$ (67 mg, 0.075 mmol, 3 mol %), PPh_3 (19.7 mg, 0.075 mmol, 3 mol%), $B_2(dan)_2$ (920 mg, 2.8 mmol, 1.1 equiv) and **1a** (330 mg, 2.5 mmol, 1.0 equiv) in toluene (25 ml) was stirred at 110 °C for 1 h. The resulting mixture was evaporated, and the residue was purified by recrystallization (hexane/AcOEt = 10:1) to afford **3a** (770 mg, 1.6 mmol, 66%).

Large-scale synthesis of 3a (5.0 mmol scale): A mixture of $Pt(dba)_3$ (140 mg, 0.15 mmol, 3 mol %), PPh_3 (39 mg, 0.15 mmol, 3 mol%), $B_2(dan)_2$ (1.8 g, 5.5 mmol, 1.1 equiv) and **1a** (660 mg, 5.0 mmol, 1.0 equiv) in

toluene (50 ml) was stirred at 110 °C for 1 h. The resulting mixture was evaporated, and the residue was purified by recrystallization (hexane/AcOEt = 10:1) to afford **3a**. The mother liquid was evaporated and the residue was purified by silica gel column chromatography (hexane/AcOEt = 10:1) to afford **3a**. Combined yield of **3a** was 1.8 g (3.8 mmol, 77%).

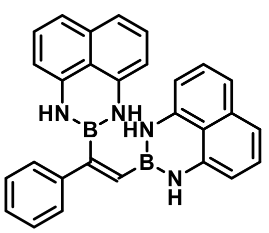

3b

(*E*)-2,2'-(1-Phenylethene-1,2-diyl)bis(2,3-dihydro-1*H*-naphtho[1,8-*de*][1,3,2]diazaborinine) (**3b**): **3b**[6] (40 mg, 0.093 mmol, 93%) was synthesized from phenylacetylene (**1b**, 14 mg, 0.10 mmol). Purified by silica gel column chromatography (hexane/AcOEt = 10:1). Yellow solid. Mp: 216.0-217.1 °C; ^{1}H NMR ($CDCl_3$, 400 MHz): δ 7.50-7.48 (m, 2H), 7.38-7.29 (m, 3H), 7.14-7.07 (m, 4H), 7.03-6.95 (m, 4H), 6.47 (s, 1H), 6.32 (dd, 2H, *J* = 6.6, 1.6 Hz), 6.14 (dd, 2H, *J* = 7.0, 1.0 Hz), 6.03 (s, 2H), 5.86 (s, 2H); ^{13}C{^{1}H} NMR ($CDCl_3$, 100 MHz): δ 143.4, 141.1, 140.3, 136.5, 136.4, 128.9, 128.2, 127.9, 127.8, 127.2, 120.0, 119.9, 118.6, 117.9, 106.6, 106.2, the signals of the boron-bound carbon atom were obscure due to the quadrupolar boron nucleus; ^{11}B{^{1}H} NMR ($CDCl_3$, 128 MHz): δ 28.5; IR (ATR): 3392, 3051 cm^{-1}; HRMS (ESI/Q-TOF) *m/z*: $[M + H]^+$ calcd for $C_{28}H_{23}{}^{10}B_2N_4$ 435.2176. Found 435.2174.

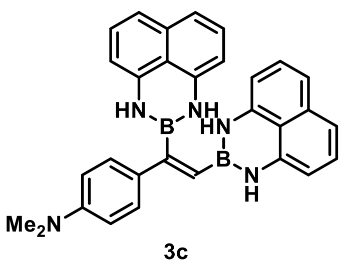

3c

(*E*)-4-(1,2-Bis(1*H*-naphtho[1,8-*de*][1,3,2]diazaborinin-2(3*H*)-yl)vinyl)-*N*,*N*-dimethylaniline (**3c**): **3c** (40 mg, 0.083 mmol, 83%) was synthesized from 4-ethynyl-*N,N*-dimethylaniline (**1c**, 14 mg, 0.10 mmol). Purified by silica gel column chromatography (hexane/AcOEt = 10:1). Yellow solid. Mp: 219.8 °C (decomp); ^{1}H NMR ($CDCl_3$, 400 MHz): δ 7.43 (d, 2H, *J* = 6.9 Hz), 7.14-7.07 (m, 4H), 6.98 (t, 2H, *J* = 8.0 Hz), 6.92 (dd, 2H, *J* = 8.3, 1.0 Hz), 6.68 (d, 2H, *J* = 8.8 Hz), 6.34-6.32 (m, 3H), 6.09 (dd, 2H, *J* = 7.1, 1.1 Hz), 6.03 (s, 2H), 5.88 (s, 2H), 2.97 (s, 6H); ^{13}C{^{1}H} NMR ($CDCl_3$, 100 MHz): δ 150.6, 141.4, 140.5, 136.6, 136.4, 130.8, 128.2, 127.9, 127.8, 120.0, 119.8, 118.5, 117.6, 112.4, 106.6, 106.0, 40.6, the signals of the boron-bound carbon atom were obscure due to the quadrupolar boron nucleus; ^{11}B{^{1}H} NMR ($CDCl_3$, 128 MHz): δ 29.1; IR (ATR): 3385, 3050, 2799, 2358, 2103, 1886 cm^{-1}; HRMS (ESI/Q-TOF) *m/z*: $[M + H]^+$ calcd for $C_{30}H_{28}{}^{10}B_2N_5$ 478.2598. Found 478.2599.

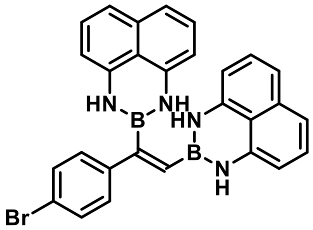

3d

(*E*)-2,2'-(1-(4-Bromophenyl)ethene-1,2-diyl)bis(2,3-dihydro-1*H*-naphtho[1,8-*de*][1,3,2]diazaborinine) (**3d**): **3d** (36 mg, 0.071 mmol, 71%) was synthesized from 1-bromo-4-ethynylbenzene (**1d**, 18 mg, 0.10 mmol). Purified by silica gel column chromatography (hexane/AcOEt = 10:1). Yellow solid. Mp: 260.3-261.1 °C; ^{1}H NMR ($CDCl_3$, 400 MHz): δ 7.47 (d, J = 8.4 Hz, 2H), 7.35 (d, J = 8.4 Hz, 2H), 7.14-7.07 (m, 4H), 7.03-6.95 (m, 4H), 6.45 (s, 1H), 6.33 (dd, J = 6.6, 1.8 Hz, 2H), 6.14 (dd, J = 7.2, 1.2 Hz, 2H), 6.02 (s, 2H), 5.84 (s, 2H); ^{13}C{^{1}H} NMR (DMSO-d_6, 100 MHz): δ 142.5, 141.72, 141.68, 135.9, 131.5, 128.5, 127.7, 120.6, 119.6, 119.3, 116.6, 116.3, 105.6, 105.2, the signals of the boron-bound carbon atom were obscure due to the quadrupolar boron nucleus and two signal were missing; ^{11}B{^{1}H} NMR ($CDCl_3$, 128 MHz): δ 29.8; IR (ATR): 3400, 3389, 3048 cm^{-1}; HRMS (ESI/Q-TOF) *m/z*: [M + H]$^{+}$ calcd for $C_{28}H_{22}{}^{10}B_2{}^{79}BrN_4$ 513.1281. Found 513.1280.

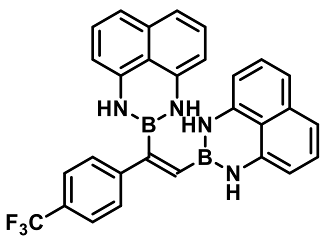

3e

(*E*)-2,2'-(1-(4-(Trifluoromethyl)phenyl)ethene-1,2-diyl)bis(2,3-dihydro-1*H*-naphtho[1,8-*de*][1,3,2]-diazaborinine) (**3e**): **3e** (37.1 mg, 0.074 mmol, 74%) was synthesized from 1-ethynyl-4-(trifluoromethyl)-benzene (**1e**, 17 mg, 0.10 mmol). Purified by silica gel column chromatography (hexane/AcOEt = 10:1). Yellow solid. Mp: 258.3-259.4 °C; ^{1}H NMR ($CDCl_3$, 400 MHz): δ 7.58 (dd, 4H, J = 15.6, 8.4 Hz), 7.14-7.08 (m, 4H), 7.04-6.96 (m, 4H), 6.52 (s, 1H), 6.34 (dd, 2H, J = 6.5, 2.0 Hz), 6.16 (dd, 2H, J = 7.0, 1.3 Hz), 6.03 (s, 2H), 5.84 (s, 2H); ^{13}C{^{1}H} NMR (DMSO-d_6, 100 MHz): δ 152.8, 147.5, 141.7, 141.6, 135.93, 135.91, 134.8, 128.0-127.7 (m), 127.1, 125.6-125.5 (m), 124.4 (q, $J_{F,C}$ = 270.9 Hz), 119.7, 119.4, 116.7, 116.4, 105.7, 105.2, the signals of the boron-bound carbon atom were obscure due to the quadrupolar boron nucleus; ^{11}B{^{1}H} NMR ($CDCl_3$, 128 MHz): δ 29.2, 27.3; IR (ATR): 3422, 3399, 3358, 2229 cm^{-1}, HRMS (ESI/Q-TOF) *m/z*: [M + H]$^{+}$ calcd for $C_{29}H_{22}{}^{10}B_2F_3N_4$ 503.2050. Found 503.2051.

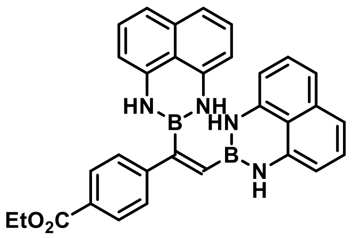

3f

Ethyl (*E*)-4-(1,2-bis(1*H*-naphtho[1,8-*de*][1,3,2]diazaborinin-2(3*H*)-yl)vinyl)benzoate (**3f**): **3f** (46 mg, 0.091 mmol, 91%) was synthesized from ethyl 4-ethynylbenzoate (**1f**, 17 mg, 0.10 mmol). Purified by silica gel column chromatography (hexane/AcOEt = 5:1). Yellow solid. Mp: 249.2-250.8 °C; ^{1}H NMR ($CDCl_3$, 400 MHz): δ 8.01 (d, 2H, *J* = 8.5 Hz), 7.53 (d, 2H, *J* = 8.3 Hz), 7.14-7.08 (m, 4H), 7.03-6.95 (m, 4H), 6.54 (s, 1H), 6.33 (dd, 2H, *J* = 6.6, 1.6 Hz), 6.15 (dd, 2H, *J* = 7.1, 1.1 Hz), 6.04 (s, 2H), 5.85 (s, 2H), 4.37 (q, 2H, *J* = 7.2 Hz), 1.38 (t, 3H, *J* = 7.1 Hz); ^{13}C{^{1}H} NMR ($CDCl_3$, 100 MHz): δ 166.6, 158.7, 147.9, 141.0, 140.2, 136.5, 136.4, 130.2, 130.0, 127.9, 127.8, 127.1, 120.0, 118.8, 118.0, 106.8, 106.3, 61.3, 14.6, the signals of the boron-bound carbon atom were obscure due to the quadrupolar boron nucleus; ^{11}B{^{1}H} NMR ($CDCl_3$, 128 MHz): δ 28.4; IR (ATR): 3400, 3379, 3055, 3002, 2978, 2903 cm^{-1}. HRMS (ESI/Q-TOF) *m/z*: $[M + H]^+$ calcd for $C_{31}H_{27}{}^{10}B_2N_4O_2$ 507.2387. Found 507.2386.

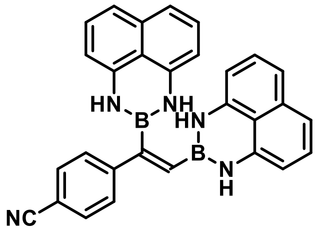

3g

(*E*)-4-(1,2-Bis(1*H*-naphtho[1,8-*de*][1,3,2]diazaborinin-2(3*H*)-yl)vinyl)benzonitrile (**3g**): **3g** (37 mg, 0.081 mmol, 81%) was synthesized from 4-ethynylbenzonitrile (**1g**, 13 mg, 0.10 mmol). Purified by silica gel column chromatography (hexane/AcOEt = 10:1). Yellow solid. Mp: 264.2 °C (decomp); ^{1}H NMR ($CDCl_3$, 400 MHz): δ 7.64 (d, 2H, *J* = 8.0 Hz), 7.56 (d, 2H, *J* = 8.3 Hz), 7.14-7.09 (m, 4H), 7.03-6.96 (m, 4H), 6.54 (s, 1H), 6.34 (dd, 2H, *J* = 6.3, 1.8 Hz), 6.15 (d, 2H, *J* = 7.0 Hz), 6.02 (s, 2H), 5.83 (s, 2H); ^{13}C NMR ($CDCl_3$, 100 MHz): δ 148.0, 140.8, 140.0, 136.5, 136.4, 132.7, 127.9, 127.82, 127.80, 120.0, 119.0, 118.2, 111.6, 106.9, 106.4, the signals of the boron-bound carbon atom were obscure due to the quadrupolar boron nucleus and some signals were missing; ^{11}B{^{1}H} NMR ($CDCl_3$, 128 MHz): δ 27.8; IR (ATR) 3422, 3399, 3358, 2229 cm^{-1}; HRMS (ESI/Q-TOF) *m/z*: $[M + H]^+$ calcd for $C_{29}H_{22}{}^{10}B_2N_5$; 460.2129. Found 460.2126.

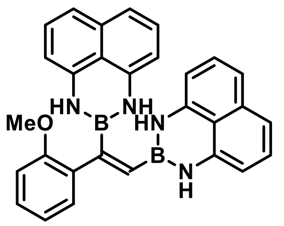

3h

(*E*)-2,2'-(1-(2-Methoxyphenyl)ethene-1,2-diyl)bis(2,3-dihydro-1*H*-naphtho[1,8-*de*][1,3,2]diazaborinine) (**3h**): **3h** (42 mg, 0.090 mmol, 90%) was synthesized from 2-ethynylanisole (**1h**, 13 mg, 0.10 mmol). Purified by silica gel column chromatography (hexane/AcOEt = 10:1). Yellow solid. Mp: 245.9-246.6 °C; ^{1}H NMR ($CDCl_3$, 400 MHz): δ 7.36-7.28 (m, 2H), 7.09 (t, 2H, *J* = 7.8 Hz), 7.05-7.01 (m, 5H), 6.95 (dd, 2H, *J* = 8.2, 1.0 Hz), 6.87 (d, 1H, *J* = 7.6 Hz), 6.28 (dd, 3H, *J* = 7.1, 1.1 Hz), 6.15 (dd, 2H, *J* = 7.1, 1.1 Hz), 6.08 (s, 2H), 5.78 (s, 2H), 3.73 (s, 3H); ^{13}C{^{1}H} NMR ($CDCl_3$, 100 MHz): δ 156.7, 141.2, 141.0, 136.6, 136.4, 134.8, 129.5, 128.7, 127.9, 127.8, 121.6, 120.0, 119.8, 118.0, 117.8, 111.3, 106.2, 106.1, 56.1, the signals of the boron-bound carbon atom were obscure due to the quadrupolar boron nucleus; ^{11}B{^{1}H} NMR ($CDCl_3$, 128 MHz): δ 28.2; IR (ATR): 3400, 3384, 3049, 2360 cm^{-1}; HRMS (ESI/Q-TOF) *m/z*: $[M + H]^+$ calcd for $C_{29}H_{25}{}^{10}B_2N_4O$ 465.2282. Found 465.2276.

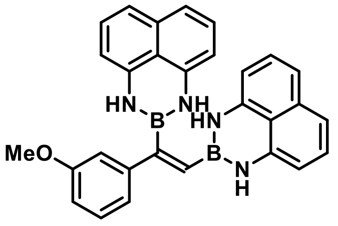

3i

(*E*)-2,2'-(1-(3-Methoxyphenyl)ethene-1,2-diyl)bis(2,3-dihydro-1*H*-naphtho[1,8-*de*][1,3,2]diazaborinine) (**3i**): **3i** (35 mg, 0.074 mmol, 74%) was synthesized from 3-ethynylanisole (**1i**, 13 mg, 0.10 mmol). Purified by silica gel column chromatography (hexane/AcOEt = 10:1). Yellow solid. Mp: 212.2-213.0 °C; ^{1}H NMR ($CDCl_3$, 400 MHz): δ 7.27 (t, 1H, *J* = 8.0 Hz), 7.13-7.05 (m, 5H), 7.02-6.94 (m, 5H), 6.85 (ddd, 1H, *J* = 8.2, 2.7, 0.8 Hz), 6.46 (s, 1H), 6.32 (dd, 2H, *J* = 6.8, 1.5 Hz), 6.14 (dd, 2H, *J* = 7.1, 1.1 Hz), 6.03 (s, 2H), 5.86 (s, 2H), 3.82 (s, 3H); ^{13}C{^{1}H} NMR ($CDCl_3$, 100 MHz): δ 160.1, 158.7, 145.0, 141.1, 140.4, 136.5, 136.4, 129.8, 127.9, 127.8, 120.02, 119.95, 118.6, 117.9, 113.5, 112.6, 106.6, 106.2, 55.5, the signals of the boron-bound carbon atom were obscure due to the quadrupolar boron nucleus; ^{11}B{^{1}H} NMR ($CDCl_3$, 128 MHz): δ 28.4; IR (ATR): 3416, 3394, 3048, 2956, 2933, 2829, 2360, 2335 cm^{-1}; HRMS (ESI/Q-TOF) *m/z*: $[M + H]^+$ calcd for $C_{29}H_{25}{}^{10}B_2N_4O$ 465.2282. Found 465.2280.

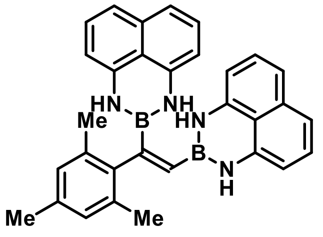

3j

(*E*)-2,2'-(1-Mesitylethene-1,2-diyl)bis(2,3-dihydro-1*H*-naphtho[1,8-*de*][1,3,2]diazaborinine) (3j): **3j** (40 mg, 0.083 mmol, 83%) was synthesized from 2-ethynyl-1,3,5-trimethylbenzene (**1j**, 14 mg, 0.100 mmol). Purified by silica gel column chromatography (hexane/AcOEt = 10:1). Yellow solid. Mp: 118.6-119.9 °C; ^{1}H NMR ($CDCl_3$, 400 MHz): δ 7.11 (t, 2H, *J* = 7.6 Hz), 7.07-6.98 (m, 6H), 6.92 (s, 2H), 6.33 (dd, 2H, *J* = 7.1, 1.1 Hz), 6.17 (dd, 2H, *J* = 6.9, 1.4 Hz), 6.13 (s, 1H), 5.93 (s, 2H), 5.80 (s, 2H), 2.32 (s, 3H), 2.21 (s, 6H); ^{13}C{^{1}H} NMR ($CDCl_3$, 100 MHz): δ 142.3, 140.8, 140.7, 136.5, 136.4, 136.0, 134.5, 128.8, 127.9, 127.8, 120.1, 120.0, 118.3, 118.1, 106.42, 106.38, 21.2, 21.0, the signals of the boron-bound carbon atom were obscure due to the quadrupolar boron nucleus; ^{11}B{^{1}H} NMR ($CDCl_3$, 128 MHz): δ 28.3; IR (ATR): 3401, 3057, 2913, 2849 cm^{-1}; HRMS (ESI/Q-TOF) *m/z*: $[M + H]^+$ calcd for $C_{31}H_{29}{}^{10}B_2N_4$ 477.2646. Found 477.2647.

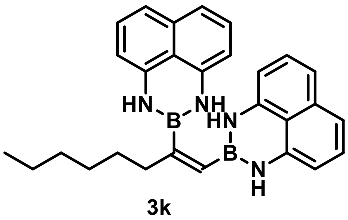

3k

(*E*)-2,2'-(Oct-1-ene-1,2-diyl)bis(2,3-dihydro-1*H*-naphtho[1,8-*de*][1,3,2]diazaborinine) (3k): **3k** (32 mg, 0.071 mmol, 71%) was synthesized from 1-octyne (**1k**, 11 mg, 0.10 mmol). Purified by silica gel column chromatography (hexane/AcOEt = 20:1). Orange solid. Mp: 94.4-95.0 °C; ^{1}H NMR ($CDCl_3$, 400 MHz): δ 7.13-7.05 (m, 4H), 7.02-6.93 (m, 4H), 6.33 (dd, 2H, *J* = 7.1, 1.1 Hz), 6.13 (dd, 2H, *J* = 7.3, 1.3 Hz), 5.92 (s, 1H), 5.90 (s, 2H), 5.74 (s, 2H), 2.31 (td, 2H, *J* = 7.8, 1.2 Hz), 1.46 (q, 2H, *J* = 7.6 Hz), 1.34-1.26 (m, 6H), 0.88 (t, 3H, *J* = 6.9 Hz); ^{13}C{^{1}H} NMR ($CDCl_3$, 100 MHz): δ 141.2, 140.5, 136.45, 136.39, 127.84, 127.75, 119.9, 119.8, 118.3, 117.6, 106.4, 106.0, 41.7, 31.9, 29.5, 22.8, 14.3, the signals of the boron-bound carbon atom were obscure due to the quadrupolar boron nucleus and one signal was missing; ^{11}B{^{1}H} NMR ($CDCl_3$, 128 MHz): δ 28.4; IR (ATR): 3405, 3050 cm^{-1}; HRMS (ESI/Q-TOF) *m/z*: $[M + H]^+$ calcd for $C_{28}H_{31}{}^{10}B_2N_4$ 443.2802. Found 443.2800.

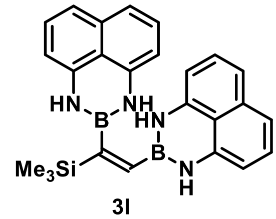

3l

(*E*)-2,2'-(1-(Trimethylsilyl)ethene-1,2-diyl)bis(2,3-dihydro-1*H*-naphtho[1,8-*de*][1,3,2]diazaborinine) (**3l**): **3l** (27 mg, 0.063 mmol, 63%) was synthesized from trimethylsilylacetylene (**1l**, 9.8 mg, 0.10 mmol). The mixture was stirred at 80 °C for 1 h. Purified by silica gel column chromatography (hexane/AcOEt = 10:1). White solid. Mp: 118.1-118.9 °C; ^{1}H NMR (CDCl$_3$, 400 MHz): δ 7.16-7.07 (m, 4H), 7.01-6.93 (m, 4H), 6.67 (s, 1H), 6.36 (dd, 2H, *J* = 7.0, 1.3 Hz), 6.10 (dd, 2H, *J* = 7.1, 1.1 Hz), 5.99 (s, 2H), 5.56 (s, 2H), 0.18 (s, 9H); ^{13}C{^{1}H} NMR (CDCl$_3$, 100 MHz): δ 141.2, 140.5, 136.5, 136.4, 127.8, 127.7, 120.0, 119.6, 118.3, 117.7, 106.4, 106.1, -0.9, the signals of the boron-bound carbon atom were obscure due to the quadrupolar boron nucleus; ^{11}B{^{1}H} NMR (CDCl$_3$, 128 MHz): δ 31.0, 26.9; IR (ATR): 3428, 3401, 3387, 3318, 3052, 2952, 2869 cm^{-1}; HRMS (ESI/Q-TOF) *m/z*: [M + H]$^+$ calcd for $C_{25}H_{27}{}^{10}B_2N_4Si$ 431.2258. Found 431.2258.

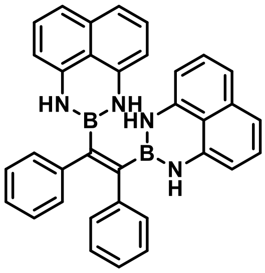

3m

(*Z*)-1,2-Bis(1*H*-naphtho[1,8-*de*][1,3,2]diazaborinin-2(3*H*)-yl)-1,2-diphenylethene (**3m**): **3m** (46 mg, 0.090 mmol, 90%) was synthesized from diphenylacetylene (**1m**, 18 mg, 0.10 mmol). The mixture was stirred at for 1 h. Purified by silica gel column chromatography (hexane/AcOEt = 10:1). Yellow solid. Mp: 195.2-195.9 °C; ^{1}H NMR (CDCl$_3$, 400 MHz): δ 7.16-7.07 (m, 6H), 7.03-6.97 (m, 12H), 6.18 (dd, 4H, *J* = 6.8, 1.5 Hz), 5.83 (s, 4H); ^{13}C{^{1}H} NMR (CDCl$_3$, 100 MHz): δ 142.3, 140.8, 136.4, 129.8, 128.3, 127.8, 126.6, 119.9, 118.1, 106.4, the signals of the boron-bound carbon atom were obscure due to the quadrupolar boron nucleus; ^{11}B{^{1}H} NMR (CDCl$_3$, 128 MHz): δ 29.6; IR (ATR) 3405, 3050 cm^{-1}; HRMS (ESI/Q-TOF) *m/z*: [M + H]$^+$ calcd for $C_{34}H_{27}{}^{10}B_2N_4$ 511.2489. Found 511.2487.

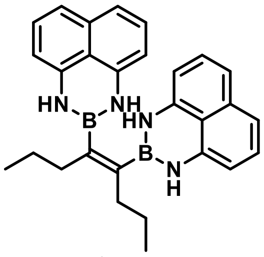

3n

(*Z*)-2,2'-(Oct-4-ene-4,5-diyl)bis(2,3-dihydro-1*H*-naphtho[1,8-*de*][1,3,2]diazaborinine) (**3n**): **3n** (29 mg, 0.065 mmol, 65%) was synthesized from 4-octyne (**1n**, 18 mg, 0.10 mmol). Purified by silica gel column chromatography (hexane/AcOEt = 20:1). White solid. Mp: 195.2-195.9 °C; ^{1}H NMR ($CDCl_3$, 400 MHz): δ 7.04-6.94 (m, 8H), 6.20 (dd, 4H, *J* = 7.1, 1.1 Hz), 5.75 (s, 4H), 2.28-2.24 (m, 4H), 1.43 (sextet, 4H, *J* = 7.5 Hz), 0.96 (t, 6H, *J* = 7.3 Hz); ^{13}C{^{1}H} NMR ($CDCl_3$, 100 MHz): δ 141.0, 136.4, 127.7, 119.8, 117.8, 106.1, 33.8, 23.6, 14.7, the signals of the boron-bound carbon atom were obscure due to the quadrupolar boron nucleus; ^{11}B{^{1}H} NMR ($CDCl_3$, 128 MHz): δ 29.6; IR (ATR): 3403, 3393, 3048, 3006, 2952, 2924, 2866 cm^{-1}; HRMS (ESI/Q-TOF) *m/z*: [M + H]$^+$ calcd for $C_{28}H_{31}{}^{10}B_2N_4$ 443.2802. Found 443.2802.

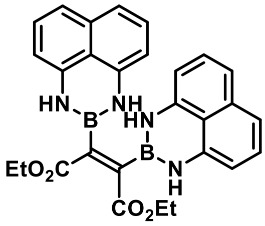

3o

Diethyl 2,3-bis(1*H*-naphtho[1,8-*de*][1,3,2]diazaborinin-2(3*H*)-yl)maleate (**3o**): **3o** (18 mg, 0.036 mmol, 36%) was synthesized from diethyl acetylenedicarboxylate (**1o**, 17 mg, 0.10 mmol). Purified by silica gel column chromatography (hexane/AcOEt = 2:1). Orange solid. Mp: 206.1-207.3 °C; ^{1}H NMR ($CDCl_3$, 400 MHz): δ 7.05-6.99 (m, 8H), 6.24 (dd, 4H, *J* = 6.6, 1.6 Hz), 6.12 (s, 4H), 4.26 (q, 4H, *J* = 7.2 Hz), 1.31 (t, 6H, *J* = 7.1 Hz); ^{13}C{^{1}H} NMR ($CDCl_3$, 100 MHz): δ 169.7, 140.0, 136.4, 127.8, 120.2, 118.7, 106.8, 61.8, 14.3, the signals of the boron-bound carbon atom were obscure due to the quadrupolar boron nucleus; ^{11}B{^{1}H} NMR ($CDCl_3$, 128 MHz): δ 27.6; IR (ATR): 3372, 3057, 2977, 2360 cm^{-1}; HRMS (ESI/Q-TOF) *m/z*: [M + H]$^+$ calcd for $C_{28}H_{27}{}^{10}B_2N_4O_4$ 503.2286. Found 503.2287

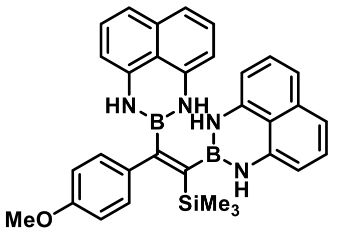

3p

(*Z*)-2,2'-(1-(4-Methoxyphenyl)-2-(trimethylsilyl)ethene-1,2-diyl)bis(2,3-dihydro-1*H*-naphtho[1,8-*de*]-[1,3,2]diazaborinine) (3p): **3p** (46 mg, 0.084 mmol, 84%) was synthesized from **1p** (20 mg, 0.10 mmol). Purified by silica gel column chromatography (hexane/AcOEt = 8:1). White solid. Mp: 237.2-237.8 °C; ^{1}H NMR ($CDCl_3$, 400 MHz): δ 7.15-7.06 (m, 4H), 7.01 (d, 2H, J = 8.4 Hz), 6.92-6.85 (m, 6H), 6.39 (dd, 2H, J = 7.1, 0.9 Hz), 5.93-5.88 (m, 4H), 5.68 (s, 2H), 3.85 (s, 3H), -0.12 (s, 9H); ^{13}C{^{1}H} NMR ($CDCl_3$, 100 MHz): δ 158.7, 141.2, 140.4, 138.4, 136.5, 136.3, 129.1, 127.8, 127.7, 119.8, 119.5, 118.4, 117.6, 113.9, 106.4, 106.1, 55.5, 1.3, the signals of the boron-bound carbon atom were obscure due to the quadrupolar boron nucleus; ^{11}B{^{1}H} NMR ($CDCl_3$, 128 MHz): δ 27.2; IR (ATR): 3410, 3359, 3044, 2952, 2892, 2842, 2361 cm^{-1}; HRMS (ESI/Q-TOF) *m/z*: $[M + H]^+$ calcd for $C_{32}H_{33}{}^{10}B_2N_4OSi$ 537.2677. Found 537.2680.

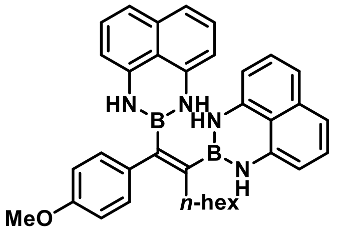

3q

(*Z*)-2,2'-(1-(4-methoxyphenyl)oct-1-ene-1,2-diyl)bis(2,3-dihydro-1*H*-naphtho[1,8-de][1,3,2]-diazaborinine) (3q): **3q** (52 mg, 0.094 mmol, 94%) was synthesized from **1q** (22 mg, 0.10 mmol). Purified by silica gel column chromatography (hexane/AcOEt = 10:1). Yellow oil. ^{1}H NMR ($CDCl_3$, 400 MHz): δ 7.14-6.99 (m, 6H), 6.97-6.89 (m, 6H), 6.34 (d, 2H, J = 7.2 Hz), 6.02 (d, 2H, J = 7.2 Hz), 5.84 (s, 2H), 5.76 (s, 2H), 3.85 (s, 3H), 2.14 (t, 2H, J = 7.8 Hz), 1.47-1.36 (m, 2H), 1.28-1.11 (m, 6H), 0.87-0.79 (m, 3H); ^{13}C{^{1}H} NMR ($CDCl_3$, 100 MHz): δ 158.2, 141.2, 140.6, 136.5, 136.3, 135.0, 129.5, 127.8, 127.7, 119.9, 119.6, 118.3, 117.6, 114.1, 106.4, 106.1, 55.5, 34.5, 31.7, 30.1, 29.8, 22.8, 14.2, the signals of the boron-bound carbon atom were obscure due to the quadrupolar boron nucleus; ^{11}B{^{1}H} NMR ($CDCl_3$, 128 MHz): δ 30.6; IR(ATR): 3412, 3051, 2953, 2927, 2853 cm^{-1}; HRMS (ESI/Q-TOF) *m/z*: $[M + H]^+$ calcd for $C_{35}H_{37}{}^{10}B_2N_4O$ 549.3221. Found 549.3220.

IV. Derivatization of Diborylalkene 3a.

Procedure for the hydrogenation of 3a.

20 wt% Pd/C (14 mg) was added to a solution of **3a** (70 mg, 0.15 mmol) in DCM (1.5 mL) and EtOH (1.5 mL). The mixture was stirred under hydrogen (1 atm) for 4 h. After the completion of the reaction, the resulting mixture was filtered through Celite and the filtrate was concentrated in vacuo. The residue was purified by silica gel column chromatography (hexane/AcOEt = 10:1) to afford **4** (62 mg, 0.13 mmol, 88%).

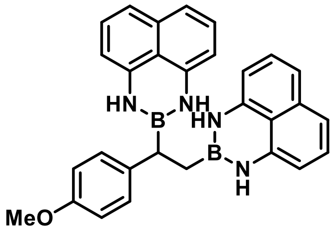

4

2,2'-(1-(4-Methoxyphenyl)ethane-1,2-diyl)bis(2,3-dihydro-1H-naphtho[1,8-de][1,3,2] diazaborinine) (**4**): White amorphous solid. Mp: 109.4-110.8 °C; ^{1}H NMR ($CDCl_3$, 400 MHz): δ 7.12 (d, 2H, J = 8.8 Hz), 7.09-7.04 (m, 4H), 6.99 (t, 4H, J = 8.0 Hz), 6.88 (d, 2H, J = 8.8 Hz), 6.23 (d, 2H, J = 7.2 Hz), 6.16 (d, 2H, J = 7.2 Hz), 5.48 (s, 2H), 5.46 (s, 2H), 3.80 (s, 3H), 2.45 (dd, 1H, J = 9.6, 6.4 Hz), 1.29 (m, 2H); ^{13}C{^{1}H} NMR ($CDCl_3$, 100 MHz): δ 157.9, 141.1, 137.0, 136.45, 136.43, 128.8, 127.7, 119.7, 119.6, 117.9, 117.7, 114.6, 106.1, 105.8, 55.5, 29.3, 17.5, two signals were missing; ^{11}B{^{1}H} NMR ($CDCl_3$, 128 MHz): δ 33.5; IR (ATR): 3415, 3051, 2833 cm^{-1}; HRMS (ESI/Q-TOF) *m/z*: $[M + H]^+$ calcd for $C_{29}H_{27}{}^{10}B_2N_4O$ 467.2438. Found 467.2444.

Procedure for the oxidation of 3a.

To a solution of KH (30 wt% dispersion in mineral oil, 48 mg, 0.36 mmol, 1.2 equiv) and 5.5 M *t*-BuOOH/decane (66 μL, 0.36 mmol, 1.2 equiv) in THF (0.6 mL) was added **3a** (140 mg, 0.30 mmol, 1.0 equiv). The reaction mixture was stirred at room temperature for 7 h. To the resulting mixture was added sat. aq. $Na_2S_2O_4$ and the mixture was extracted with AcOEt (3 × 10 mL). The combined organic layer was washed with water and brine, dried over Na_2SO_4 and concentrated in vacuo. The residue was purified by silica gel column chromatography (hexane/AcOEt = 40:1) to afford **5** (33 mg, 0.22 mmol, 73%) as a colorless oil. The NMR data matched those reported previously.[7]

Procedure for the synthesis of 6 from 3a.

A mixture of **3a** (47 mg, 0.10 mmol, 1.0 equiv), $Pd(PPh_3)_4$ (4.6 mg, 4.0 μmol, 4 mol %), MeI (7.1 μL, 0.11 mmol, 1.1 equiv) and 1M KO*t*-Bu/THF (0.11 mL, 0.11 mmol, 1.1 equiv) in 1,4-dioxane (2 mL) was stirred at

70 °C for 1 h. To the mixture was added brine and the mixture was extracted with AcOEt (3 × 5 mL). The combined organic layer was dried over Na_2SO_4 and concentrated in vacuo. The residue was purified by silica gel column chromatography (hexane/AcOEt = 20:1) to afford **6** (18 mg, 0.057 mmol, 57%).

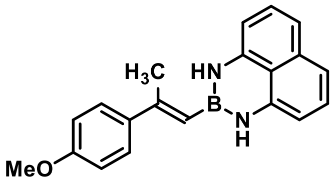

6

(*E*)-2-(2-(4-Methoxyphenyl)prop-1-en-1-yl)-2,3-dihydro-1H-naphtho[1,8-de][1,3,2] diazaborinine (**6**): White solid. Mp: 149.4-150.6 °C; ^{1}H NMR ($CDCl_3$, 400 MHz): δ 7.45 (d, 2H, *J* = 8.4 Hz), 7.11 (dd, 2H, *J* = 8.2, 7.2 Hz), 7.02 (dd, 2H, *J* = 8.2, 0.8 Hz), 6.84 (d, 2H, *J* = 8.4 Hz), 6.32 (dd, 2H, *J* = 7.2, 0.8 Hz), 5.83 (d, 1H, *J* = 0.8 Hz), 5.72 (s, 2H), 3.82 (s, 3H), 2.31 (d, 3H, *J* = 0.8 Hz); ^{13}C{^{1}H} NMR ($CDCl_3$, 100 MHz): δ 159.6, 150.2, 141.4, 136.6, 136.4, 127.8, 127.0, 119.8, 117.7, 113.8, 105.9, 55.5, 20.8, the signal of the boron-bound carbon atom was obscure due to the quadrupolar boron nucleus; ^{11}B{^{1}H} NMR ($CDCl_3$, 128 MHz): δ 26.3; IR (ATR): 3400, 3049, 2997, 2972, 2942 cm^{-1}; HRMS (ESI/Q-TOF) *m/z*: $[M + H]^+$ calcd for $C_{20}H_{20}{}^{10}BN_2O$ 314.1700. Found 314.1700.

Procedure for Suzuki-Miyaura cross-coupling of 3a.

A mixture of **3a** (47 mg, 0.10 mmol, 1.0 equiv), $Pd(PPh_3)_4$ (4.6 mg, 4.0 μmol, 4 mol %), 4-iodotoluene (48 mg, 0.22 mmol, 2.2 equiv) and 1M KO*t*-Bu/THF (0.30 mL, 0.30 mmol, 3.0 equiv) in 1,4-dioxane (2 mL) was stirred at 70 °C for 15 h. To the mixture was added brine and the mixture was extracted with AcOEt (3 × 5 mL). The combined organic layer was dried over Na_2SO_4 and concentrated in vacuo. The residue was purified by silica gel column chromatography (hexane/AcOEt = 40:1) and GPC to afford **7**[8] (16 mg, 0.052 mmol, 52%) as a white solid.

References

(1) Lewis, L. N.; Krafft, T. A.; Huffman, J. C. Crystal and molecular structure of a (dibenzylideneacetone)platinum dimer with partial Pt occupancy. *Inorg. Chem.* **1992**, *31* (17), 3555-3557. DOI: 10.1021/ic00043a014.

(2) Moseley, K.; Maitlis, P. M. Acetylenes and noble metal compounds. Part XI. Reactions of di-methyl acetylenedicarboxylate with dibenzylideneacetone–palladium and –platinum complexes: pallada- and platina-cyclopentadienes. *J. Chem. Soc., Dalton Trans.* **1974**, 169-175. DOI: 10.1039/DT9740000169.

(3) Gauthier, R.; Tzouras, N. V.; Zhang, Z.; Bédard, S.; Saab, M.; Falivene, L.; Van Hecke, K.; Cavallo, L.; Nolan, S. P.; Paquin, J.-F. Gold N-Heterocyclic Carbene Catalysts for the Hydrofluorination of Alkynes Using Hydrofluoric Acid: Reaction Scope, Mechanistic Studies and the Tracking of Elusive Intermediates. *Chem. Eur. J.* **2022**, *28* (4), e202103886. DOI: 10.1002/chem.202103886.

(4) Rasolofonjatovo, E.; Tréguier, B.; Provot, O.; Hamze, A.; Brion, J.-D.; Alami, M. A One-Pot Three-Step Synthesis of *Z*-Trisubstituted Olefins from Arylalkynes and Their Cyclization into 4-Aryl-2H-chromenes. *Eur. J. Org. Chem.* **2012**, *2012* (8), 1603-1615. DOI: 10.1002/ejoc.201101735.

(5) Yasuda, T.; Yoshigoe, Y.; Saito, S. Copper-Catalyzed Borylation of Styrenes by 1,8-Diaminonaphthalene-Protected Diboronic Acid. *Org. Lett.* **2023**, *25* (12), 2093-2097. DOI: 10.1021/acs.orglett.3c00451.

(6) Viereck, P.; Krautwald, S.; Pabst, T. P.; Chirik, P. J. A Boron Activating Effect Enables Cobalt-Catalyzed Asymmetric Hydrogenation of Sterically Hindered Alkenes. *J. Am. Chem. Soc.* **2020**, *142* (8), 3923-3930. DOI: 10.1021/jacs.9b12214.

(7) Cacchi, S.; Fabrizi, G.; Gavazza, F.; Goggiamani, A. Palladium-Catalyzed Reaction of Aryl Iodides with Acetic Anhydride. A Carbon Monoxide-Free Synthesis of Acetophenones. *Org. Lett.* **2003**, *5* (3), 289-291. DOI: 10.1021/ol027243b.

(8) Zhang, W.; Liu, M.; Wu, H.; Ding, J.; Cheng, J. Phosphine-free rhodium-catalyzed hydroarylation of diaryl acetylenes with boronic acids. *Tetrahedron Lett.* **2008**, *49* (35), 5214-5216. DOI: 10.1016/j.tetlet.2008.05.140.

NMR spectra

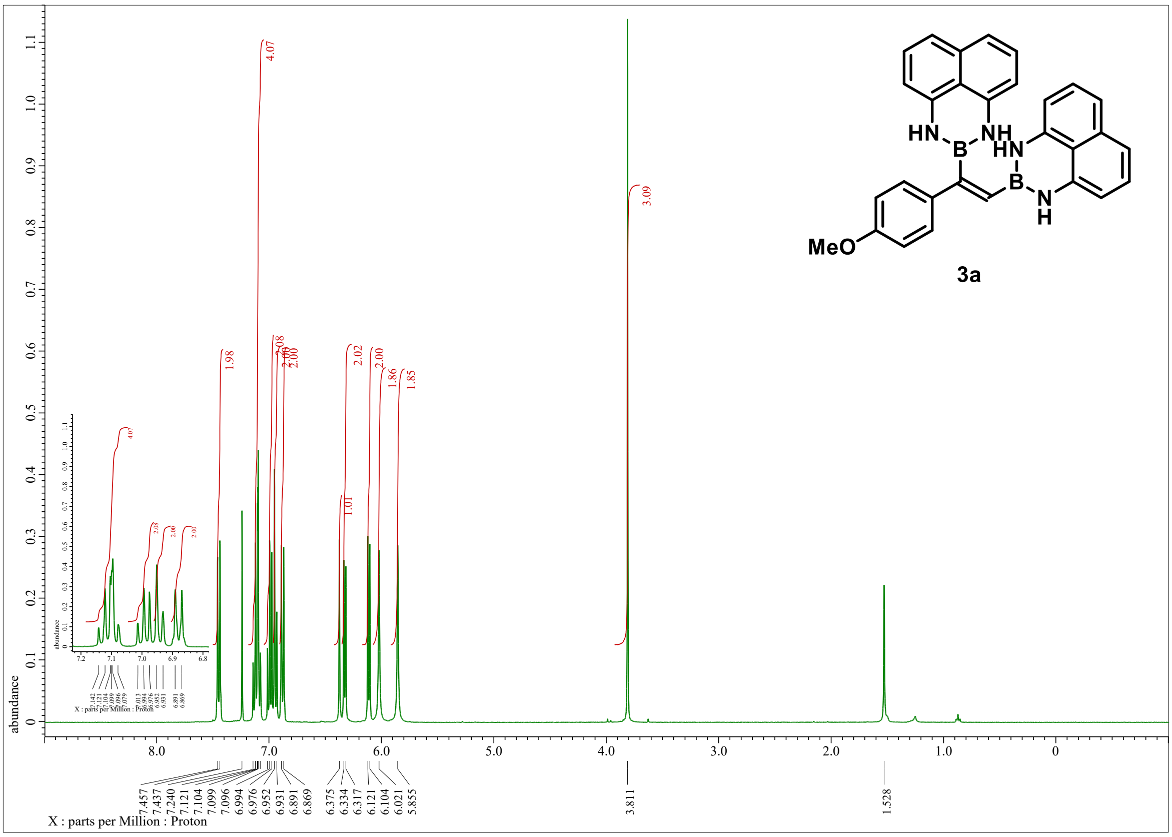

Figure S1. ^{1}H NMR ($CDCl_3$, 400 MHz) spectrum of **3a**.

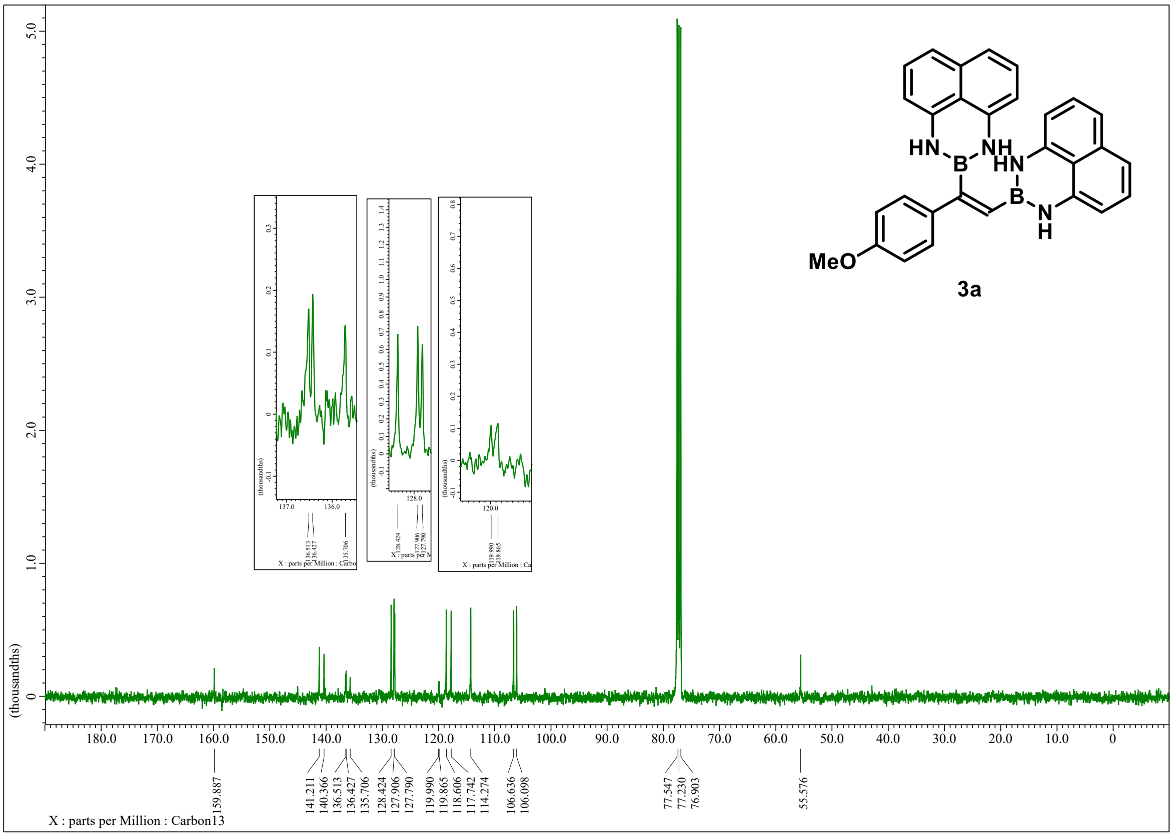

Figure S2. $^{13}C\{^1H\}$ NMR ($CDCl_3$, 100 MHz) spectrum of **3a**.

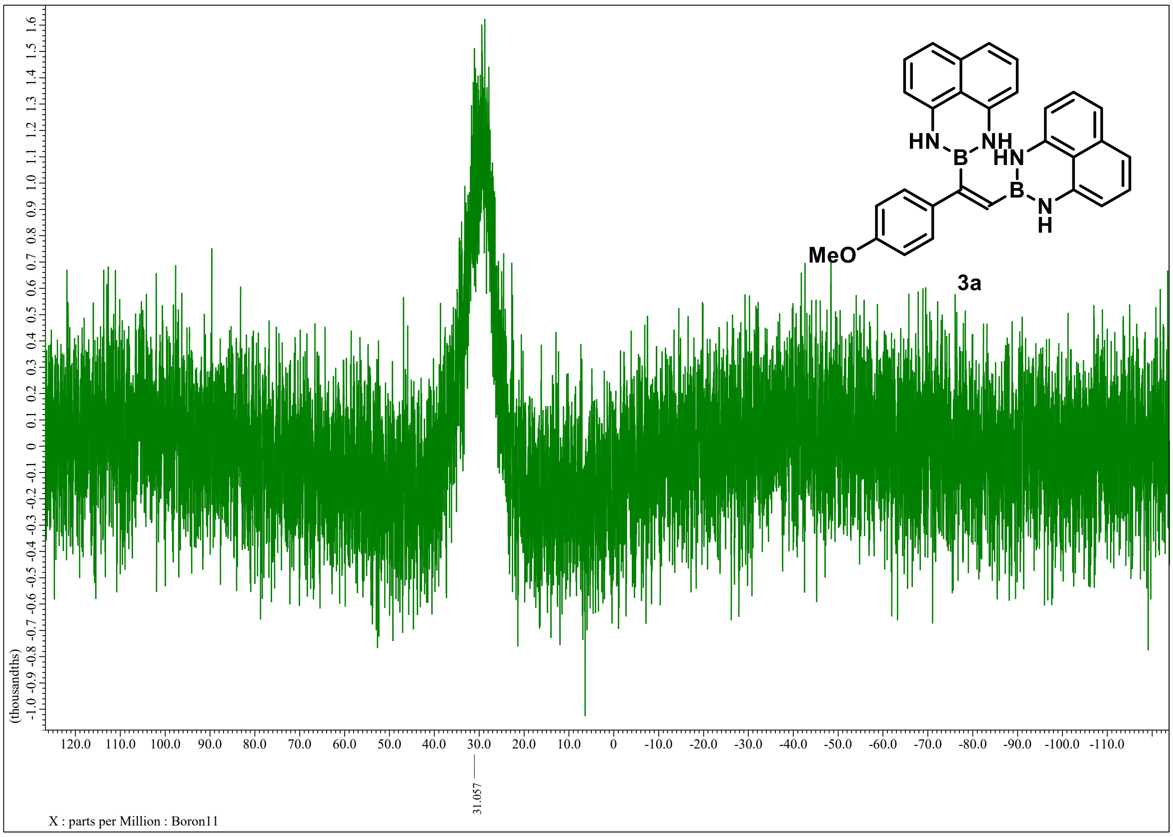

Figure S3. $^{11}B\{^1H\}$ NMR ($CDCl_3$, 128 MHz) spectrum of **3a**.

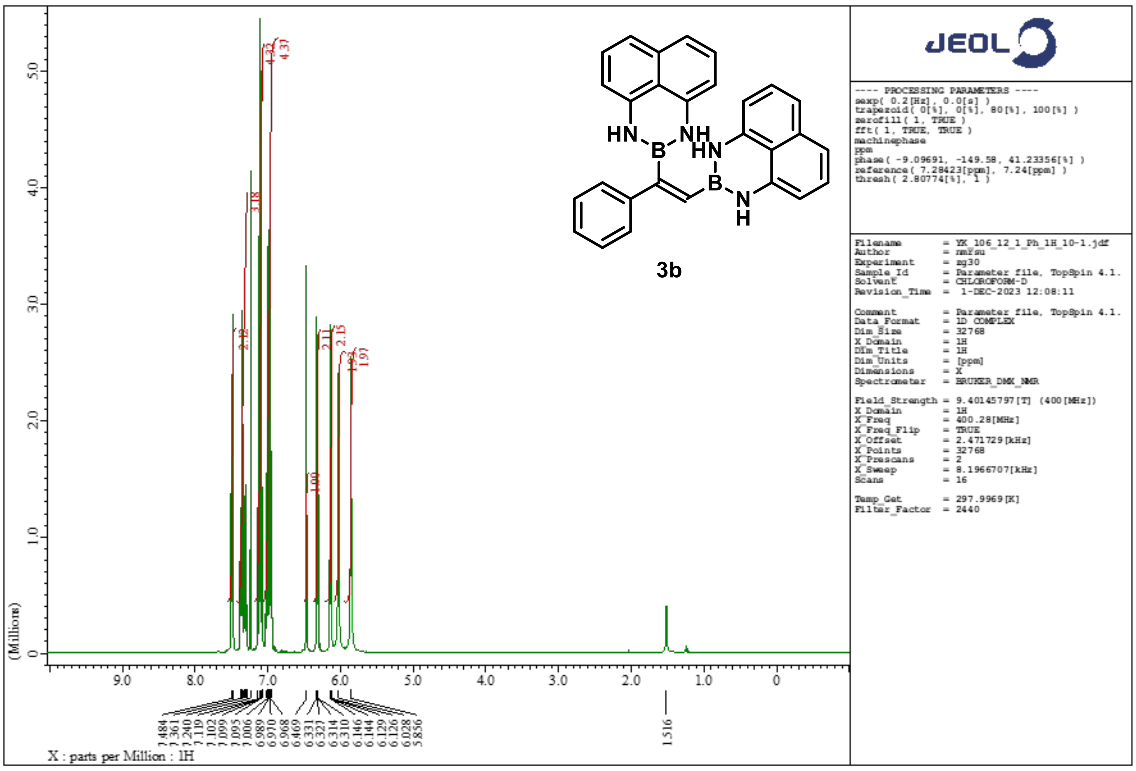

Figure S4. ^{1}H NMR ($CDCl_3$, 400 MHz) spectrum of **3b**.

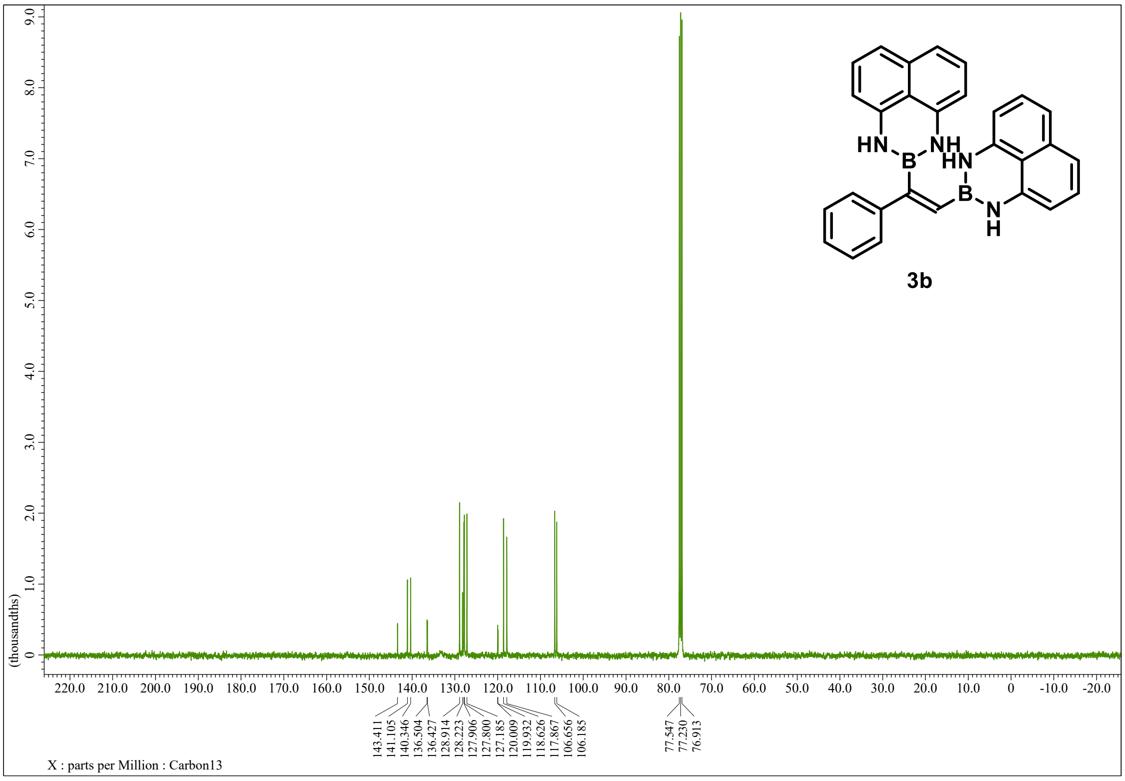

Figure S5. $^{13}C\{^{1}H\}$ NMR ($CDCl_3$, 100 MHz) spectrum of **3b**.

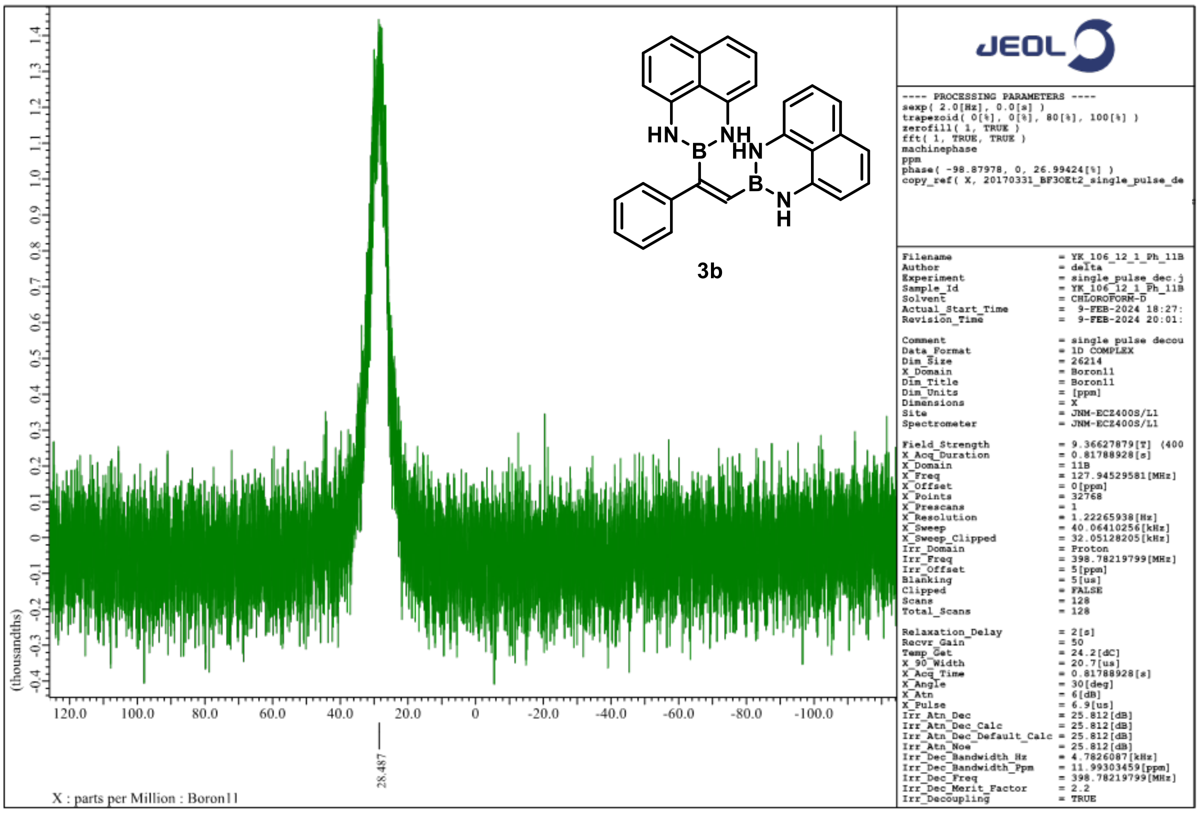

Figure S6. $^{11}B\{^{1}H\}$ NMR ($CDCl_3$, 128 MHz) spectrum of **3b**.

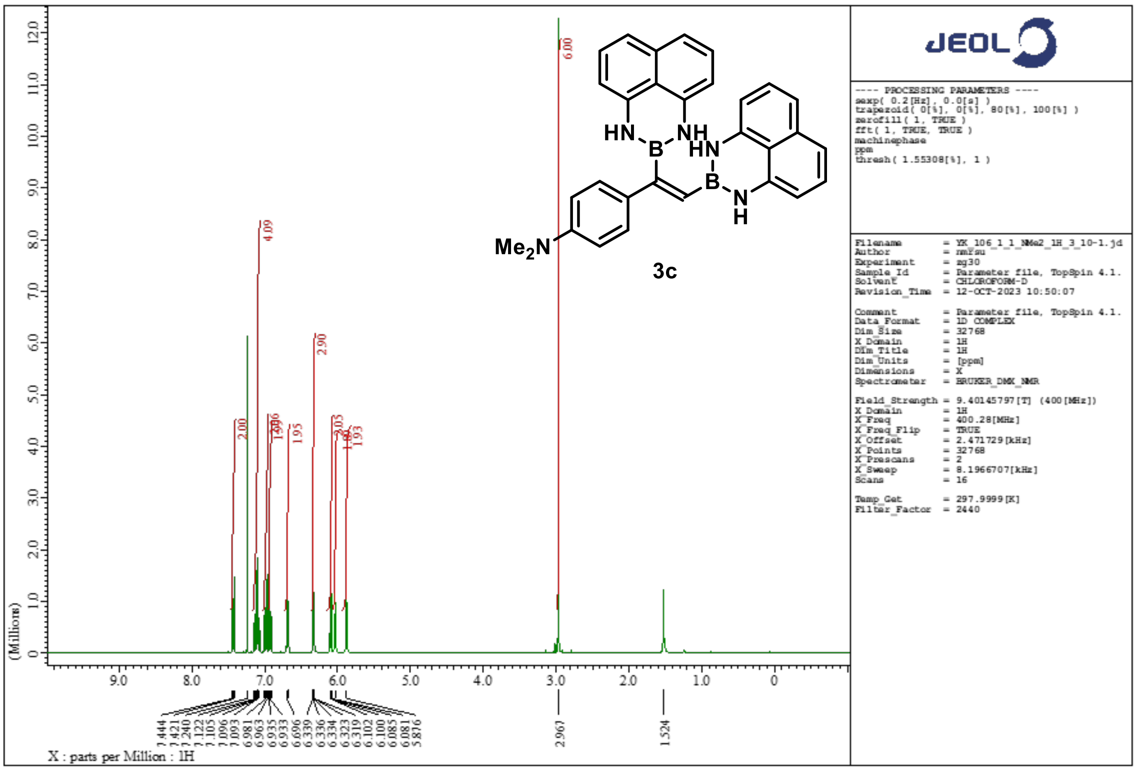

Figure S7. ^{1}H NMR ($CDCl_3$, 400 MHz) spectrum of **3c**.

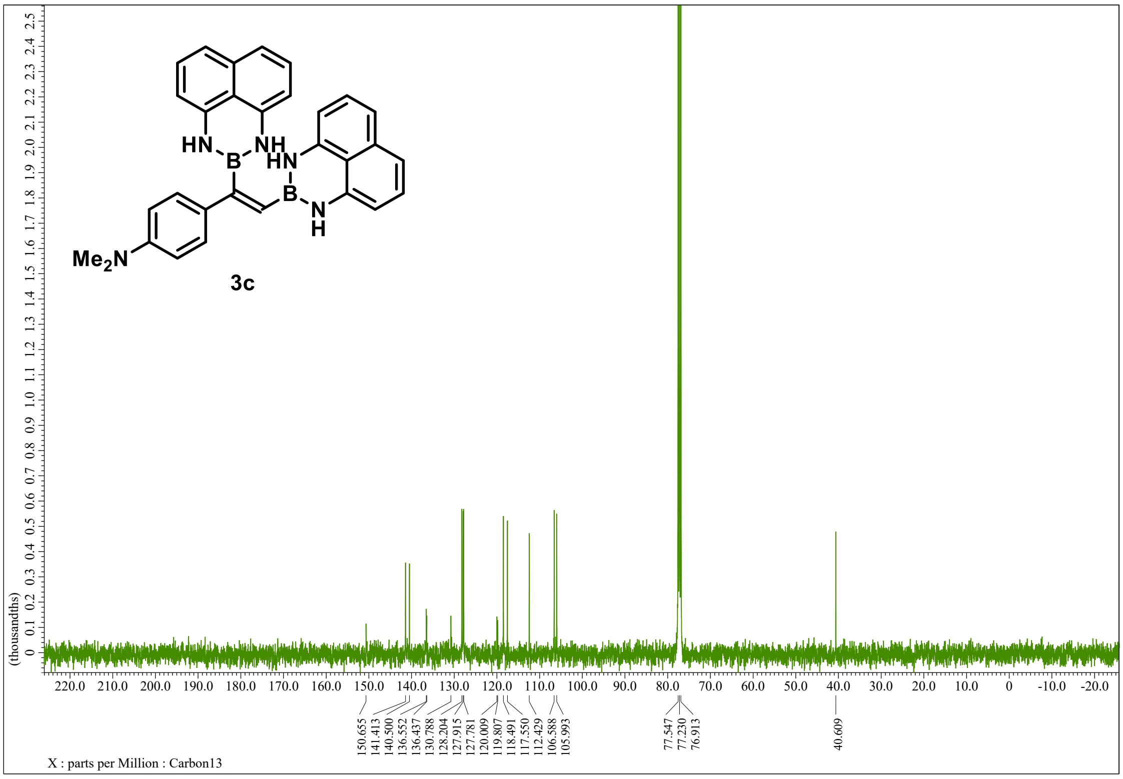

Figure S8. $^{13}C\{^{1}H\}$ NMR ($CDCl_3$, 100 MHz) spectrum of **3c**.

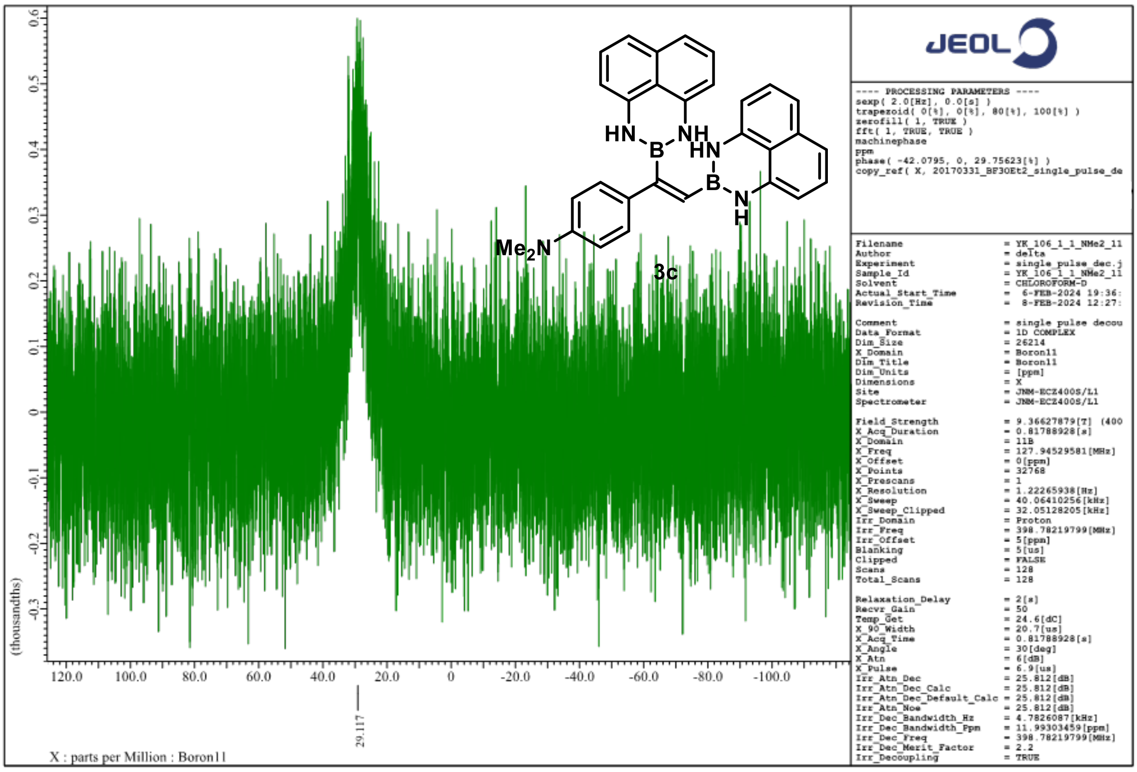

Figure S9. $^{11}B\{^{1}H\}$ NMR ($CDCl_3$, 128 MHz) spectrum of **3c**.

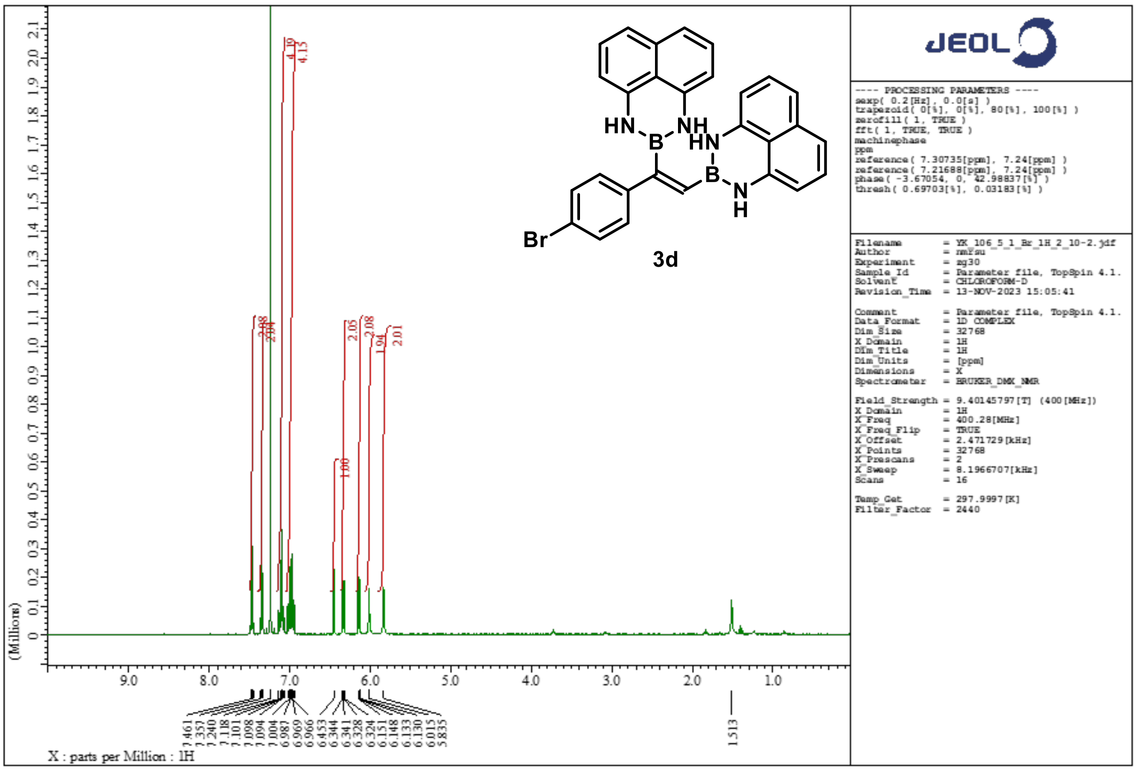

Figure S10. ^{1}H NMR ($CDCl_3$, 400 MHz) spectrum of **3d**.

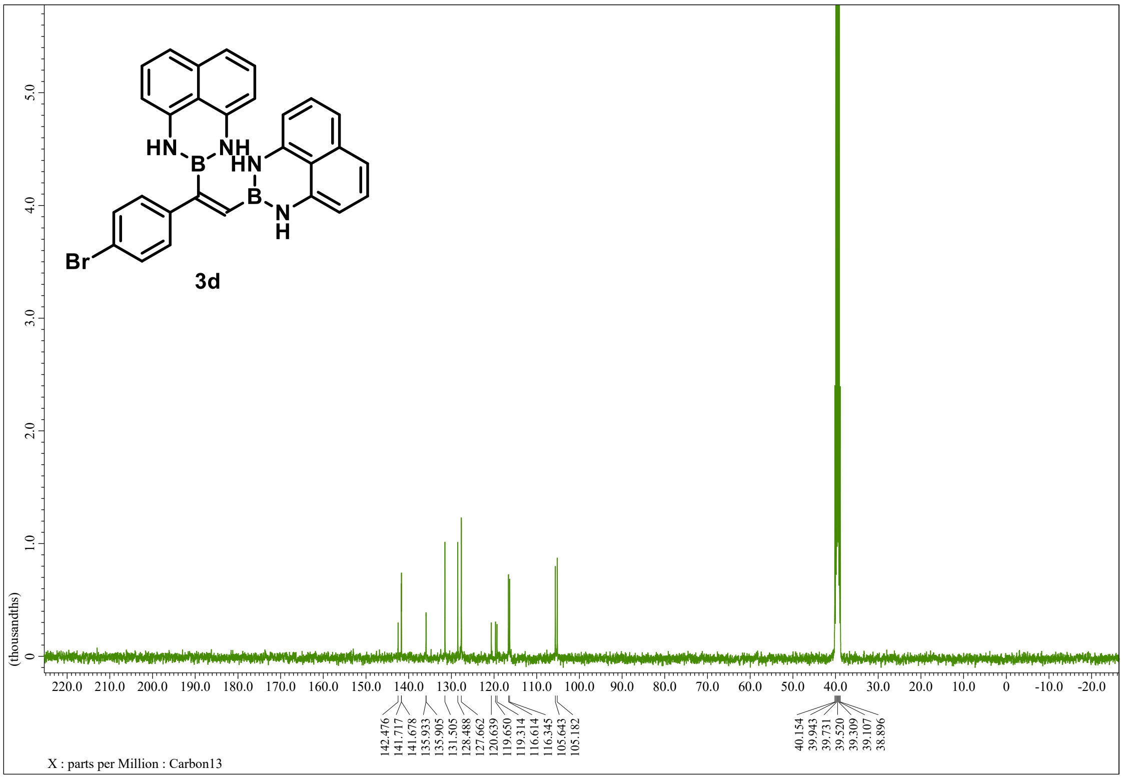

Figure S11. $^{13}C\{^{1}H\}$ NMR (DMSO-d_6, 100 MHz) spectrum of **3d**.

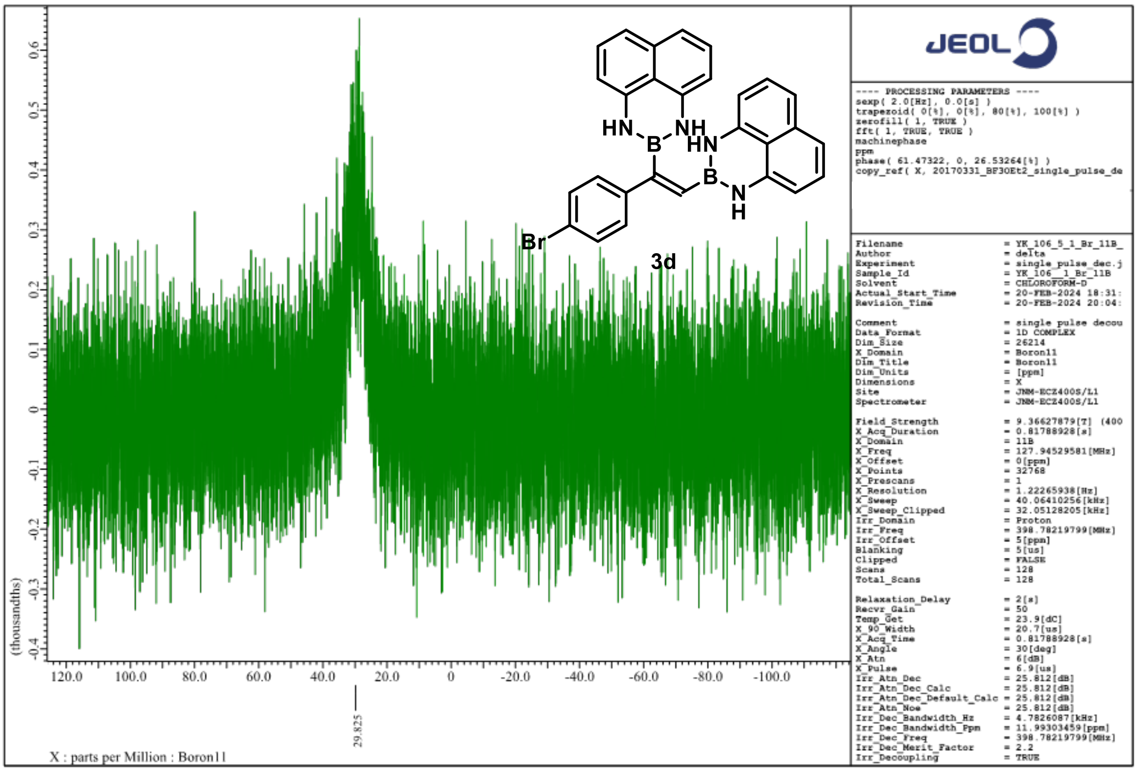

Figure S12. $^{11}B\{^1H\}$ NMR ($CDCl_3$, 128 MHz) spectrum of **3d**.

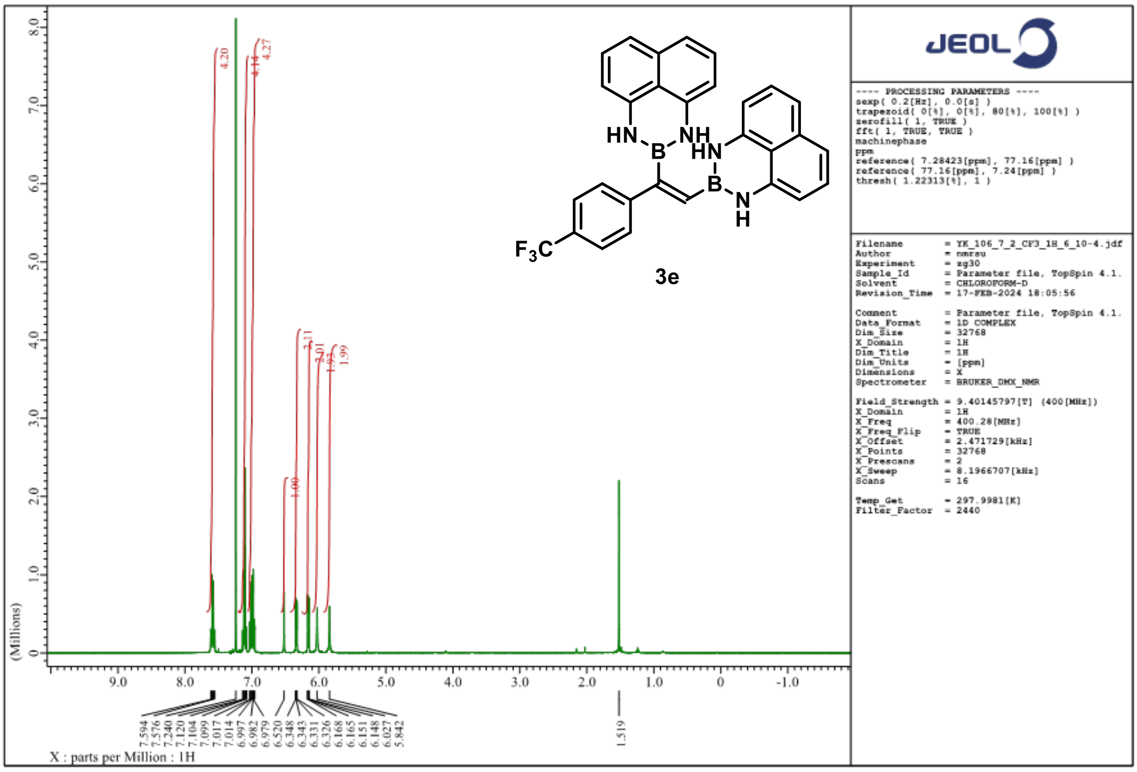

Figure S13. ^{1}H NMR ($CDCl_3$, 400 MHz) spectrum of **3e**.

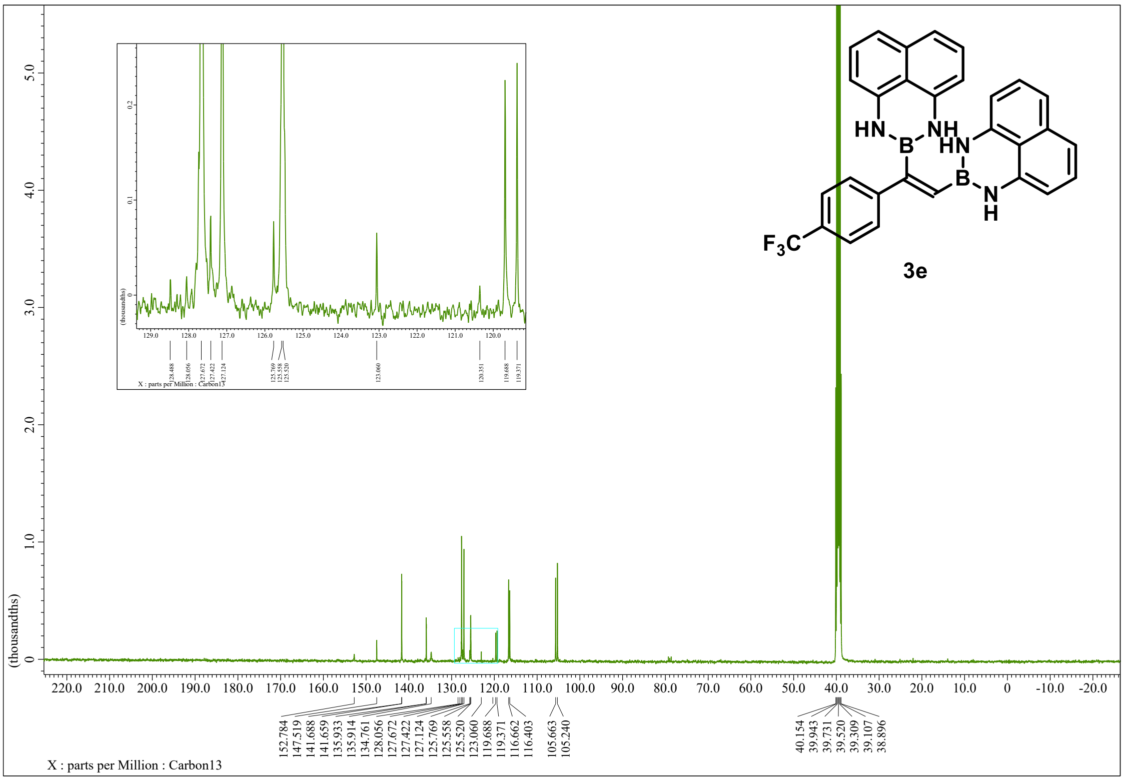

Figure S14. $^{13}C\{^{1}H\}$ NMR (DMSO-d_6, 100 MHz) spectrum of **3e**.

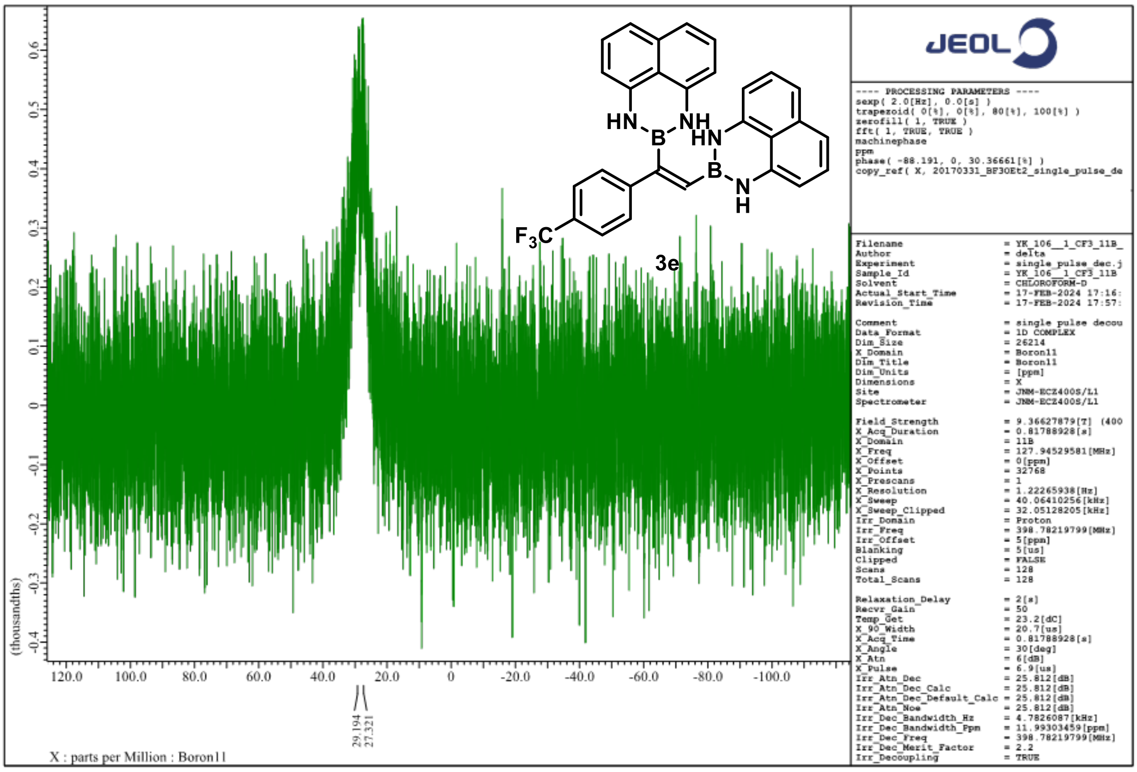

Figure S15. $^{11}B\{^{1}H\}$ NMR ($CDCl_3$, 128 MHz) spectrum of **3e**.

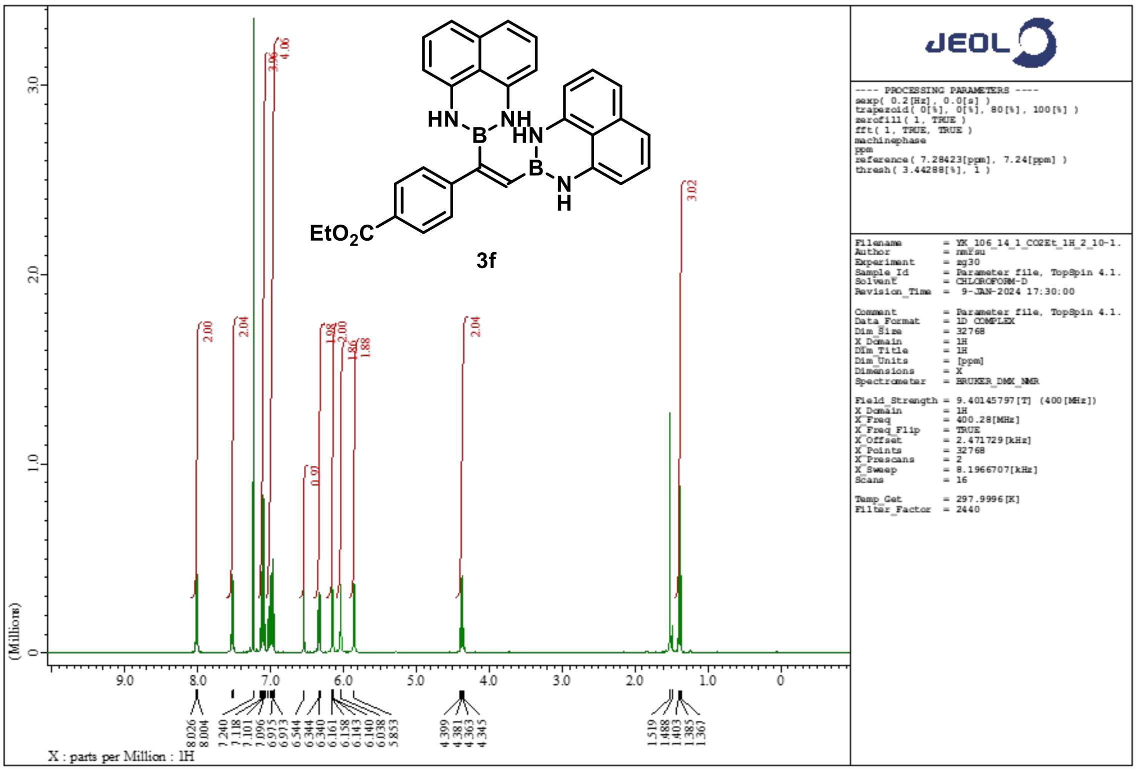

Figure S16. ^{1}H NMR ($CDCl_3$, 400 MHz) spectrum of **3f**.

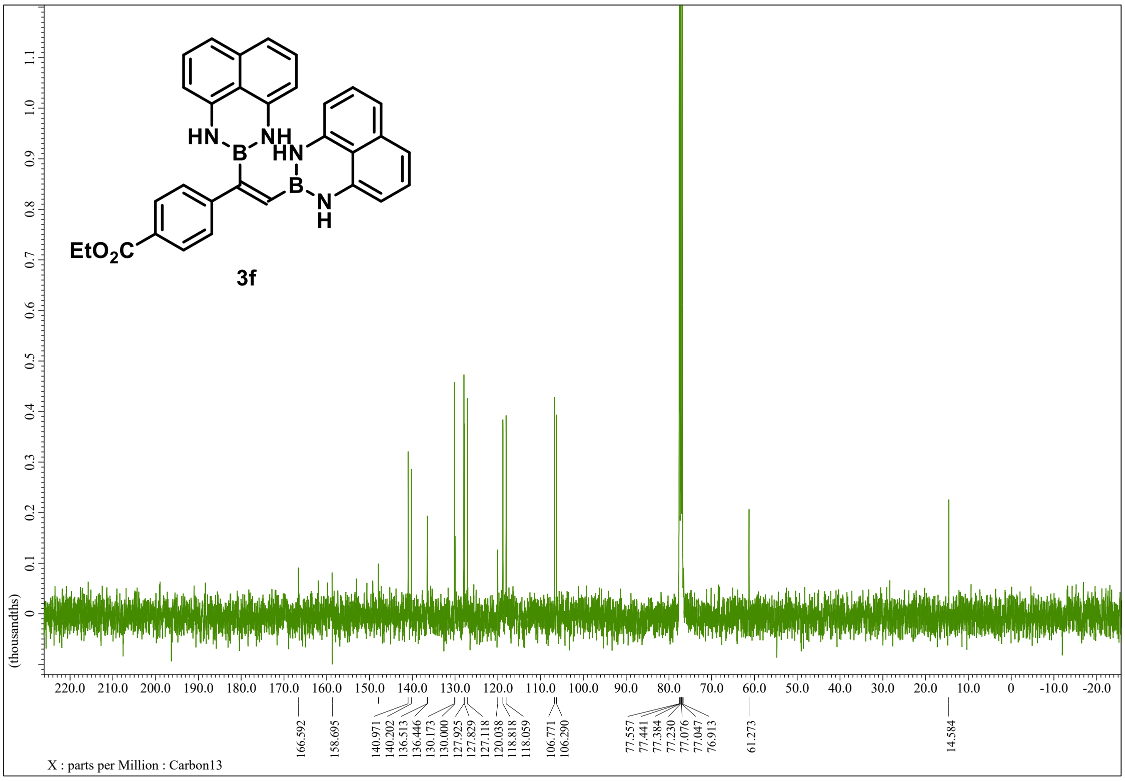

Figure S17. $^{13}C\{^{1}H\}$ NMR ($CDCl_3$, 100 MHz) spectrum of **3f**.

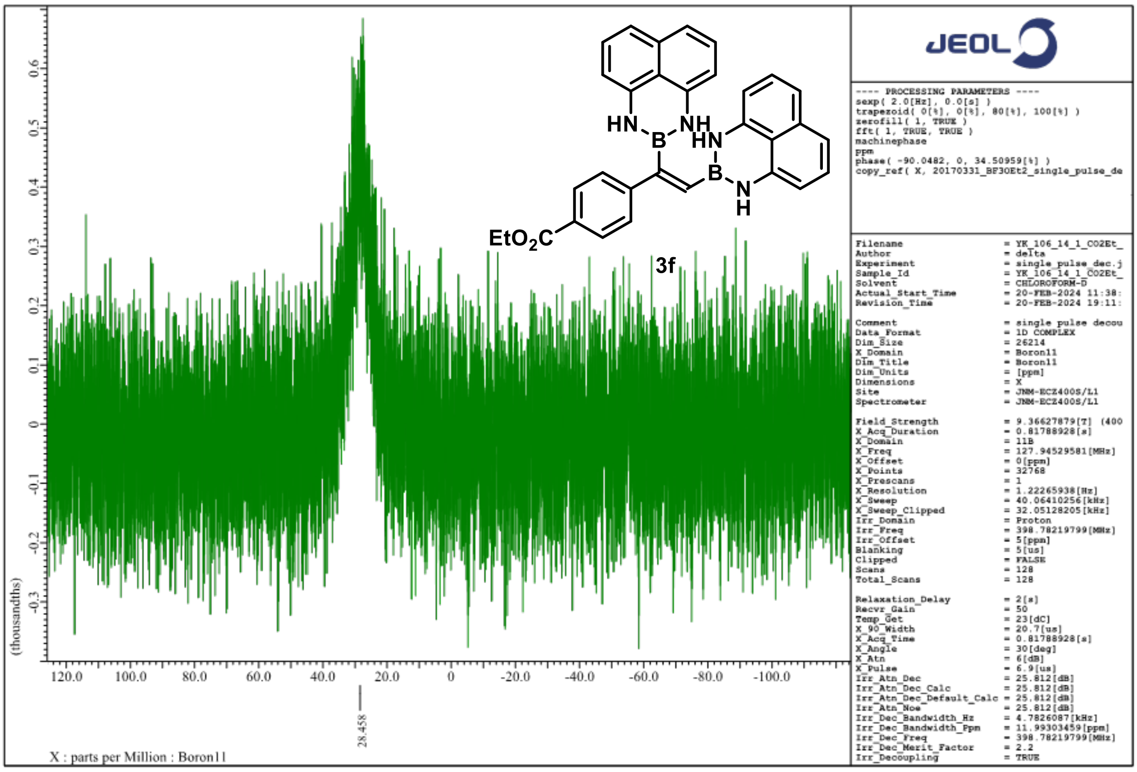

Figure S18. $^{11}B\{^1H\}$ NMR ($CDCl_3$, 128 MHz) spectrum of **3f**.

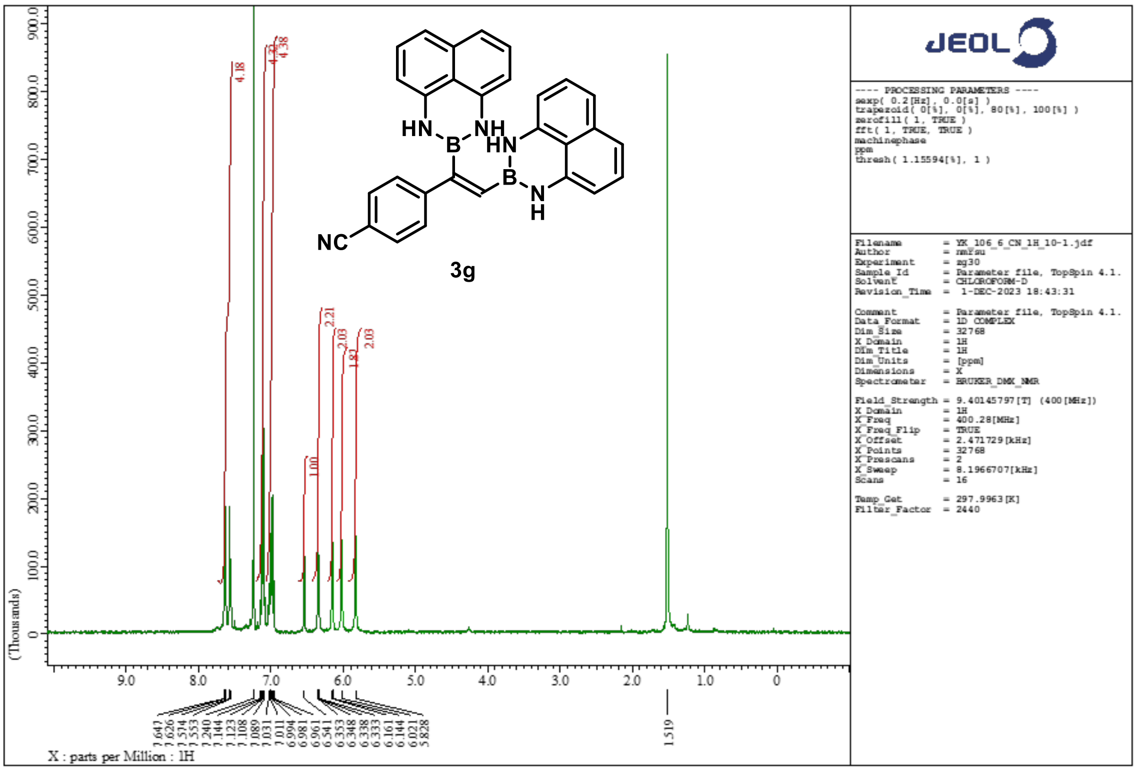

Figure S19. ^{1}H NMR ($CDCl_3$, 400 MHz) spectrum of **3g**.

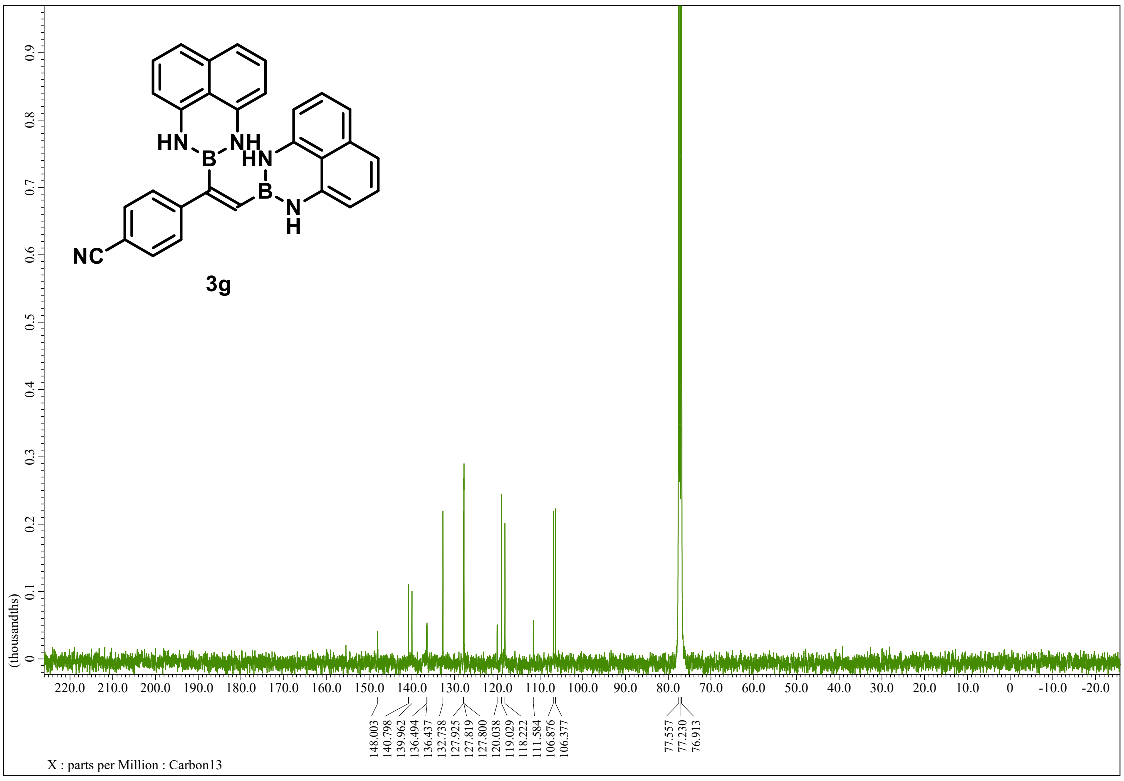

Figure S20. $^{13}C\{^{1}H\}$ NMR ($CDCl_3$, 100 MHz) spectrum of **3g**.

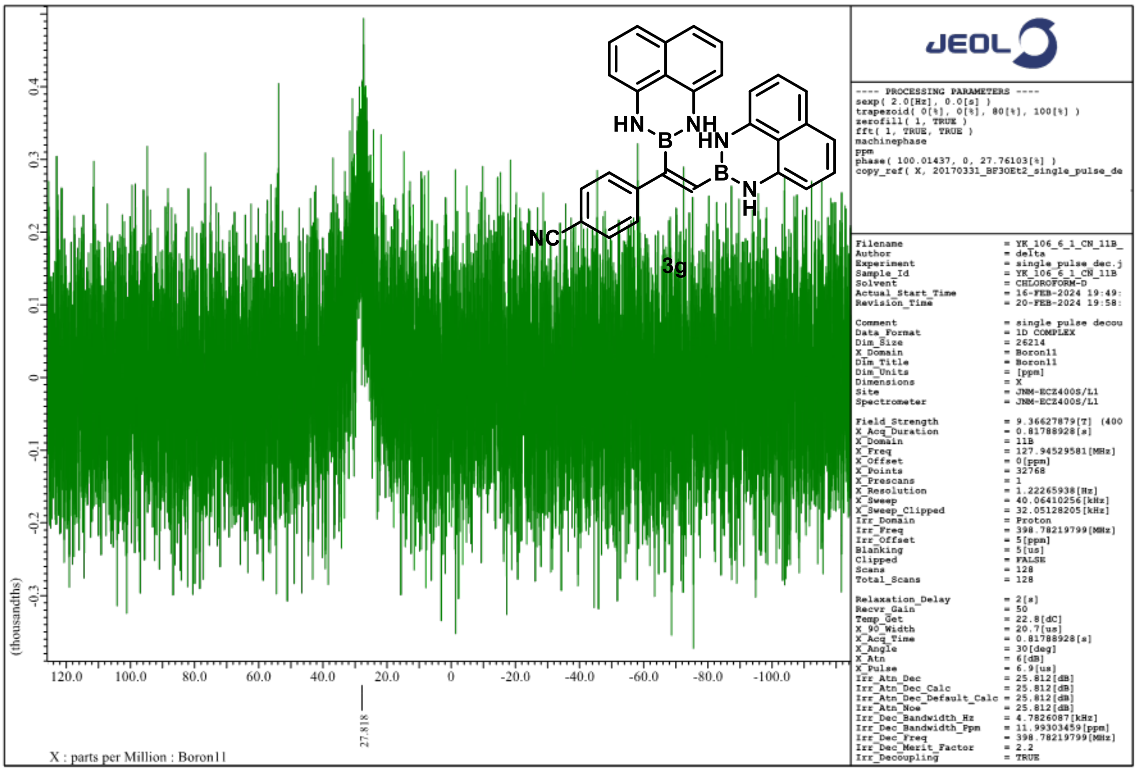

Figure S21. $^{11}B\{^{1}H\}$ NMR ($CDCl_3$, 128 MHz) spectrum of **3g**.

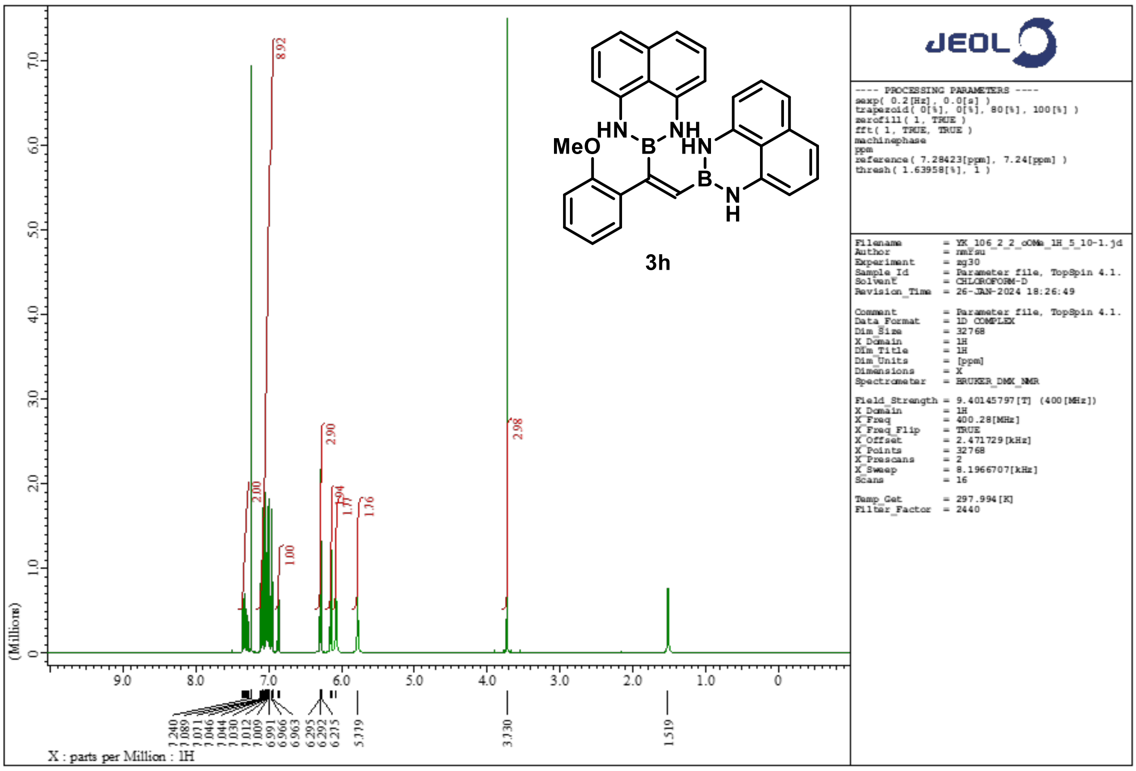

Figure S22. ^{1}H NMR ($CDCl_3$, 400 MHz) spectrum of **3h**.

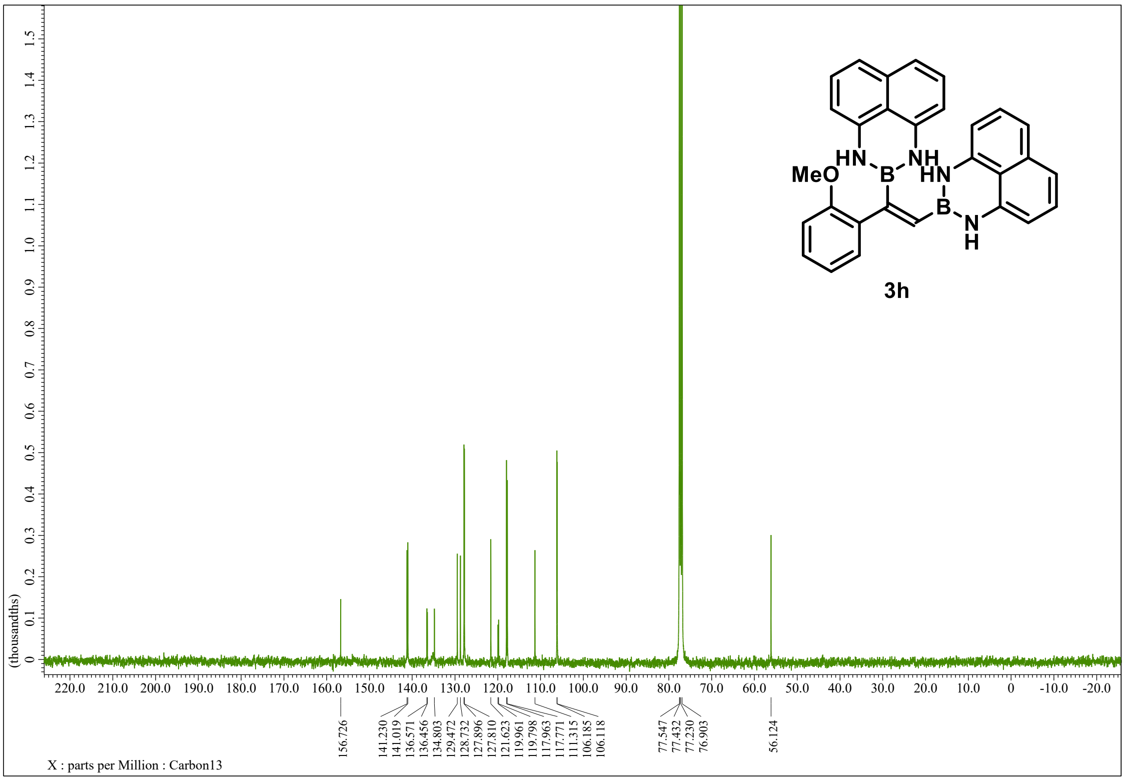

Figure S23. $^{13}C\{^{1}H\}$ NMR ($CDCl_3$, 100 MHz) spectrum of **3h**.

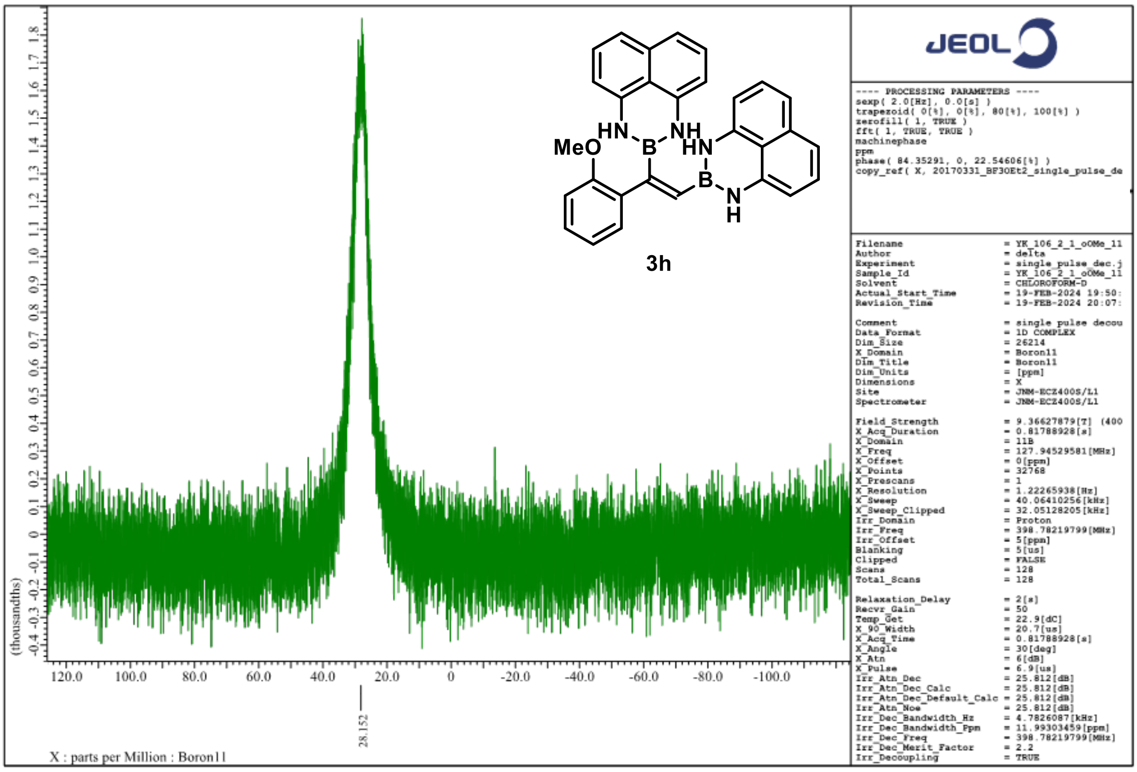

Figure S24. $^{11}B\{^{1}H\}$ NMR ($CDCl_3$, 128 MHz) spectrum of **3h**.

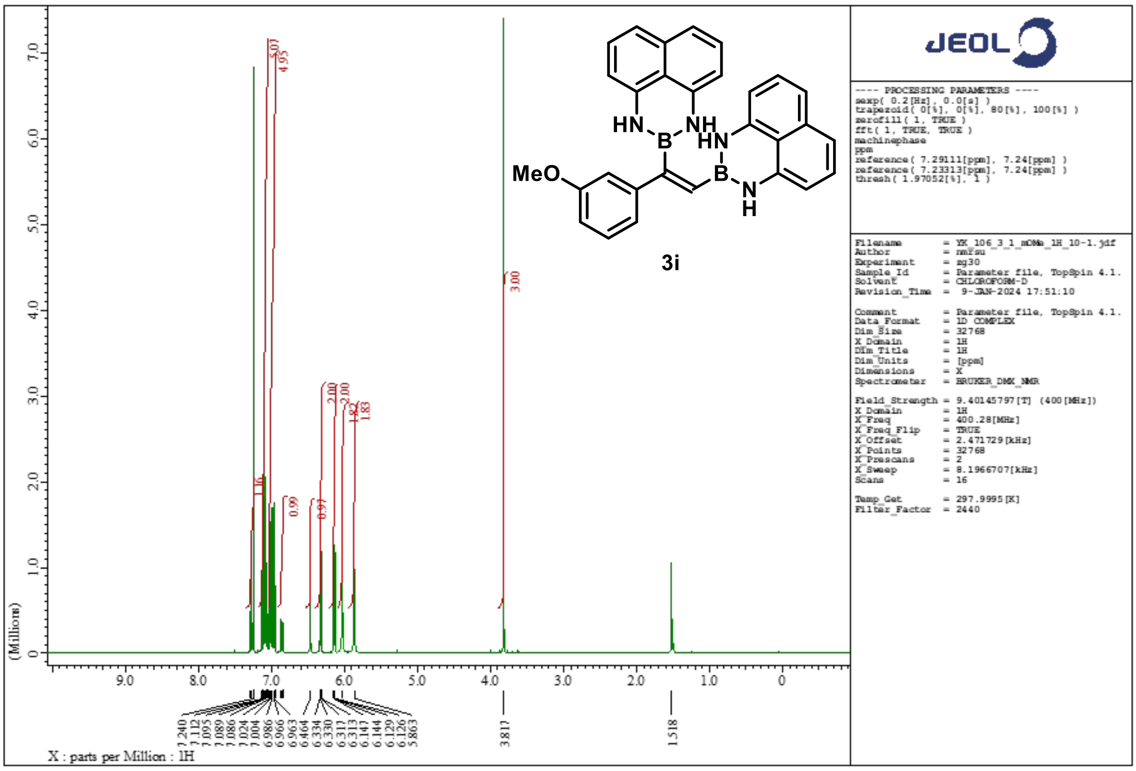

Figure S25. ^{1}H NMR ($CDCl_3$, 400 MHz) spectrum of **3i**.

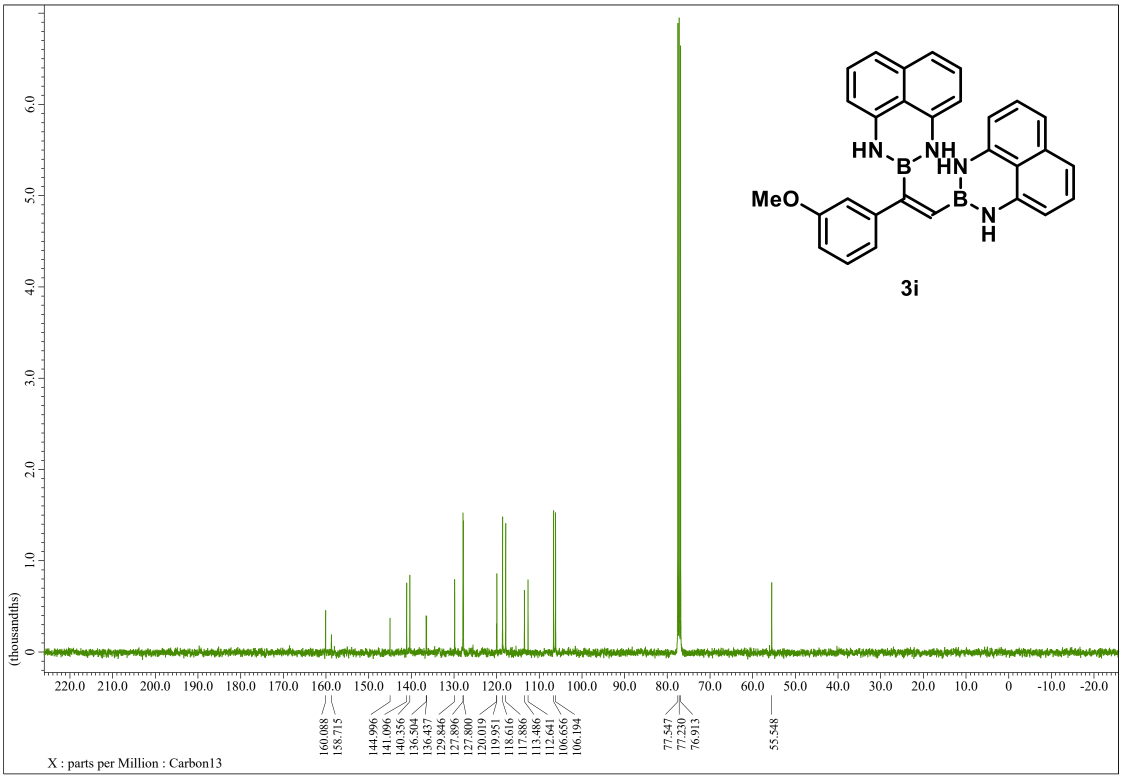

Figure S26. $^{13}C\{^{1}H\}$ NMR ($CDCl_3$, 100 MHz) spectrum of **3i**.

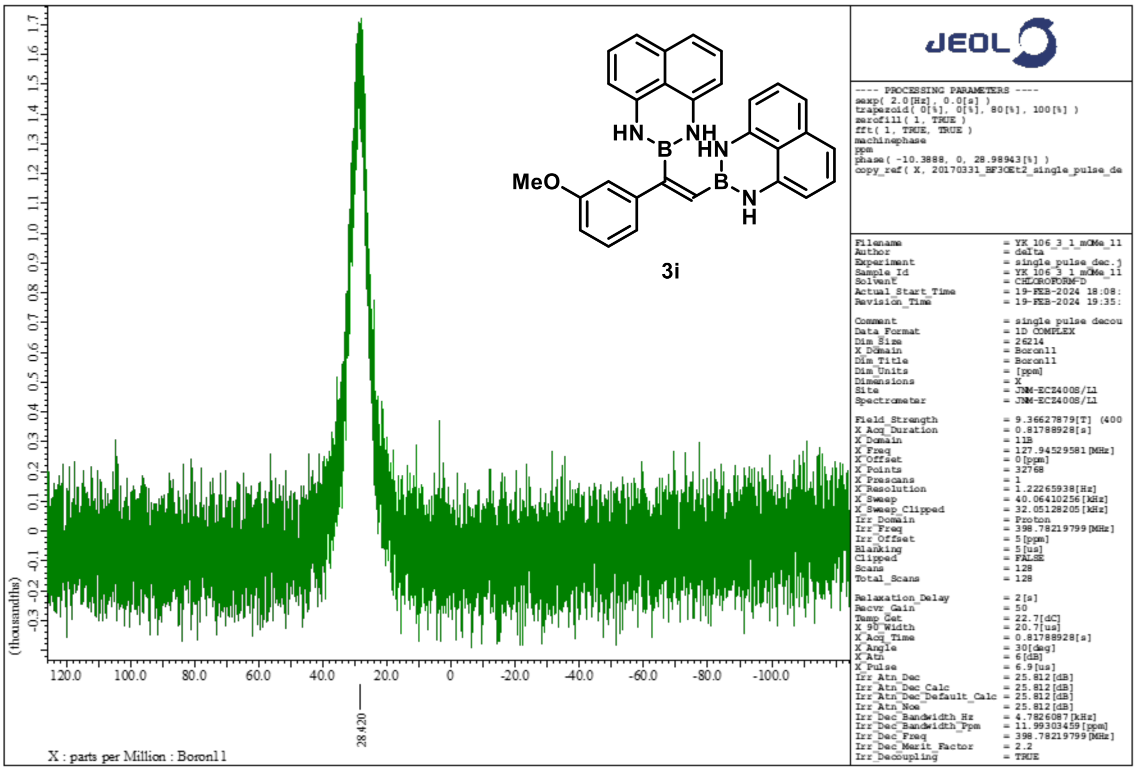

Figure S27. $^{11}B\{^1H\}$ NMR ($CDCl_3$, 128 MHz) spectrum of **3i**.

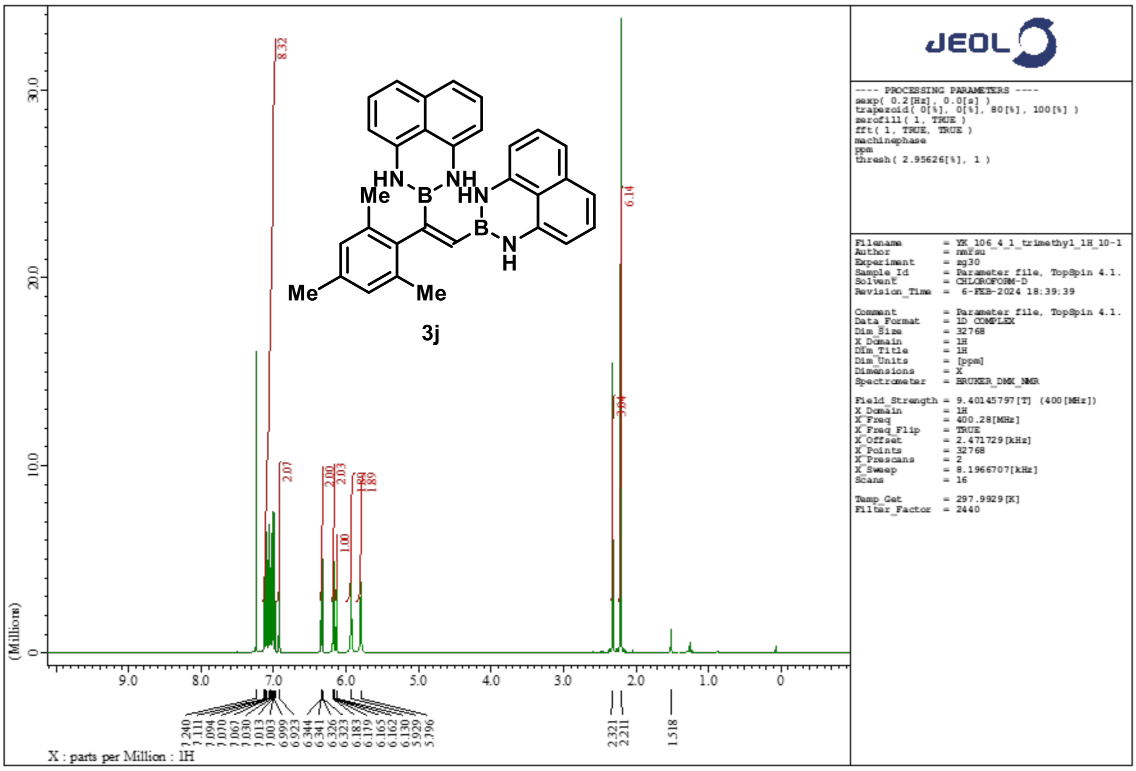

Figure S28. ^{1}H NMR ($CDCl_3$, 400 MHz) spectrum of **3j**.

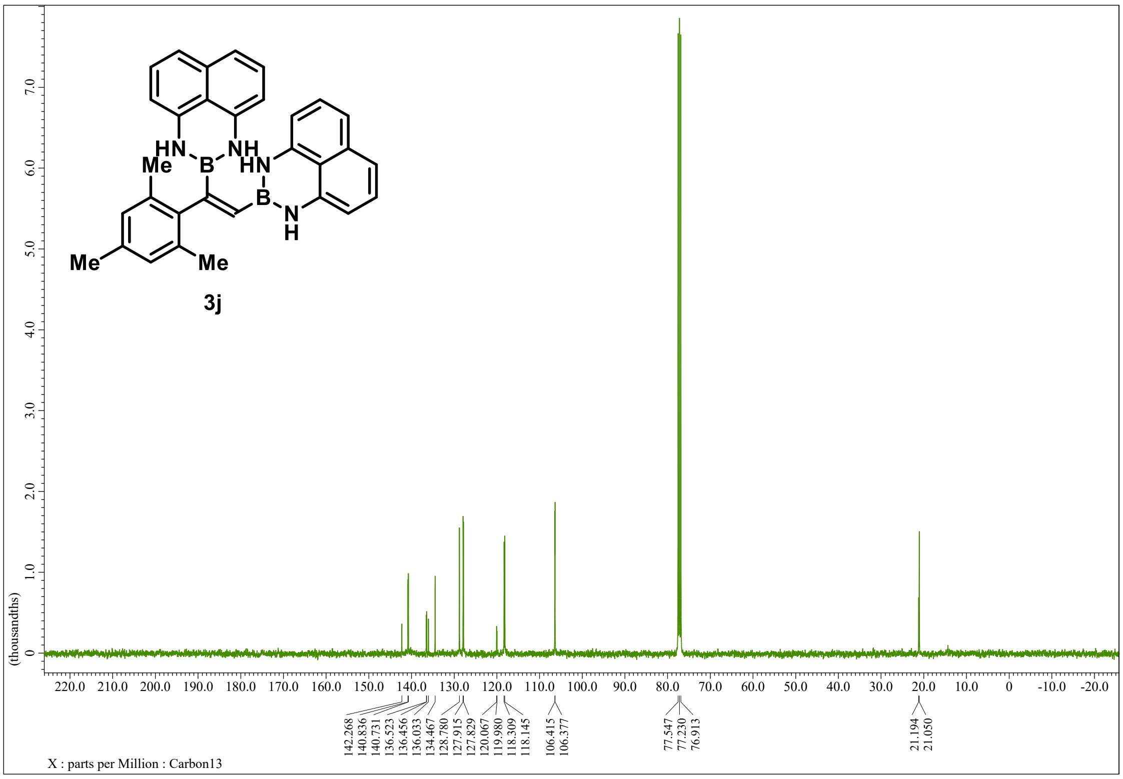

Figure S29. $^{13}C\{^{1}H\}$ NMR ($CDCl_3$, 100 MHz) spectrum of **3j**.

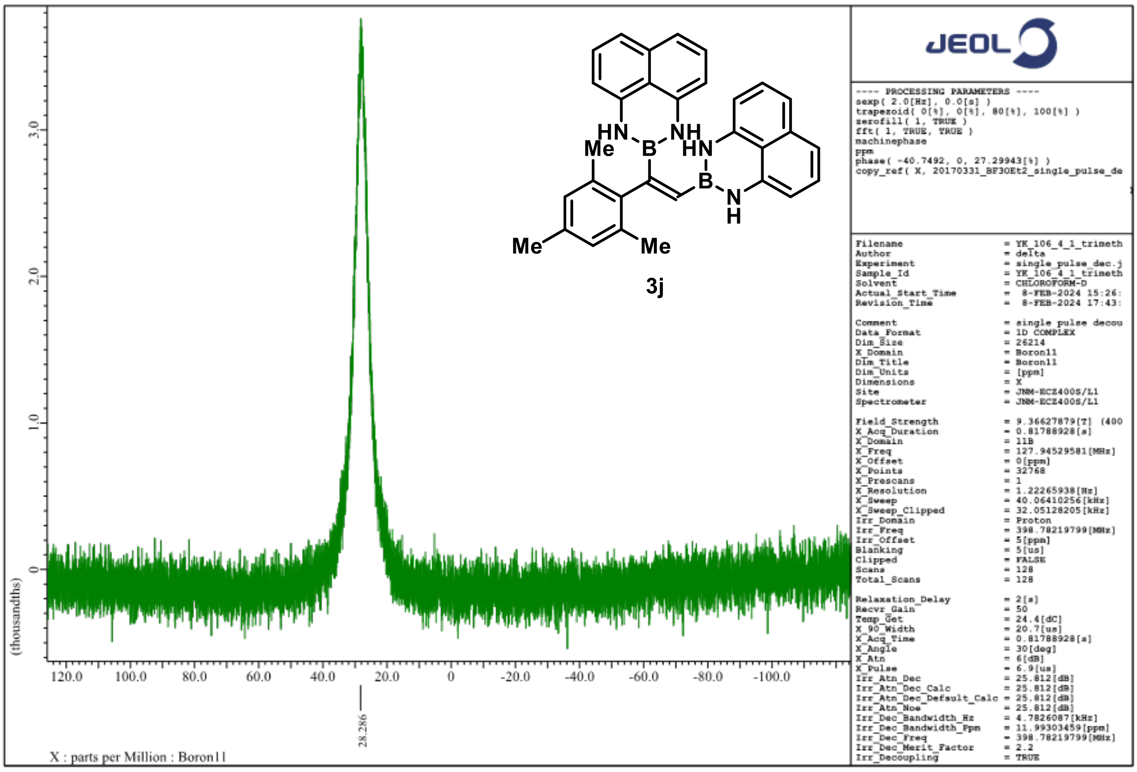

Figure S30. $^{11}B\{^1H\}$ NMR ($CDCl_3$, 128 MHz) spectrum of **3j**.

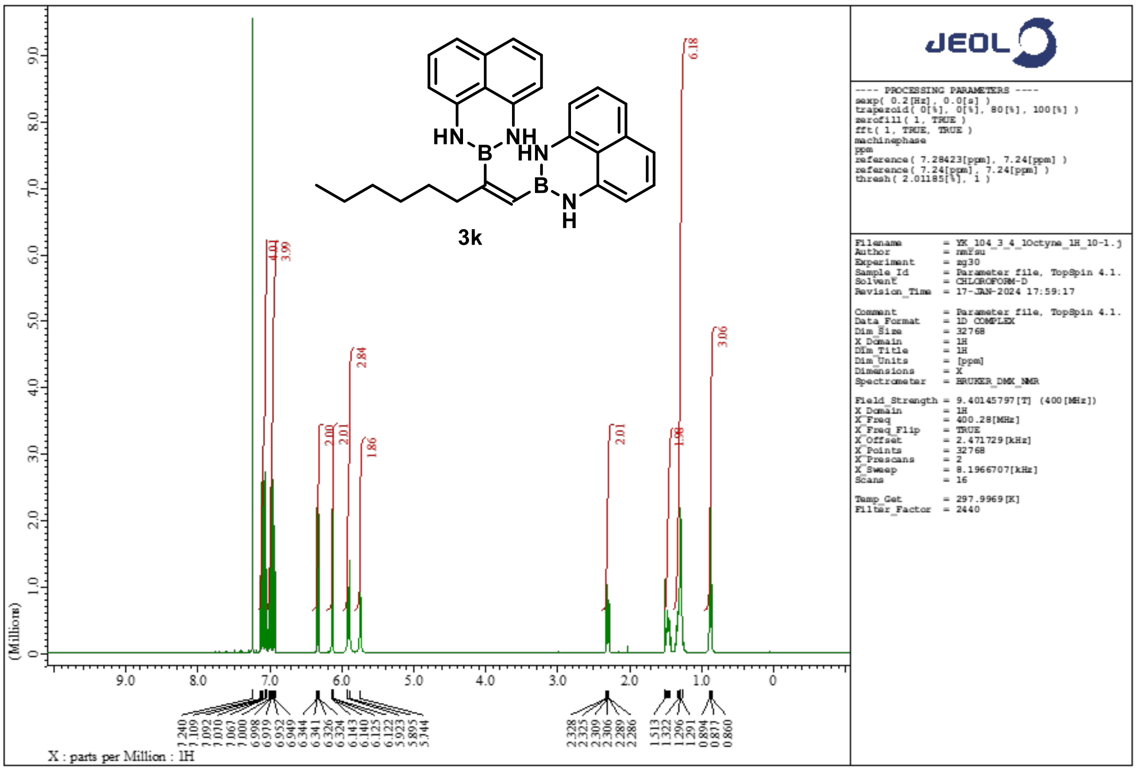

Figure S31. ^{1}H NMR ($CDCl_3$, 400 MHz) spectrum of **3k**.

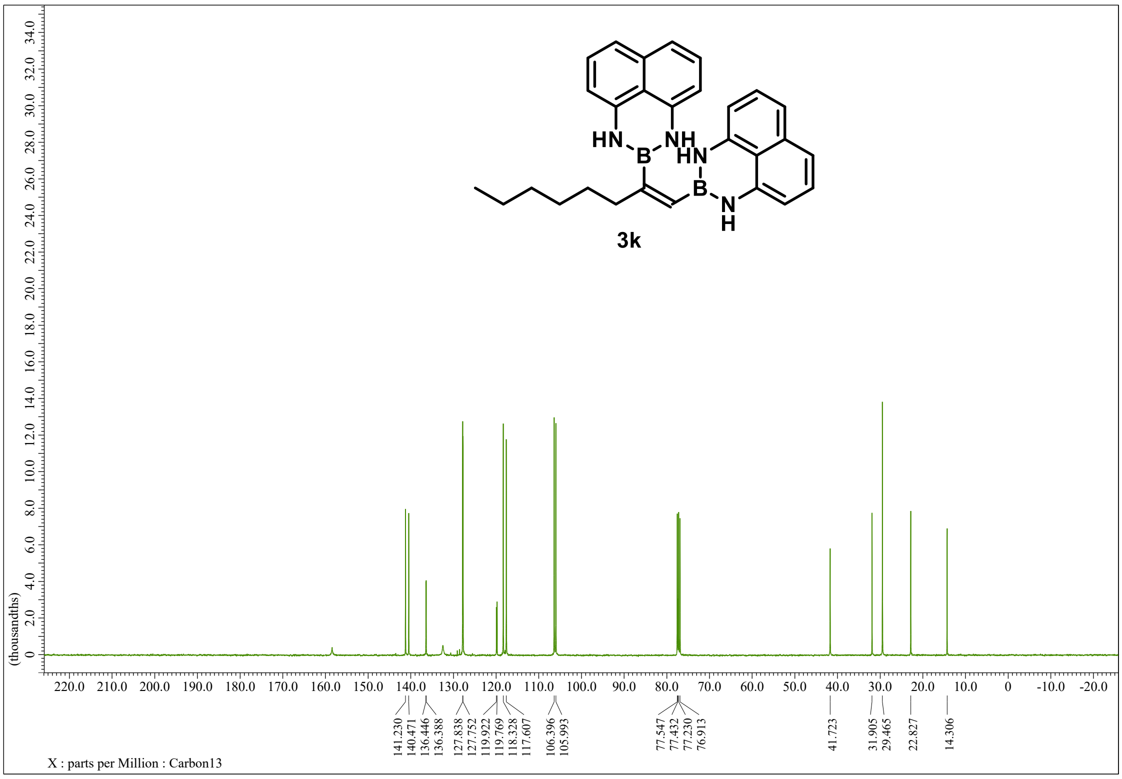

Figure S32. $^{13}C\{^{1}H\}$ NMR ($CDCl_3$, 100 MHz) spectrum of **3k**.

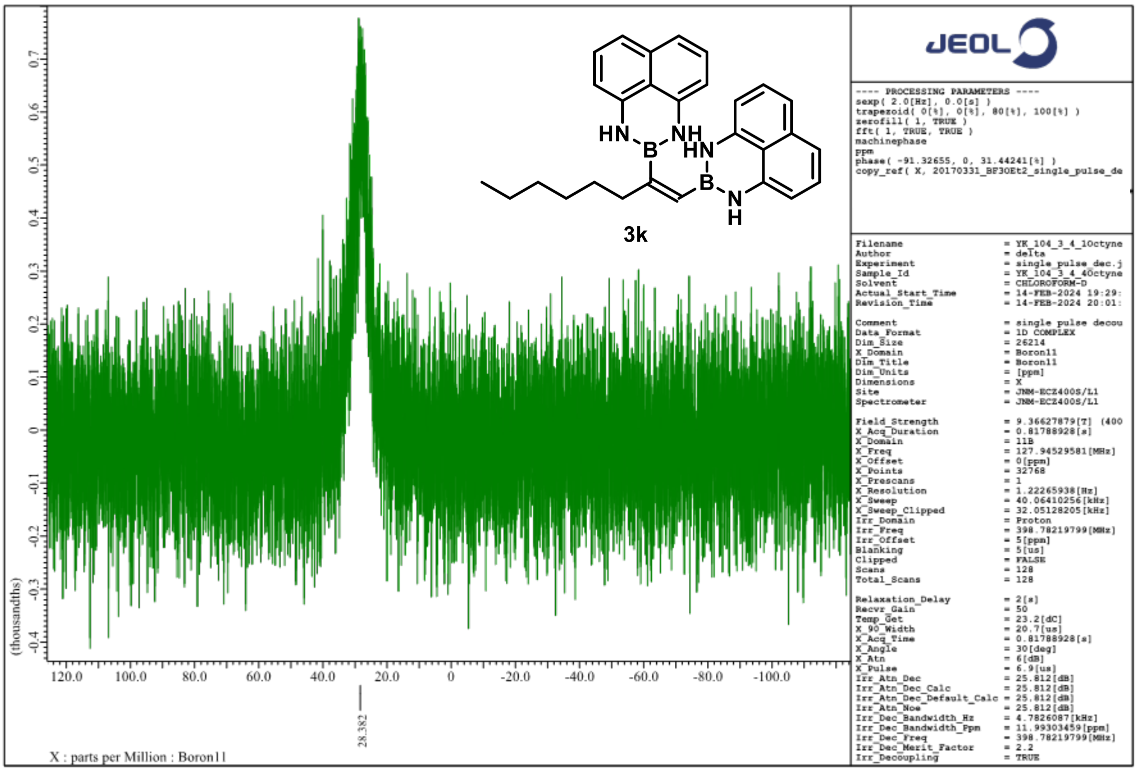

Figure S33. $^{11}B\{^{1}H\}$ NMR ($CDCl_3$, 128 MHz) spectrum of **3k**.

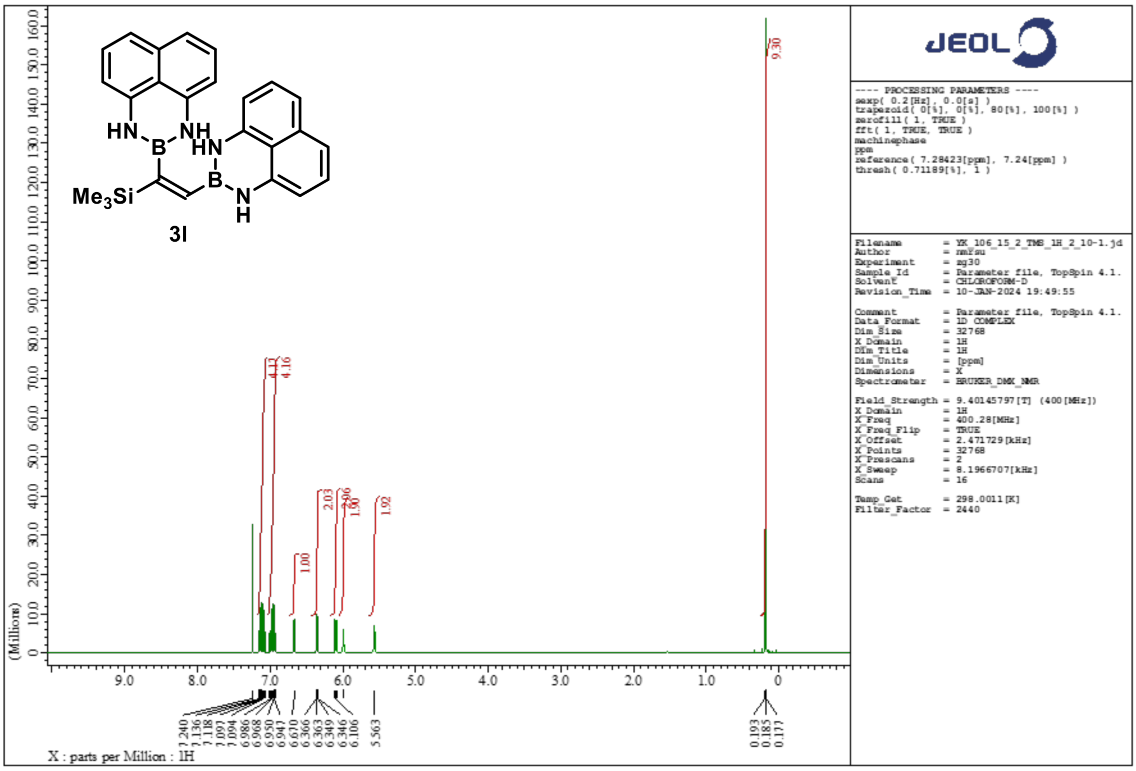

Figure S34. ^{1}H NMR ($CDCl_3$, 400 MHz) spectrum of **3l**.

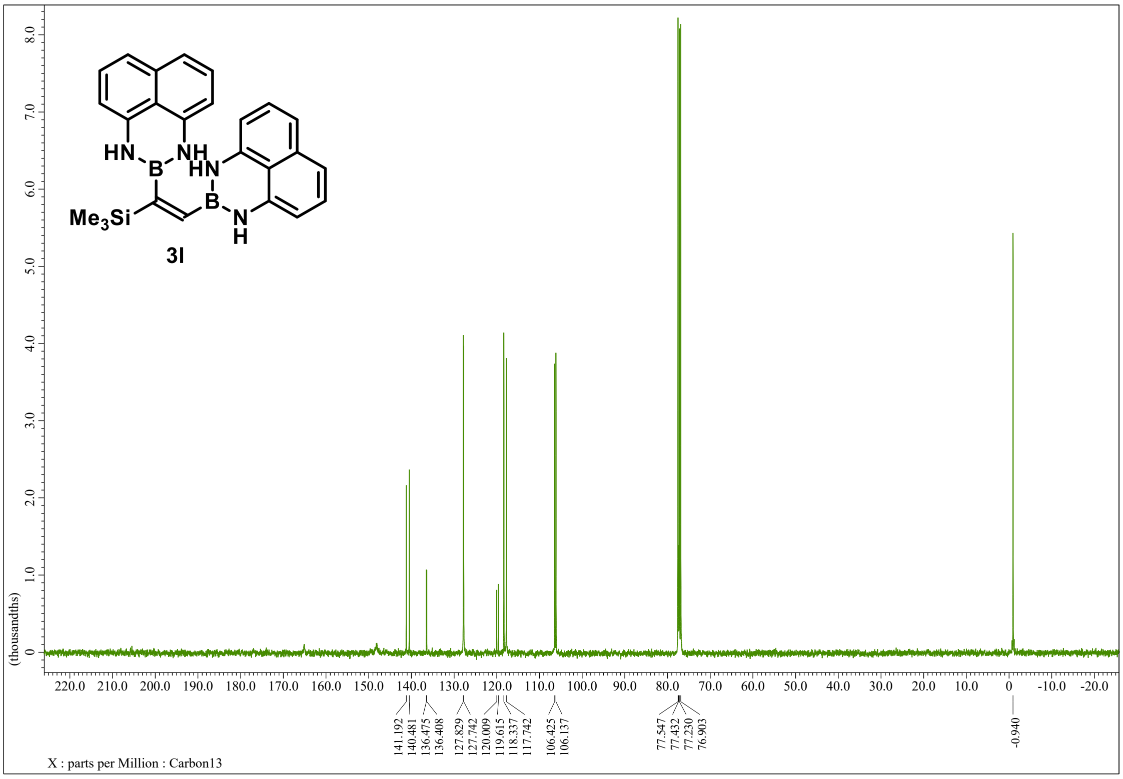

Figure S35. $^{13}C\{^{1}H\}$ NMR ($CDCl_3$, 100 MHz) spectrum of **3l**.

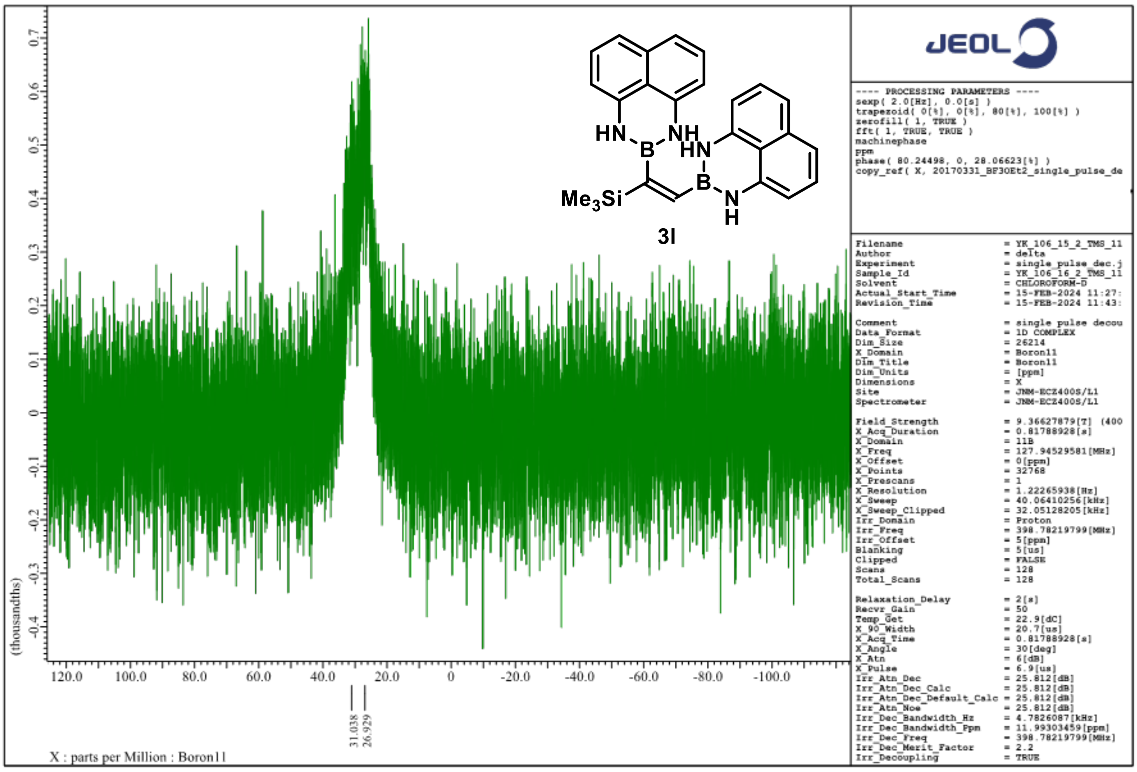

Figure S36. $^{11}B\{^{1}H\}$ NMR ($CDCl_3$, 128 MHz) spectrum of **3l**.

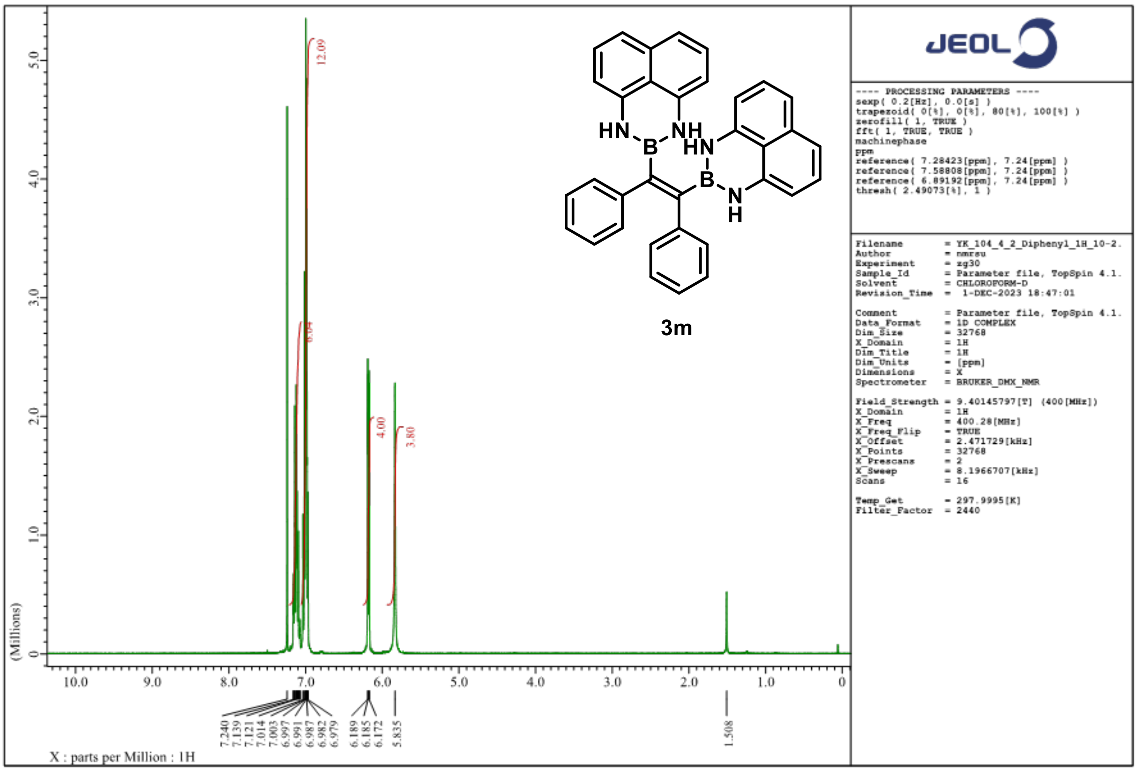

Figure S37. ^{1}H NMR ($CDCl_3$, 400 MHz) spectrum of **3m**.

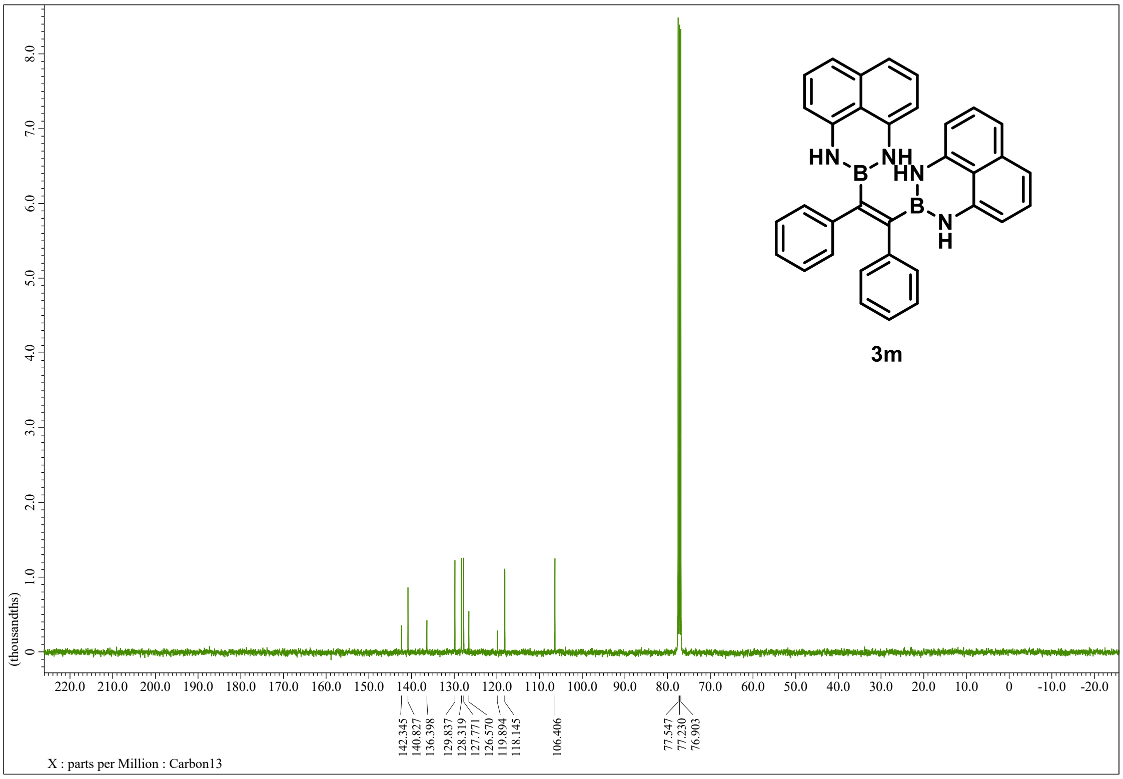

Figure S38. ^{13}C{^{1}H} NMR ($CDCl_3$, 100 MHz) spectrum of **3m**.

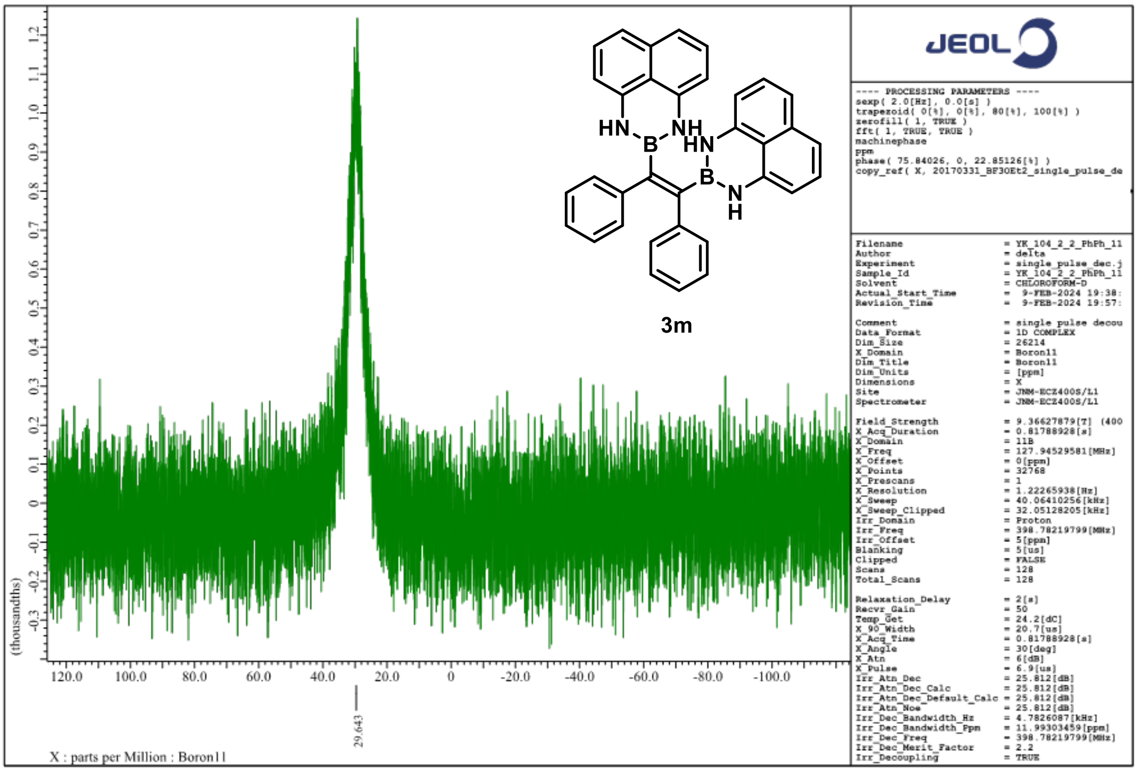

Figure S39. $^{11}B\{^{1}H\}$ NMR ($CDCl_3$, 128 MHz) spectrum of **3m**.

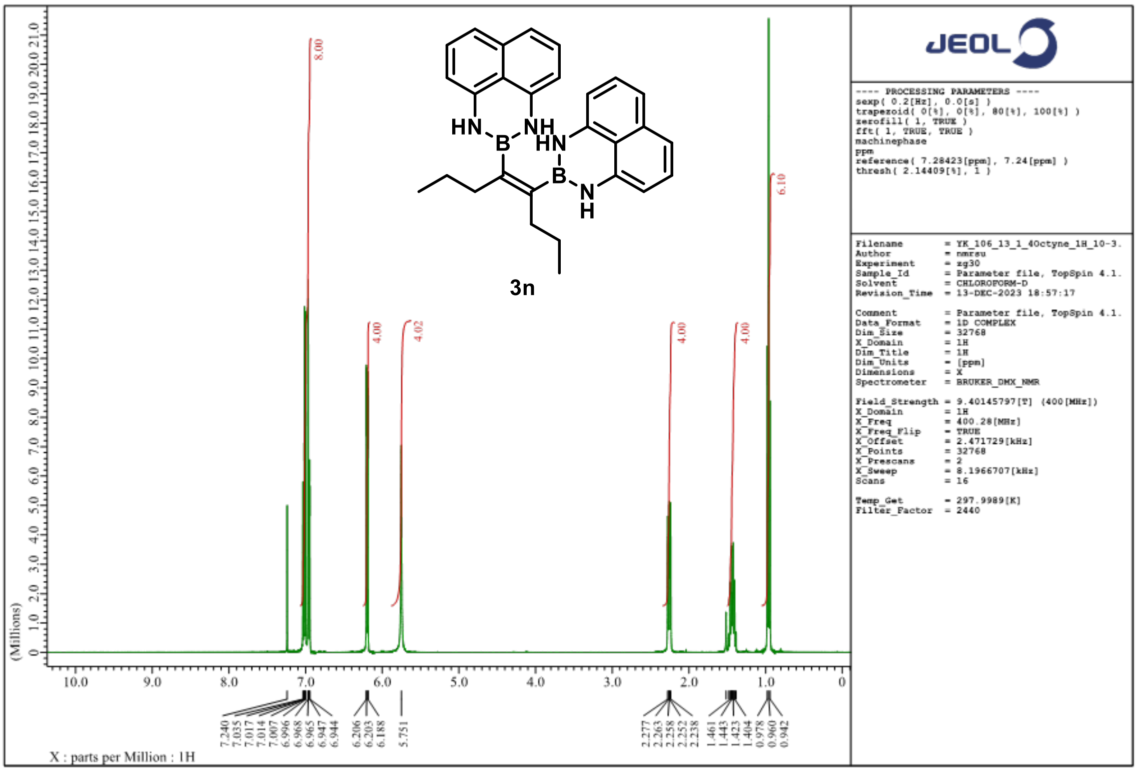

Figure S40. ^{1}H NMR ($CDCl_3$, 400 MHz) spectrum of **3n**.

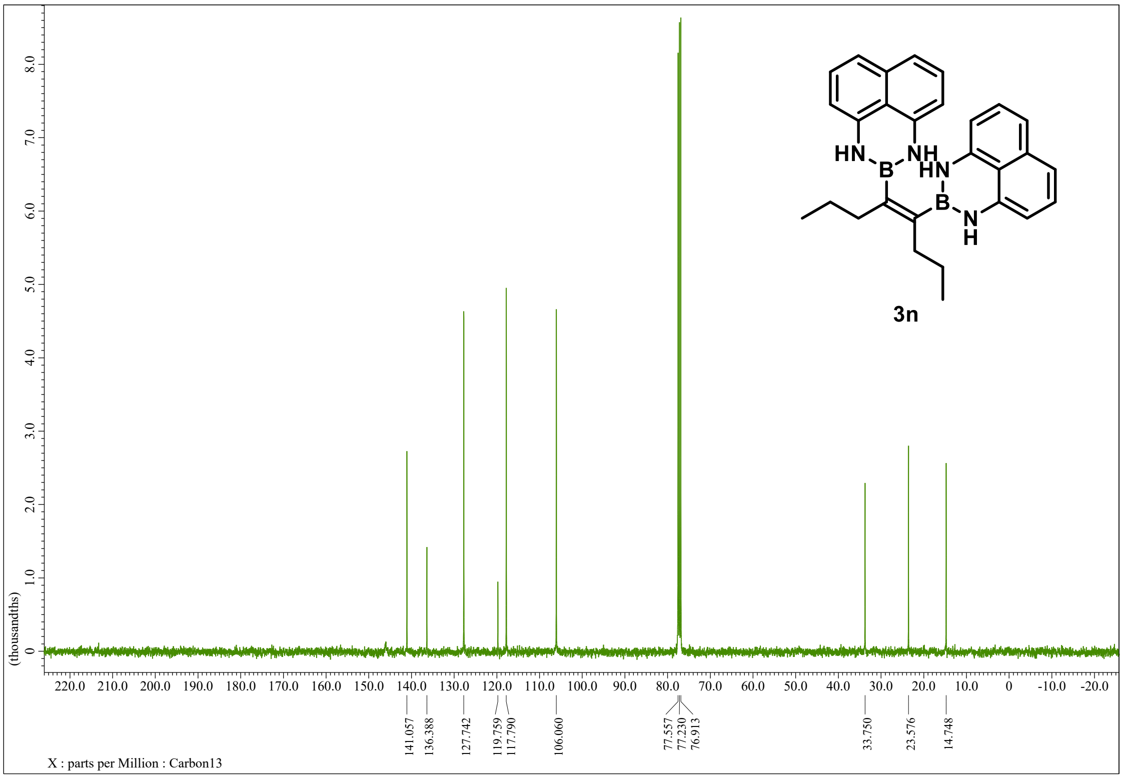

Figure S41. $^{13}C\{^{1}H\}$ NMR ($CDCl_3$, 100 MHz) spectrum of **3n**.

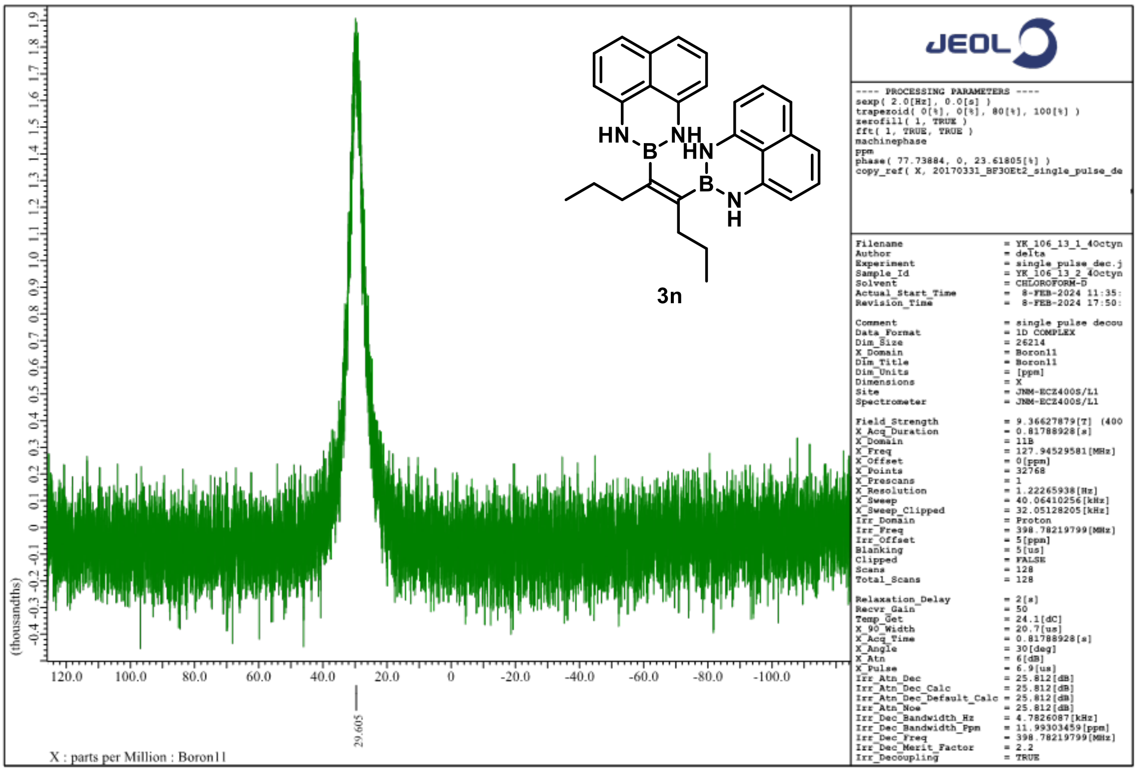

Figure S42. $^{11}B\{^1H\}$ NMR ($CDCl_3$, 128 MHz) spectrum of **3n**.

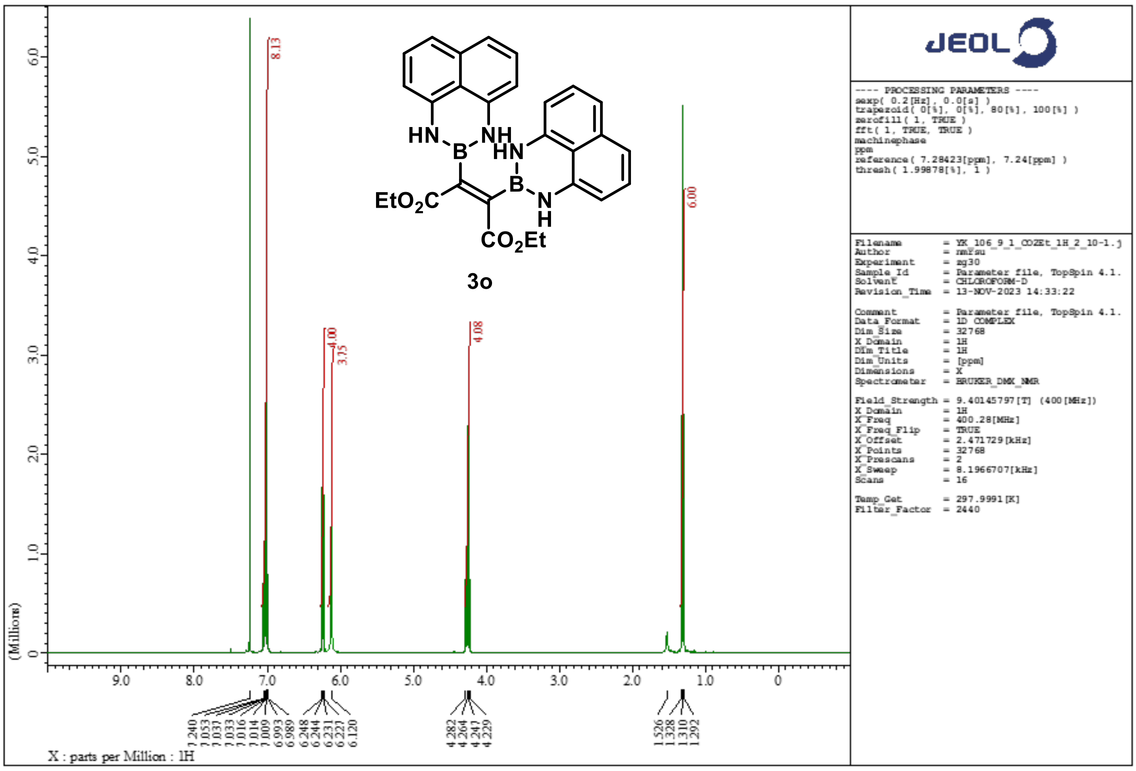

Figure S43. ^{1}H NMR ($CDCl_3$, 400 MHz) spectrum of **3o**.

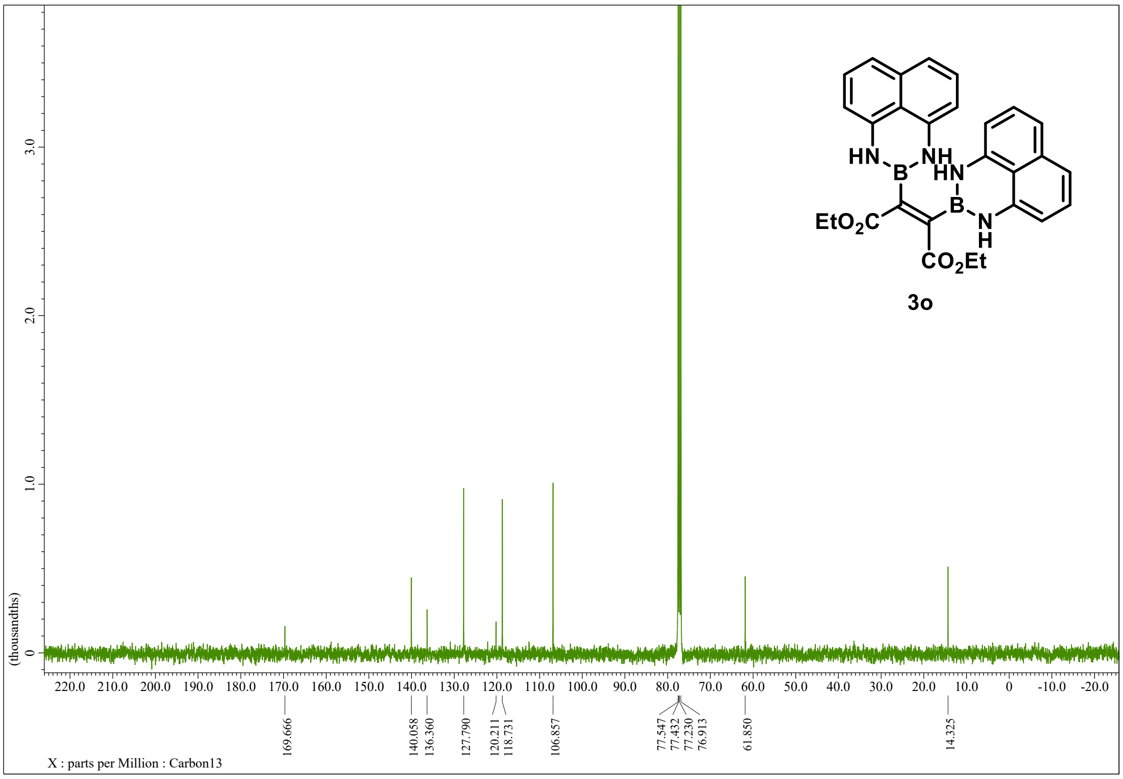

Figure S44. $^{13}C\{^{1}H\}$ NMR ($CDCl_3$, 100 MHz) spectrum of **3o**.

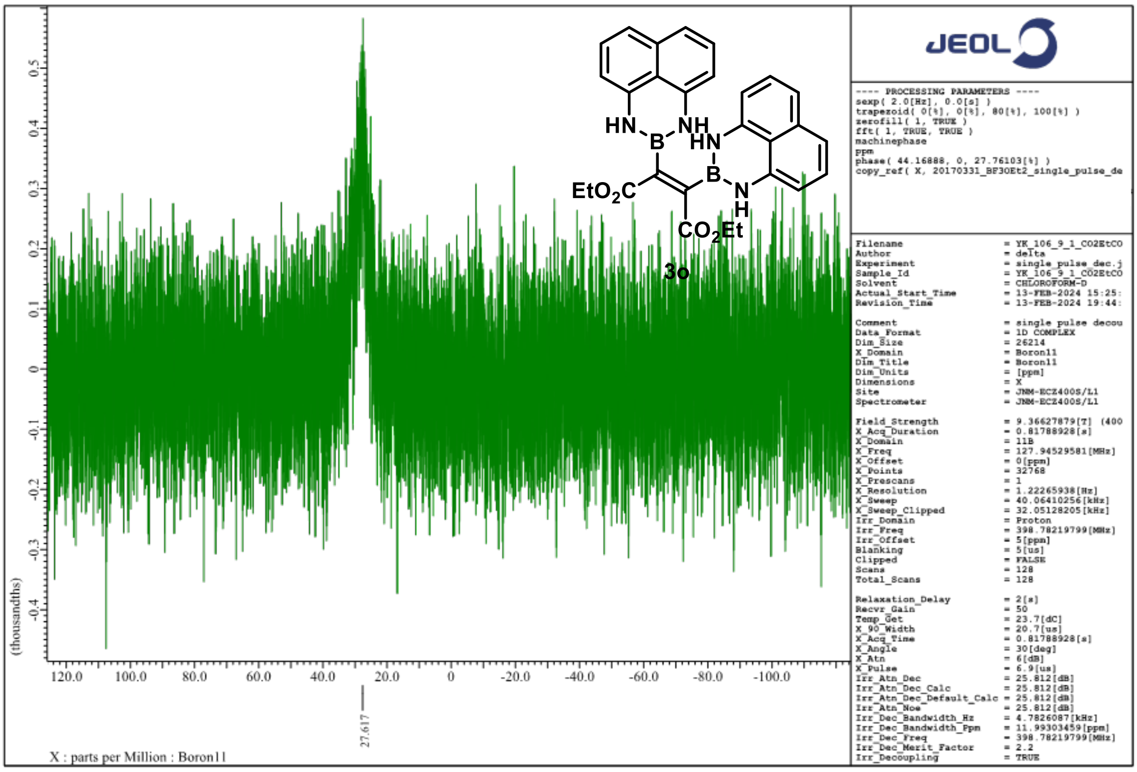

Figure S45. $^{11}B\{^1H\}$ NMR ($CDCl_3$, 128 MHz) spectrum of **3o**.

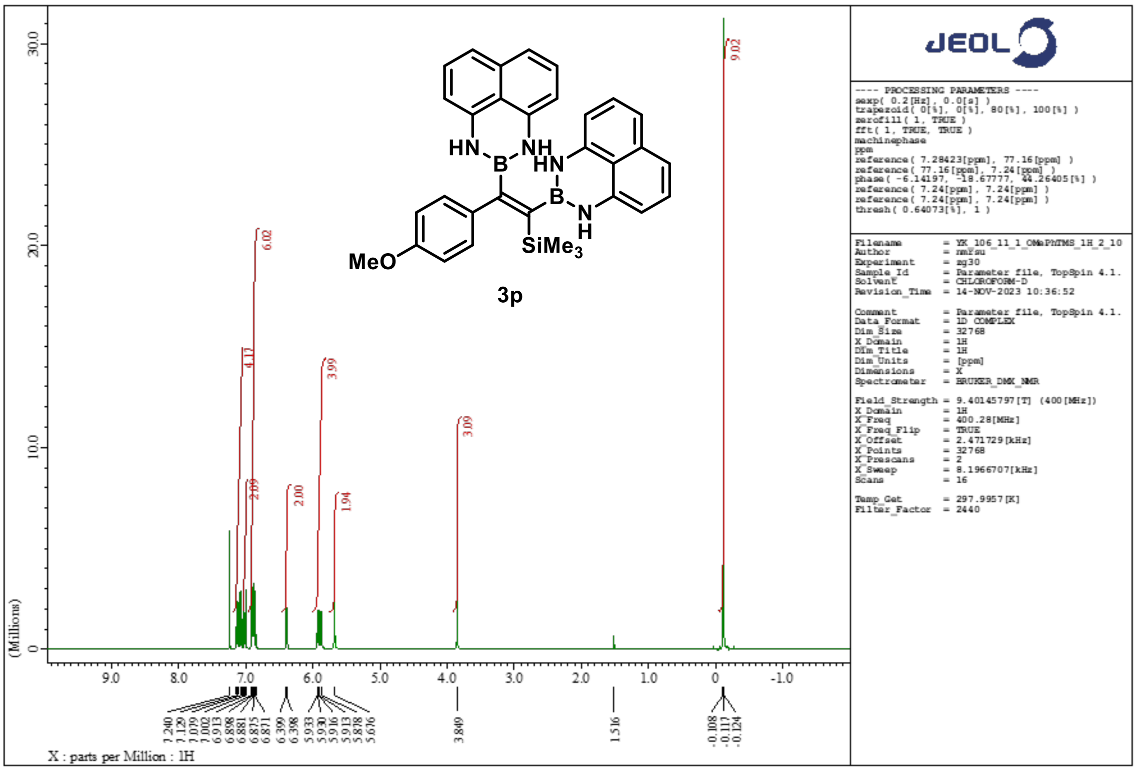

Figure S46. ^{1}H NMR ($CDCl_3$, 400 MHz) spectrum of **3p**.

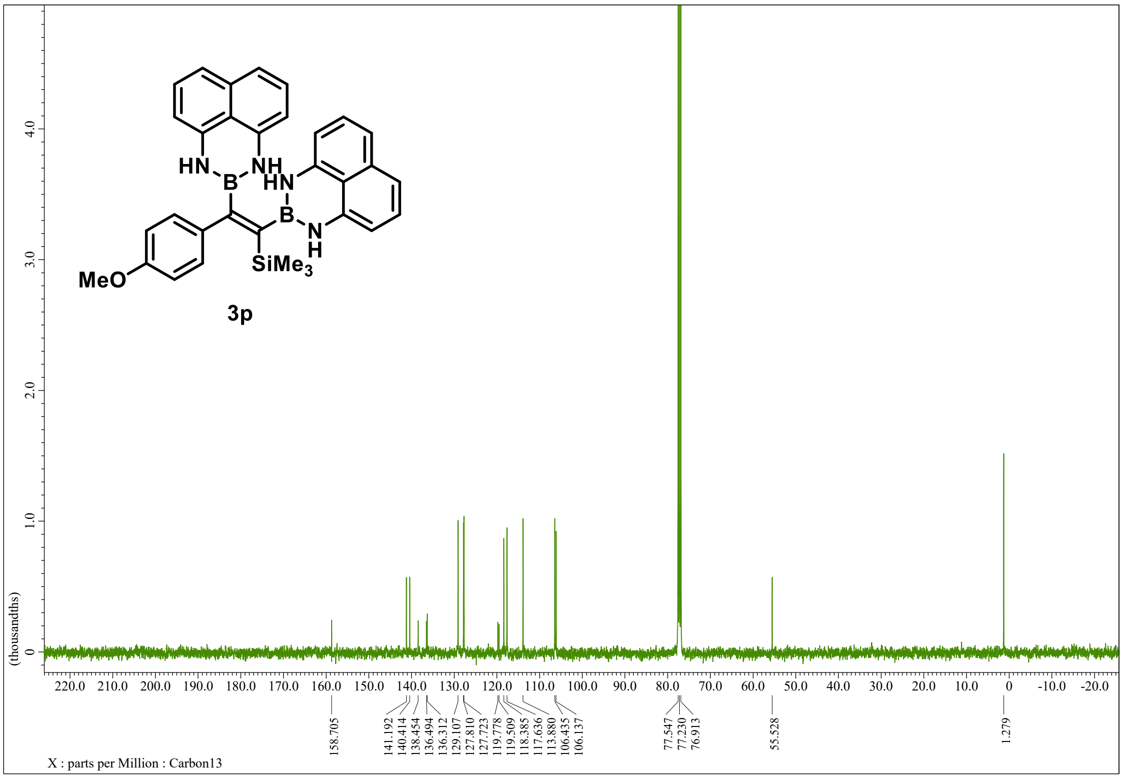

Figure S47. $^{13}C\{^{1}H\}$ NMR ($CDCl_3$, 100 MHz) spectrum of **3p**.

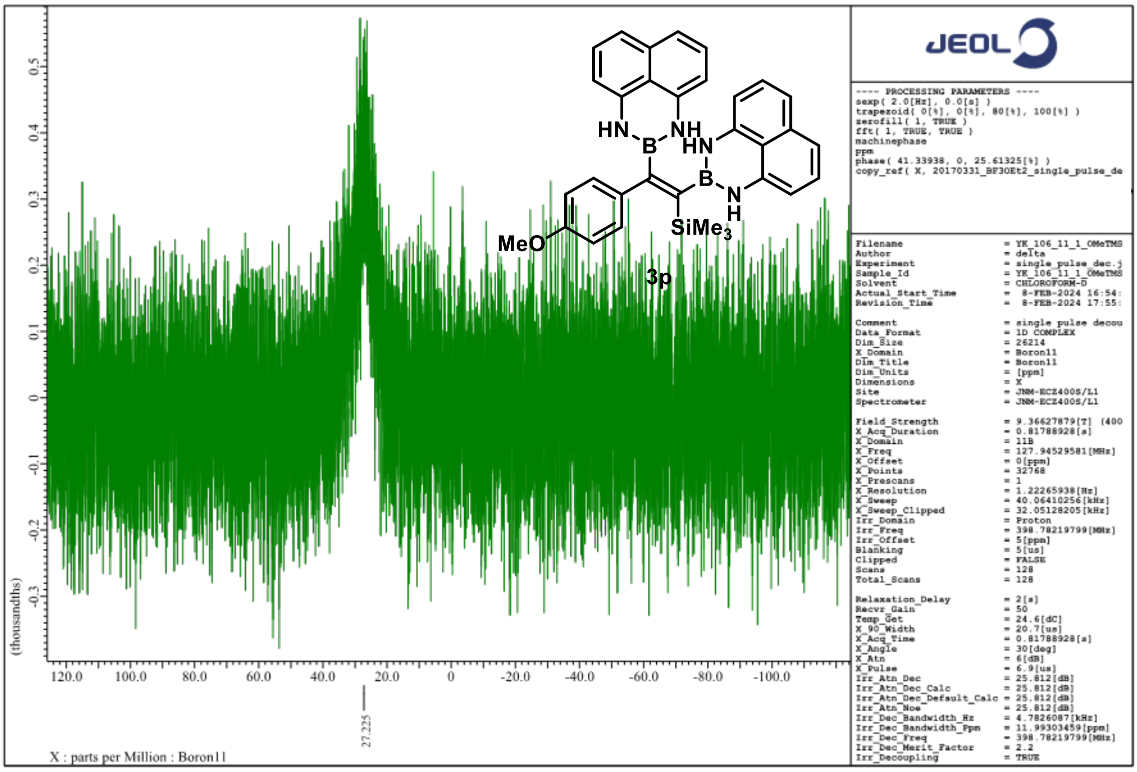

Figure S48. $^{11}B\{^1H\}$ NMR ($CDCl_3$, 128 MHz) spectrum of **3p**.

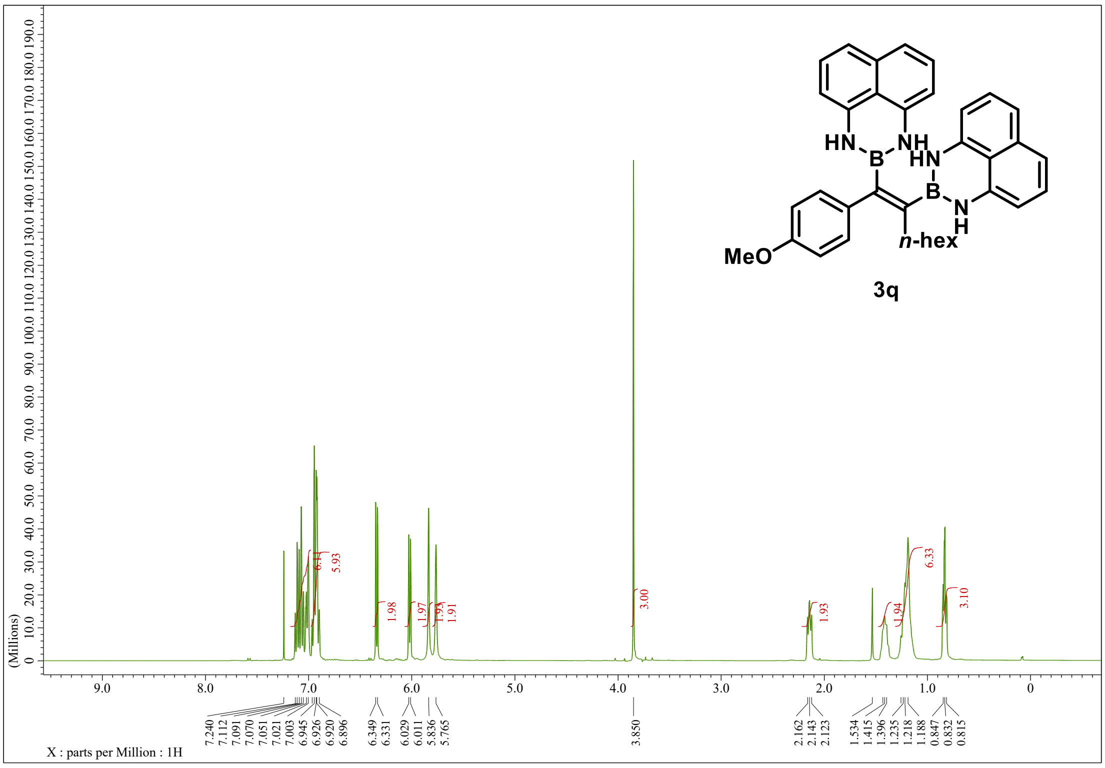

Figure S49. ^{1}H NMR ($CDCl_3$, 400 MHz) spectrum of **3q**.

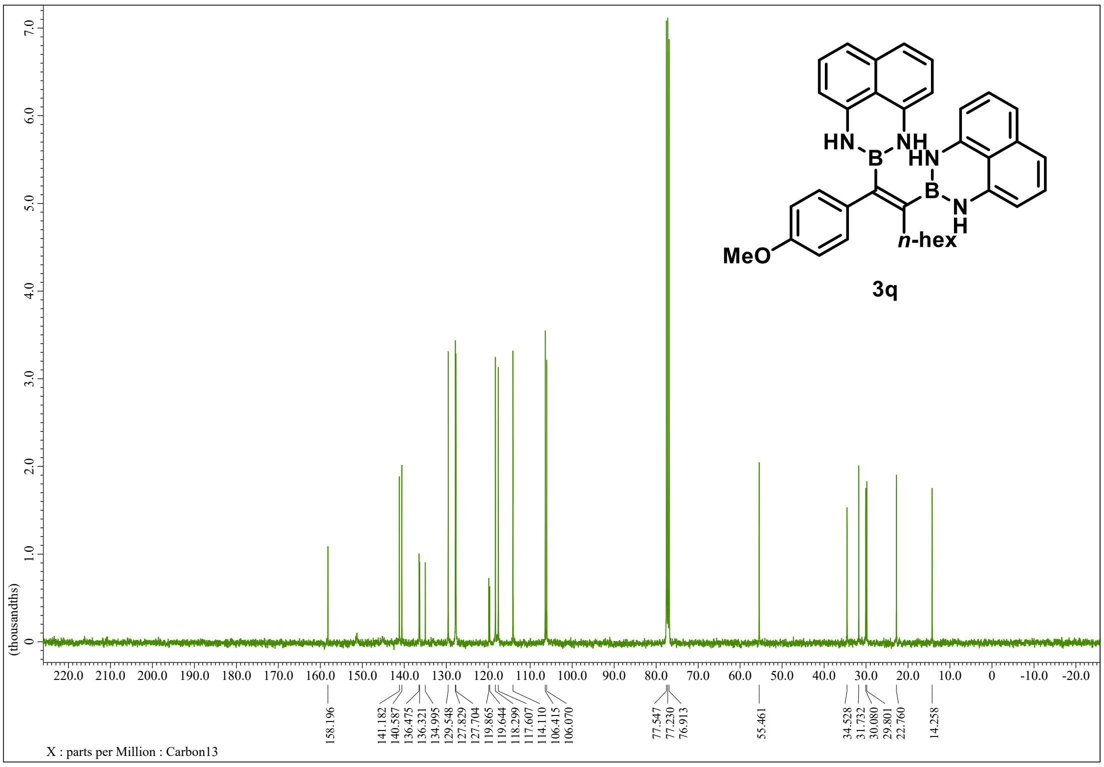

Figure S50. $^{13}C\{^{1}H\}$ NMR ($CDCl_3$, 100 MHz) spectrum of **3q**.

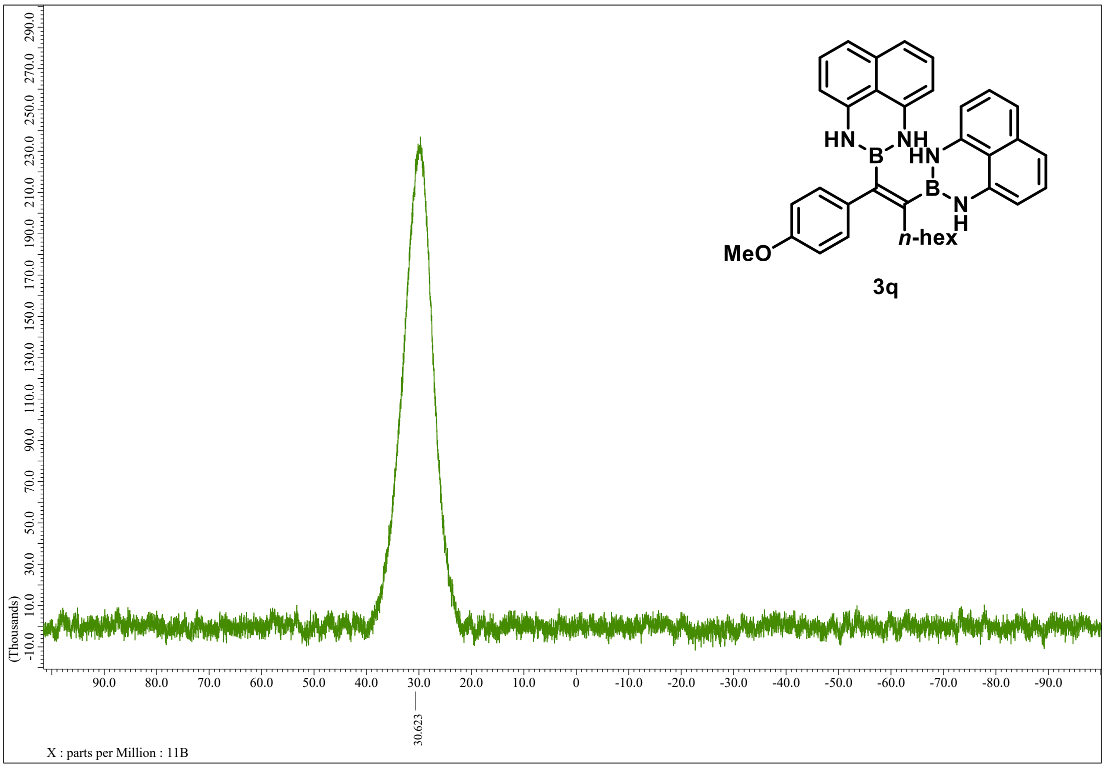

Figure S51. $^{11}B\{^1H\}$ NMR ($CDCl_3$, 128 MHz) spectrum of **3q**.

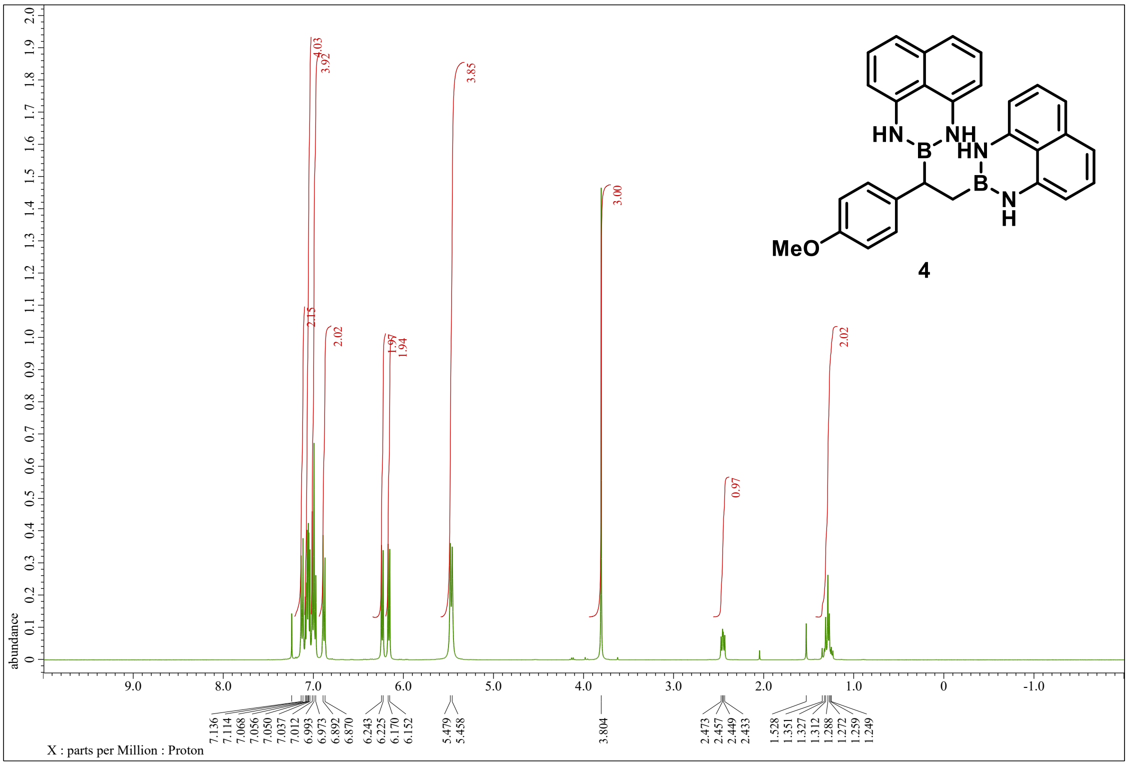

Figure S52. ^{1}H NMR ($CDCl_3$, 400 MHz) spectrum of **4**.

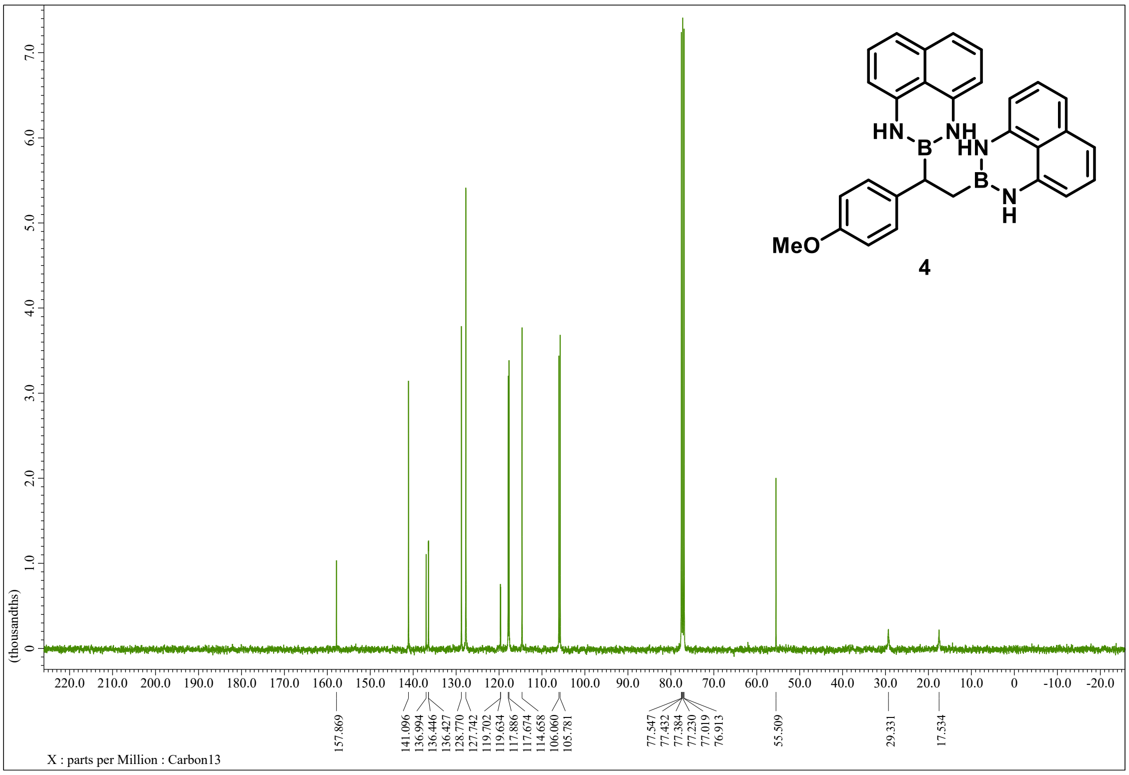

Figure S53. $^{13}C\{^{1}H\}$ NMR ($CDCl_3$, 100 MHz) spectrum of **4**.

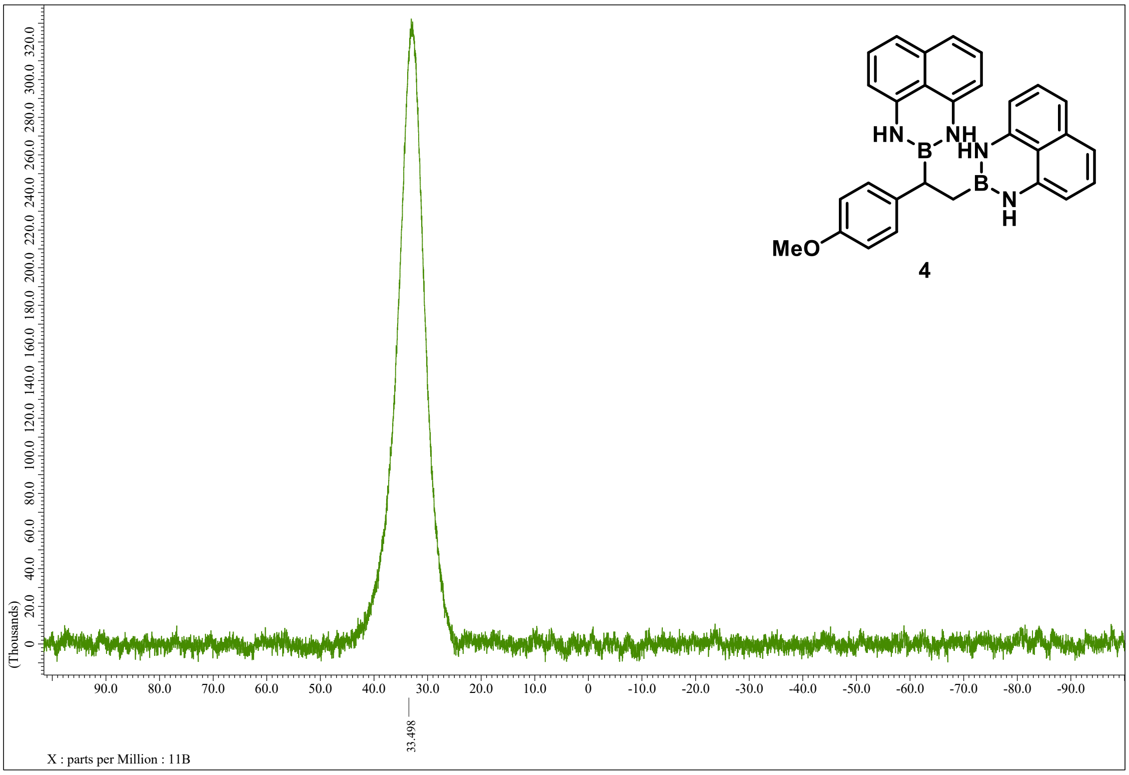

Figure S54. $^{11}B\{^{1}H\}$ NMR ($CDCl_3$, 128 MHz) spectrum of **4**.

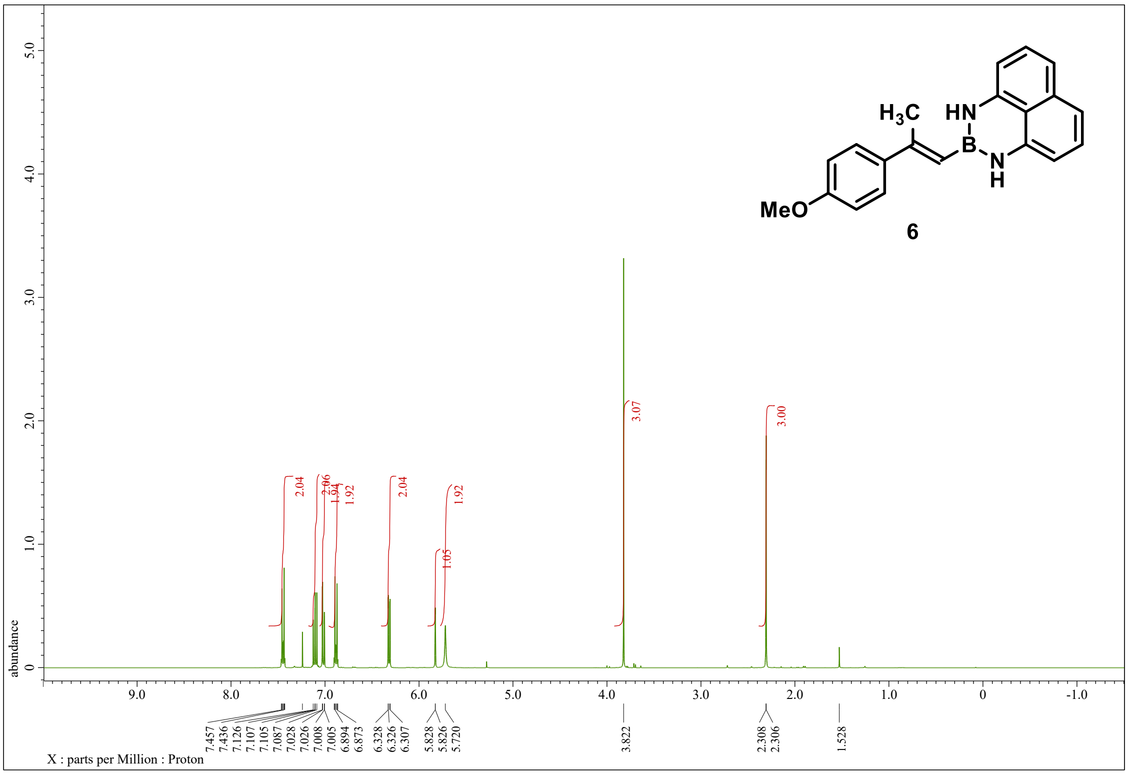

Figure S55. ^{1}H NMR ($CDCl_3$, 400 MHz) spectrum of **6**.

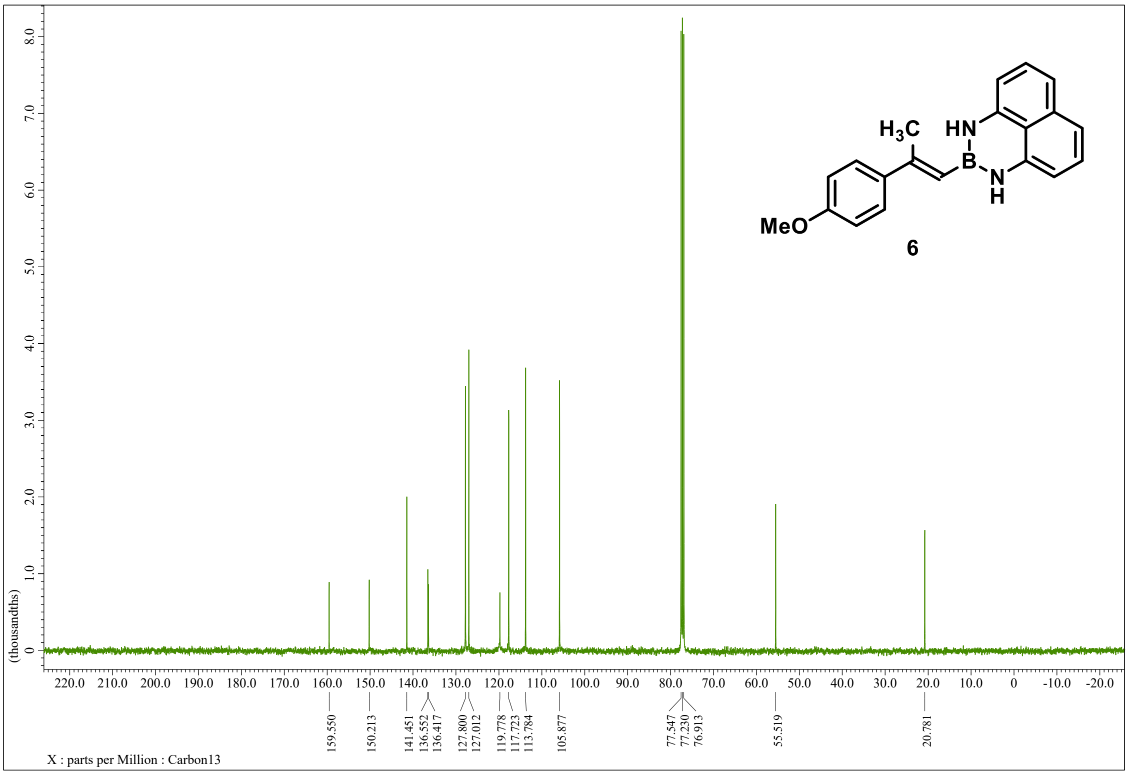

Figure S56. $^{13}C\{^{1}H\}$ NMR ($CDCl_3$, 100 MHz) spectrum of **6**.

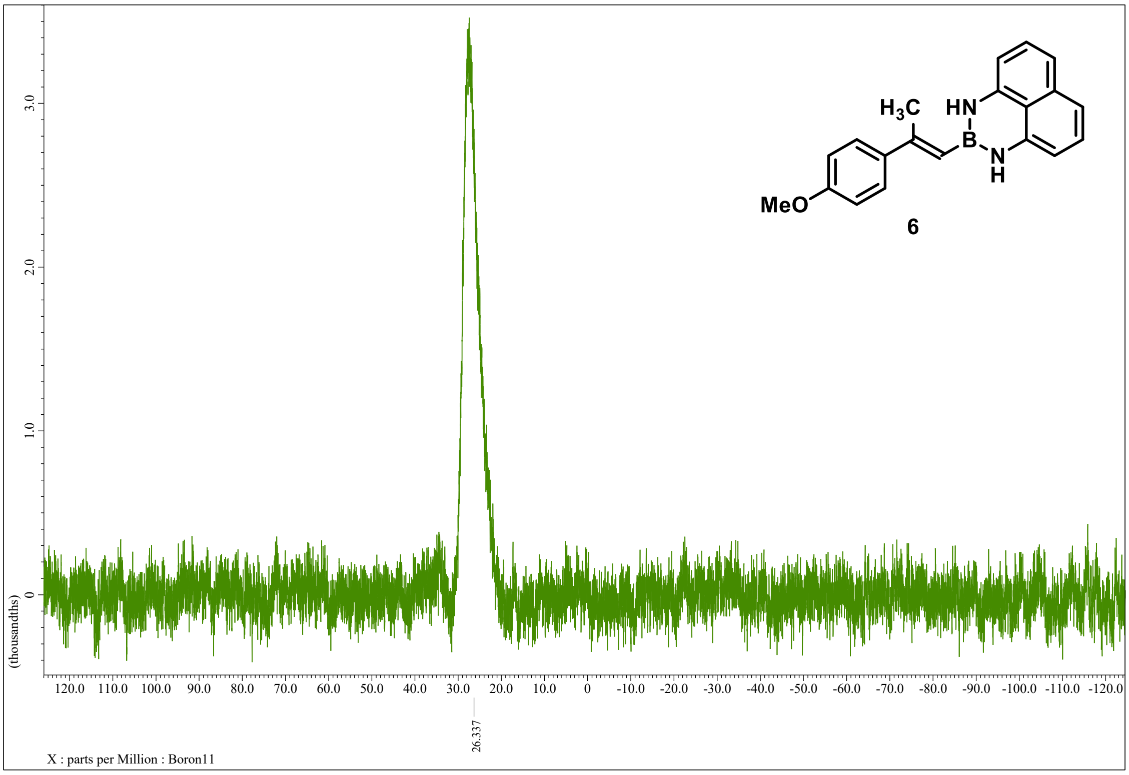

Figure S57. $^{11}B\{^{1}H\}$ NMR ($CDCl_3$, 128 MHz) spectrum of **6**.
